# A *De Novo* Genome Sequence Assembly of the *Arabidopsis thaliana* Accession Niederzenz-1 Displays Presence/Absence Variation and Strong Synteny

**DOI:** 10.1371/journal.pone.0164321

**Published:** 2016-10-06

**Authors:** Boas Pucker, Daniela Holtgräwe, Thomas Rosleff Sörensen, Ralf Stracke, Prisca Viehöver, Bernd Weisshaar

**Affiliations:** 1 Faculty of Biology, Bielefeld University, Bielefeld, Germany; 2 Center for Biotechnology, Bielefeld University, Bielefeld, Germany; Universiteit Gent, BELGIUM

## Abstract

*Arabidopsis thaliana* is the most important model organism for fundamental plant biology. The genome diversity of different accessions of this species has been intensively studied, for example in the 1001 genome project which led to the identification of many small nucleotide polymorphisms (SNPs) and small insertions and deletions (InDels). In addition, presence/absence variation (PAV), copy number variation (CNV) and mobile genetic elements contribute to genomic differences between *A*. *thaliana* accessions. To address larger genome rearrangements between the *A*. *thaliana* reference accession Columbia-0 (Col-0) and another accession of about average distance to Col-0, we created a *de novo* next generation sequencing (NGS)-based assembly from the accession Niederzenz-1 (Nd-1). The result was evaluated with respect to assembly strategy and synteny to Col-0. We provide a high quality genome sequence of the *A*. *thaliana* accession (Nd-1, LXSY01000000). The assembly displays an N50 of 0.590 Mbp and covers 99% of the Col-0 reference sequence. Scaffolds from the *de novo* assembly were positioned on the basis of sequence similarity to the reference. Errors in this automatic scaffold anchoring were manually corrected based on analyzing reciprocal best BLAST hits (RBHs) of genes. Comparison of the final Nd-1 assembly to the reference revealed duplications and deletions (PAV). We identified 826 insertions and 746 deletions in Nd-1. Randomly selected candidates of PAV were experimentally validated. Our Nd-1 *de novo* assembly allowed reliable identification of larger genic and intergenic variants, which was difficult or error-prone by short read mapping approaches alone. While overall sequence similarity as well as synteny is very high, we detected short and larger (affecting more than 100 bp) differences between Col-0 and Nd-1 based on bi-directional comparisons. The *de novo* assembly provided here and additional assemblies that will certainly be published in the future will allow to describe the pan-genome of *A*. *thaliana*.

## Introduction

*Arabidopsis thaliana* was established as a model organism during the last century [1]. Since then, it became the most important model for plant biology [2]. The 119,146,348 bp nuclear genome sequence of the accession Col-0 was published in 2000 [3]. This reference sequence has been generated by the BAC-by-BAC approach and currently represents most probably the plant genome sequence of the highest accuracy. In addition, the functional annotation is of very high quality and usually the source of gene function data which are transferred to other plant genome annotations. However, even this excellent high-quality nuclear genome sequence contains some remaining gaps in repetitive regions like the centromeres [4, 5], telomeres and rDNA regions. The genome annotation in TAIR10 [6], which served as reference for this study, contains 33,323 nuclear genes. Only 81% of these genes (27,206) are protein coding, the remaining 19% are labelled as pseudogenes (924), RNA-coding genes (1,290) or genes of transposable elements (3,903). Functional annotations are assigned to 18,932 distinct genes [7], from which only about 2,400 genes (about 9%) have been experimentally confirmed through molecular complementation or multiple alleles [8]. Moreover, the data set contains 31,189 transposable element entries, which were identified and classified based on the work of Buisine et al. [9]. An update is under way in the context of Araport [10], but the upcoming Araport11 annotation was not finalized when our study was performed.

Besides the ultimate Col-0 reference sequence, to date only few non-reference-guided whole genome sequences of *A*. *thaliana* accessions have been published, most notably that of the popular Landsberg *erecta*-0 (L*er*-0) accession [11]. The L*er*-0 assembly, generated by SMRT (PacBio) sequencing technology, consists of 130.86 Mbp distributed over 545 contigs and was initially provided without structural and functional gene annotation. In addition, several reference-guided assemblies have been made available, for example those of Bur-0, C24, Kro-0 and L*er*-1 [12].

At the beginning of 2008, the 1001 genome project (http://1001genomes.org) was launched in order to determine genome-wide sequence variation between different *A*. *thaliana* accessions by next generation sequencing (NGS) and comparison of the resulting accession-specific sequences to the Col-0 reference. Today, information from about 1,300 different accessions contributes to the very comprehensive set of sequence variation data that is available for *A*. *thaliana* [13]. Within the 1001 genomes project, short sequence variations [12, 14–16] were detected by either mapping of short reads [17] from NGS data of limited coverage, or by reference-guided [12] approaches. Short sequence variations affecting only a few or several nucleotides are referred to as single (or short) nucleotide polymorphisms (SNPs) or as short insertion/deletions (InDels). Such short variations can be detected by mapping of the usually short NGS reads. These data have been exploited intensively to study specific adaptations of local populations, to investigate the sequence diversity within a plant species as well as to carry out genome-wide association studies [18].

Quite significant differences in the genome sizes of different *A*. *thaliana* accessions have been detected by propidium iodide staining of nuclear DNA [19], indicating early on that not only SNPs and InDels contribute to genome variation. The description of the strong contribution of structural variation (SV) to genotype differences in *Z*. *mays* was an important point for explaining genetic diversity in plants more completely ([20] and references therein). SV can be divided into copy number variation (CNV) which refers to all similar sequences with different copy numbers among a set of accessions or genotypes [21], and presence absence variation (PAV). Although most short sequence variations display a length below about 20 bp (see e.g. [22]), we use in this study a length of 100 bp as operational threshold for considering variation as SV. In many cases, CNV is related to additional or removed copies of transposable elements (TEs). In contrast, PAV refers to the presense or absense of unique sequences at a given position in individual genomes among a set of accessions or genotypes [23]. PAV can arise by non-allelic homologous recombination [24], double strand breaks and single strand annealing [25]. In addition, TEs influence the emergence of PAV [26]. Also, full genome- and local duplication events are important for genome evolution and contribute to the basis for selected and random evolutionary changes [27, 28].

Mapping of short reads, even if data from mate pair sequencing is applied, does not allow straightforward identification of SV of unknown sequence [12, 21, 29]. However, identification and description of genotype- or accession-specific sequences is enabled by *de novo* assembly of the genome sequence of new accessions [15, 30]. Access to sequences representing SV in general and specifically PAV is an important step towards the description of the pan-genome of a species. This is of high relevance, because the genome sequence of one accession (or individual) is not sufficient to fully represent the genome structure of a given species. Also, data from many accessions are required to better understand the genetic basis of phenotypes [13, 14]. When attempting to detect PAV by read mapping, genome sequence regions were observed where no sequencing reads derived from another accession of the same species are mapped [23, 31]. Below, we use the term ‘zero coverage region’ (ZCR) for values of the depth of read mapping coverage that falls to zero. There are two possible explanations for such regions: one is the absence of this region in one accession resulting in the detection of PAV, and the other is a high number of sequence differences in the studied region which prevent the reads from mapping. Therefore, these regions have been classified as either PAV or ‘highly diverged region’ (HDR, [32]).

For comparisons at the level of protein coding genes between (closely) related species, the concept of reciprocal best BLAST hits (RBHs) has been widely used to identify homologous or even orthologous genes [33–36]. A RBH pair consists of two protein sequences, one from each species or accession studied, which display the highest scoring genome-wide hit in the other data set in a reciprocal manner [37]. RBH relationships are used to infer orthology between genes, which form the basis for many comparative genomics studies and also analyses of phylogeny. The detection of orthology relationships which are consistent across several or many neighboring genes is referred to as synteny, that is conservation of gene order at the level of genomic blocks, chromosomes or even genomes. Divergence from synteny can, in addition to being very informative for comparative and/or evolutionary genomics, also reveal sequencing artefacts, misannotations or orthology inference errors [38].

In order to overcome the limitations of short read mapping for genome sequence comparison, we assembled the genome sequence of Nd-1 *de novo*. Our Nd-1 sequence assembly was used for a genome wide and systematic identification of SV. Despite the good quality of the Nd-1 genome sequence assembly generated and the relatively low content of repetitive sequences in *A*. *thaliana* genomes, the assembly displays fragmentation in the pericentromeric regions caused by TEs. Therefore, we focused on the analysis of presence-absence variation (PAV) and avoided CNV. Comparisons with the gold-standard Col-0 reference genome sequence let to the identification of several cases of PAV of up to about 13 kbp in size.

## Material & Methods

### Plant material

Niederzenz-1 (Nd-1) seeds were obtained from the European Arabidopsis Stock Centre (NASC; stock number N22619). The *A*. *thaliana* accession Nd-1 is from Germany (Kranz, 1987 #4776). The elevation of the origin is given as 200 m to 300 m above sea level, but there are no coordinates of the exact collection site available. Plants have been grown under short day conditions (8h light, 16h dark) at 21°C for about four weeks prior to extraction of DNA from rosette leaves. Over time, plant material was harvested several times for different NGS technologies (see below). For the Illumina mate pair (MP) libraries generated at the end of the data production phase, plants were etiolated for three days prior to harvest.

### DNA extraction

Genomic DNA for library preparation was extracted from grinded leaf tissue in CARLSON buffer [39] as described in [40]. The DNA was further purified and selected for large molecules via QIAGEN Genomic-tip 20/G according to the suppliers protocol. Genomic DNA for PCR experiments was extracted from *A*. *thaliana* leaf tissue using a cetyltrimethylammonium bromide (CTAB) based method as previously described [41].

### Library preparation and sequencing

Sequence read data from Nd-1 genomic DNA were generated over a period of 8 years. We decided to submit all read data to SRA, including 454 and Ion Torrent runs (S1 Table), although only the more recent high quality data were selected for the final assembly procedure. SNP data deduced from SRX1434931, SRX1434943 and SRX1434944 were contributed to the 1001 Genomes Project (http://1001genomes.org/projects/CeBiTecRies2012).

Library preparation for paired-end (PE) sequencing on Illumina platforms was performed according to the Illumina TruSeq DNA Sample Preparation v2 Guide. DNA was fragmented by nebulization. After end repair and A-tailing, adaptors were ligated to the DNA fragments to allow PE sequencing. Adaptor-ligated fragments were size selected on a two percent low melt agarose gel. Fragments that carry adaptors on both ends were enriched by PCR. Final libraries were quantified using PicoGreen. Average fragment size of the libraries was determined on a BioAnalyzer HighSensitivity DNA chip. A library with about 400 bp insert size was sequenced 2 x 100 nt PE on one lane of a GAIIx run (SRX1434944). An additional PE library with an insert size of 700 to 790 bp was sequenced with 2 x 250 nt on an Illumina MiSeq (SRX1683594).

Nextera MP libraries were constructed according to the Gel-Plus protocol from the Illumina Nextera Mate Pair Sample Preparation Guide. High molecular weight genomic DNA was fragmented and tagged with a junction adapter by mate pair transposomes. Tagmentation-caused gaps were filled. DNA fragments below 1500 bp and components of the strand displacement reaction were removed. Afterwards, fragments were size selected via gelelectrophoresis and purified via Zymo Purification kit (Zymo Research) to increase the proportion of large DNA molecules (6-12 kbp). Resulting molecules were circularized using biotinylated adaptors. Removal of linear fragments was achieved by DNA exonuclease treatment. Circular molecules were randomly fragmented by nebulization. Biotinylated fragments were purified using streptavidin-coated magnetic beads. The following steps were carried out as described for the PE library construction. Finally, samples were sequenced on an Illumina MiSeq generating 2 x 250 nt MP reads (SRX1434948, SRX1683821). After completion of the Illumina sequencing runs, basecalling, demultiplexing and fastq file generation was performed using a CASAVA-based inhouse script.

### Trimming of sequencing reads

Trimmomatic [42] was applied for adaptor removal, quality and length trimming of the Illumina PE data. The minimal required sequencing read length was set to 36 nt and unpaired reads were removed. NxTrim [43] was applied with default parameters on the Nextera MP reads for trimming and extraction of PE and MP data. NxTrim separates PE and MP read pairs when analyzing the sequence data generated from a MP library. The extracted PE reads can, in addition to the mate pair data, be used in downstream applications.

### Sequence read based estimation of genome size

Data from run SRX1434948 were used to estimate the genome size of Nd-1. Jellyfish2 version 2.3 [44, 45] was applied with -m = 25. The total area under the peak of the most frequent k-mer 23 was determined. Normalization to the most frequent k-mer revealed the genome size. The size of the nucleolus organizing region (NOR) was calculated by estimation of the number of NOR repeats (or 45S transcription units) in Nd-1. Genomic reads were mapped to three adjacent copies of a manually assembled NOR repeat, and the resulting average read coverage was used to deduce the NOR repeat copy number. The difference between the estimated genome size and the sum of assembly plus NOR size was assumed to represent centromeric sequences. An equal distribution of these sequences over all five centromeres was assumed.

### Assembly parameters

Our *A*. *thaliana* Nd-1 *de novo* sequence was computed using CLC genomics workbench (v. 8.0, CLC bio) and data from three sequencing runs (SRX1434944, SRX1434948 and SRX1683821). The *de novo* assembly pipeline was applied with automatic detection of best parameters. After the assembly process, all sequencing reads were remapped to all contigs to update the assembly. In accordance with good practice, all contigs below a length threshold of 500 bp were removed. Resulting contigs were scaffolded by SSPACE [46] using PE (SRX1434944, SRX1683594) and MP (SRX1434948, SRX1683821) data with slightly modified default settings (S2 Table). Gaps between contigs inside of scaffolds were closed by GapFiller [47] with mostly default settings in nine iterations (S3 Table). In order to remove plastid and mitochondrium DNA sequences, the scaffolds were mapped to the Col-0 reference sequence using BLAT [48] (option settings: -extentThroughN and -fine). All scaffolds without good hits against the nucleome, but with good hits against the plastome [GenBank: AP000423.1] or chondrome [GenBank: Y08501.2] were removed. Therefore, nucleome-derived sequences with similarity to plastome or chondrome ("numts") remained in the assembly. Sequences with over 40% content of ambiguity characters were removed. All remaining scaffolds with a match against Col-0 below 50% of their length were checked for bacterial origin. These sequences were subjected to BLASTn [49] against the NCBI non-redundant nucleotide database nt. Scaffolds were removed if they matched synthetic DNA (e.g. vector), sequences from a number of species (listed in S4 Table). These sequences were probably derived from barcoded libraries that were sequenced in our sequencing core facility in parallel to the Nd-1 libraries, and ended up in the data due to failure of the de-multiplexing for a few reads.

The remaining scaffolds were sorted into five groups based on their chromosomal location deduced from BLAT and BLAST mapping to the Col-0 reference sequence. Unmapped scaffolds were placed in a separate group. Sequences with assigned chromosomal location were oriented according to the Col-0 reference sequence. All unmapped sequences remained as produced by the assembler. The SEC10 locus (At5g12370 [50]) and the NOR region in the north of chromosome two and four were manually corrected and integrated into the scaffolds. In addition, scaffolds were manually broken if analyses of read mapping data indicated bad scaffolding. RBH synteny analysis was used to collect hints for such positions, essentially by attempting to validate each hint for true differences in gene order between Nd-1 and Col-0. Assembly regions showing deviating RBH neighbours were checked and revealed very often assembly errors. This iterative manual procedure of RBH synteny analysis, validation of scaffolding by mapping PE and MP reads (S1 Table), and re-scaffolding was repeated several times until no additional artificial deviations were detected. An AGP file (S1 File) describes the final followup of sequences within the assembly. The statistics of the assembly that deviated from the CLC output after manual improvement were determined with a dedicated Python script.

### Assembly validation

After the internal validation by CLC we applied REAPR [51] with default parameters on different assembly versions from before and after manual RBH-based optimization. MP reads of SRX1683821/2x250 were mapped with CLC, parameters 'length fraction' and 'similarity fraction' were set to 0.9 and 0.95, respectively.

### Scaffold and exon mapping

Mapping of all scaffolds to the Col-0 reference sequence for quality assessment was carried out by BLAT (parameters: -fine and -extendThroughN). Positions of the Col-0 reference sequence to which scaffold ends mapped were checked for TEs in the Col-0 annotation dataset. In addition, 2 kbp of the most outer sequence of all scaffolds with sufficient length (i.e. more than 4 kbp) were mapped to the Col-0 reference sequence and these positions were also checked for TEs.

Col-0 exon sequences from the TAIR10 transcriptome dataset were detected in the Nd-1 assembly via BLASTn with an e-value cutoff of 0.01. As a control, Col-0 exon sequences were also checked by BLAST against the Col-0 reference with the same parameters. Only hits in Nd-1 with at least 50% of the score achieved in a BLASTn against the Col-0 reference sequence were considered. Only one hit was allowed per genome sequence region.

### AUGUSTUS gene prediction

AUGUSTUS 3.2 [52, 53] was applied to the Nd-1 assembly. *Ab initio* gene prediction was carried out using the optional parameters - -species = arabidopsis - -gff3 = on - -uniqueGeneId = true - -codingseq = on. For further analysis sequences were extracted by the Perl script getAnnoFasta.pl (http://bioinf.uni-greifswald.de/augustus/binaries/scripts/).

### EST mapping

A total of 1,664 ESTs from the accession Nd-1 are available from GenBank (GenBank accessions CB259106 to CB260427 and CF651219 to CF651560 [54]). All ESTs were filtered for quality and length (minimal length of 300 bp), low sequence quality and high sequence similarity (at least 95% query coverage) to the *A*. *thaliana* plastome (GenBank: AP000423.1) as well as chondrome (GenBank: Y08501.2). The remaining 1,325 ESTs covering 892 different genes were aligned to the Nd-1 assembly using BLAT. ESTs were considered to be 'mapped' if the alignment covers at least 95% of the query sequence with at least 90% identity.

### Sequencing read mapping and identification of ZCRs

The 'map reads to reference' method of CLC genomics workbench was applied to map Nd-1 sequencing reads from SRX1434944, SRX1434948 and SRX1683821 (138 million reads) to the Col-0 reference sequence, and Col-0 sequencing reads (SRX879613 [55]; 24 million reads, 2 x 250 nt) to the Nd-1 assembly. The parameters 'length fraction' and 'similarity fraction' were set to 0.9 and 0.95, respectively. Non specific matching reads were mapped randomly to detect only PAV. However, changing the mapping mode to "unique" did not significantly influence the results. Default settings for all other parameters were applied. Based on the read mapping graph, regions without any coverage were identified (S1 Fig). The lower cutoff size for the selection of zero coverage regions (ZCRs) was set to five base pairs. Identified ZCRs were regarded as candidates for deletions in the genome of the accession from which the sequencing reads were derived. Simultaneously, ZCRs are candidates for insertions in the genome that was used as target for mapping. ZCRs were ignored if they contained more than 10% of ambiguity characters. For further categorization, sequences of 1 kbp flanking ZCRs on both sides were subjected to BLASTn against the assembled genome sequence of the read source accession. Results were screened for adjacent hits in the expected orientation, and the distance between the inner ends of the hits was calculated for each hit pair. Directly adjacent BLASTn hits on the same scaffold supports the absence of the ZCR between the two flanking sequences, and we considered the respective region as PAV. A lower limit of 100 bp was implemented for PAV to distinguish PAV from InDels, and to compensate for small inaccuracies of position determination by BLASTn in diverged regions. For ZCRs larger than 1 kbp, the tolerance for the distance between the two BLAST hits was increased to 10% of the length of the ZCR. ZCRs that did not qualify as PAV by the BLASTn analysis were considered as HDRs. To validate that our ZCR detection relied on sufficient coverage, we mapped fractions of increasing size of the Col-0 sequencing reads to the Nd-1 assembly. Number and size of identified variants were plotted against the amount of sequencing read coverage, and the result showed that the coverage was in the plateau region (S5 Table). The PAV candidates detected were checked *in silico* by analyzing the distance of mapped read pairs at the specific location. An deviation of 100 bp compared to the average mapping distances was used as cutoff.

### Identification of SNPs and InDels

Mapping of sequencing reads was done as described above. The 'basic variant detection' and ‘structural variant detection’ methods of CLC were used to identify SNPs and InDels, respectively. Mapping data was exported in BAM format for application of GATK 3.4 [56–58] with the goal to improve InDel detection by applying IndelRealign as well as for SNP detection. The BAM file was sorted and indexed by PicardTools v.1.119 (https://github.com/broadinstitute/picard). Afterwards, hard filtering was applied on SNPs (QD<2.0, FS>60.0, MQ<40.0) and InDels (QD<2.0, FS>200.0, DP>300, DP<30) separately. Only variants that were detected by both methods were considered. The findings were collected in VCF format, and the file is available upon request.

### Application of SnpEff

SnpEff 4.1 [59] was called on the filtered set of small variants using the TAIR10 annotation as reference. In principle, deletions in the CDS were classified by SnpEff as ‘high’, codon changes as moderate, and synonymous variants were expected to have a ‘low’ impact. Effects in intergenic regions were classified as ‘modifier’. Finally, SnpEff assigns one or more effects to each small variant. We filtered the predicted effects based on their impact. Only one predicted effect per variant was taken into account for further analysis. When projecting effects of variants to genes, the most relevant effect of the major transcript was extracted to place genes in only one of the categories "premature stop", "lost stop", "splice site variant" or "frameshift". Effects downstream of a premature stop codon were ignored. Premature stop codons were manually checked for second site variants which could revert the first variant effect.

### Identification and visualization of reciprocal best hits (RBHs)

All protein sequences predicted by AUGUSTUS based on the Nd-1 genome sequence were subjected to a BLASTp against the Col-0 protein sequences of TAIR10 [6]. Protein sequences encoded on the plastome or chondrome were excluded, as well as those with the TAIR10 annotation ‘transposable element’. Reciprocally, the Col-0 protein sequences were subjected to BLASTp analysis against the predicted Nd-1 protein sequences. The e-value cutoff was set to 0.001 and maximal target number was set to one. All Col-0 RBH genes were sorted according to their position in the reference sequence. Predicted Nd-1 genes were sorted in the same way based on the ordered and oriented scaffolds. Afterwards, the positions in these lists were identified for each RBH pair and visualized in a dot plot via an inhouse Python script. Outliers were further analysed by checking for multiple perfect BLASTp hits of the involved protein sequences. They were designated as “random” outliers if they show multiple good hits or as "real" outliers if there was only one good BLASTp hit. Centromere positions were taken from TAIR10 [6] and validated by location and abundance of the 180 bp centromeric repeat [60–62]. The consensus sequence of the 180 bp repeat (which actually has a length of 179 bp) was obtained from PlantSat (http://w3lamc.umbr.cas.cz/PlantSat/family.php?dir=Arabidopsis_thaliana_180 [61]).

### Read coverage analysis of genic sequences

Read mapping was carried out as described above for ZCR detection. The average read coverage of all genic sequences was calculated. Predicted Nd-1 genes and the annotated Col-0 genes were used for this purpose. The coverage values were plotted, yielding a very constant value of 112.4 with a standard deviation of only 1.1 fold for the genic sequences contributed by the RBH gene set.

### PCR

Standard *Taq* DNA polymerase was applied for PCRs on genomic DNA. Flanking oligonucleotides were manually designed for the generation of amplicons spanning the predicted site of the addressed deletion or insertion. If the candidate PAV was larger than 5 kbp, additional primers were used to address the predicted borders of the PAV (see S2 Fig). Amplicons were analyzed by gel-electrophoresis and visualized with ethidiumbromide. PCR products were purified by ExoSAP-IT^®^ (Affymetrix) prior to Sanger sequencing using BigDye terminator chemistry on an ABI Prism 3730x sequencer (Applied Biosystems, Foster City, CA, USA).

## Results

### Nd-1 genome sequence

Data from different NGS sequencing technologies were generated over time for the Nd-1 genome, yielding a total coverage of 218x (assuming about 150 Mbp genome size). A subset with a coverage of about 120x (18.14 Gbp) was used for creating the assembly (see S1 Table). All read data have been submitted to SRA (linked to PRJNA302255). Several assembly attempts were carried out, usually after addition of new data. Finally, the best assembly resulted from omitting older sequencing data, and by relying only on recent Illumina data from three runs for the CLC assembly as well as using additional MP data for scaffolding. To assign and anchor the scaffolds to positions along chromosomes, we replaced genetic mapping by sequence mapping using BLASTn and BLAT. Chromosome positioning and scaffolding was further improved by an iterative process that relied on RBH synteny and inspection of synteny breakpoints indicated by lack continuity of RBH succession (see below and Material & Methods). The resulting *A*. *thaliana* Nd-1 *de novo* assembly comprised 117 Mbp (Table 1) and has been deposited at DDBJ/ENA/GenBank under the accession LXSY00000000. The version described in this paper is version LXSY01000000.

**Table 1:** Assembly statistics. Metrics of the Nd-1 genome sequence assembly before and after application of SSPACE, GapFiller and subsequent RBH-based manual improvement.

parameter	CLC assembly	scaffolded	gaps filled	polished
number of scaffolds	10,057	5,201	5,201	5,197
total number of bases	113,939,710	117,144,260	117,816,107	116,846,015
average scaffold length	11,329 bp	22,523 bp	22,652 bp	22,483 bp
minimal scaffold length	500 bp	500 bp	500 bp	500 bp
maximal scaffold length	445,914 bp	3,176,818 bp	3,190,961 bp	2,967,516 bp
GC content	35.98%	35.98%	35.95%	35.95%
N25	102,863 bp	1,299,823 bp	1,304,062 bp	1,211,412 bp
N50	52,252 bp	709,626 bp	713,021 bp	589,639 bp
N75	22,586 bp	214,378 bp	215,617 bp	174,007 bp
N90	7,163 bp	42,960 bp	43,285 bp	40,994 bp

**Fig 1:**
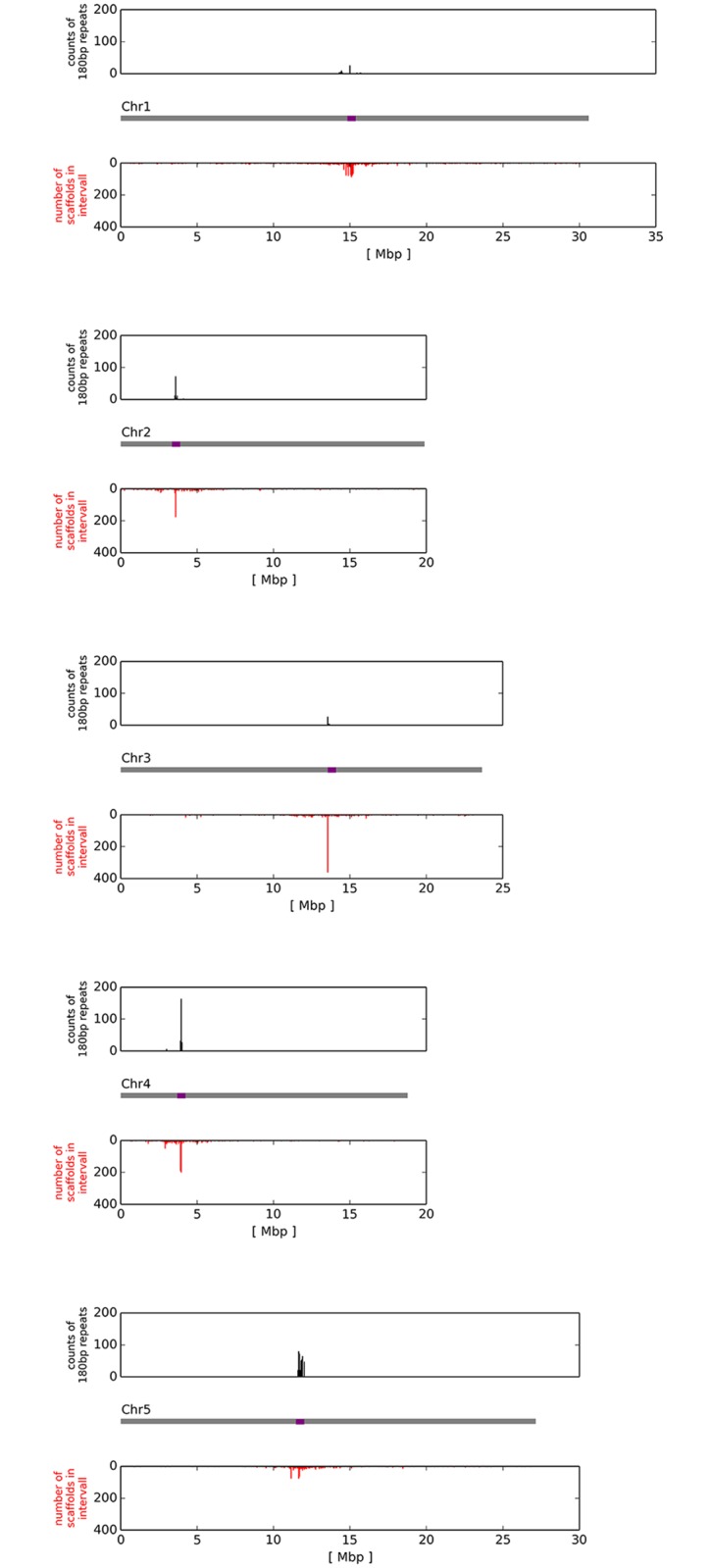
Mapping of Nd-1 scaffolds to Col-0 reference sequence. Schematic chromosomes are shown in grey with centromere positions in purple. Below each chromosome, red bars indicate the frequency of scaffolds. Above each chromosome, black bars show the abundance of the 180 bp centromeric repeat that has been shown to be a major component of *A*. *thaliana* centromeric DNA [62]. Data were calculated for a window size of 50 kbp.

Despite the total number of 5,197 scaffolds, an N50 of 0.59 Mbp and a longest scaffold of almost 3 Mbp in length indicate a high continuity and quality of the assembly. Assembly quality was further analyzed by mapping all scaffolds to the Col-0 reference sequence. In total, 5,086 (98%) of the scaffolds were successfully assigned to the pseudochromosomes of Col-0, covering about 99.8% of the Col-0 reference sequence. A total of 153 regions in Col-0 without Nd-1 scaffold coverage cumulate to only 267 kbp in length. The ten chromosome arms were covered with few and long scaffolds, while shorter and many more scaffolds are clustered around the centromere positions (Fig 1). In fact, 2,866 scaffolds are located within one Mbp of the fife centromere positions (adding up to 10 Mbp), while the remaining 2,220 scaffolds cover 110 Mbp of the "genic" Col-0 genome sequence. Analysis of the sequence and the relative location of scaffold ends indicated that about 65% of the Nd-1 scaffolds end within TEs annotated in the Col-0 reference.

### Nd-1 genes detected by *ab initio* gene prediction

An *ab initio* gene prediction using AUGUSTUS and the *A*. *thaliana* training set on the Nd-1 assembly resulted in 28,670 nuclear protein coding genes with an average gene length of 2,124 bp, an average CDS length of 1,625 bp and an average exon number per gene of four. Gene prediction with the same parameters on the Col-0 nuclear reference sequence resulted in 27,862 protein coding genes, which exceeds the number given in the TAIR10 annotation (27,206 nuclear protein coding genes) by only 656.

### Detection of strong synteny between Nd-1 and Col-0

The predicted Nd-1 and Col-0 TAIR10 peptide sequences were compared by BLASTp in both directions revealing 22,178 reciprocal best hits (RBHs). The RBH set represented 81.5% of the 27,206 nuclear Col-0 genes. Average read coverage over all genic sequences of the RBH set (22,178 genes) in Nd-1 was 112.4x ± 1.1x (S3 Fig). Coverage analysis of the 6,492 (predicted) genes from Nd-1 not included in the RBH set identified 1,216 genes with a read coverage very similar to the average of 112.4x ± 1.1x. Almost all the 5,276 non-RBH genes were clearly separated in the coverage distribution and showed an at least two fold increase in read coverage. We interprete this increased read coverage for the majority of non-RBH genes as an indication for collapsed sequences derived from e.g. transposable elements.

**Fig 2:**
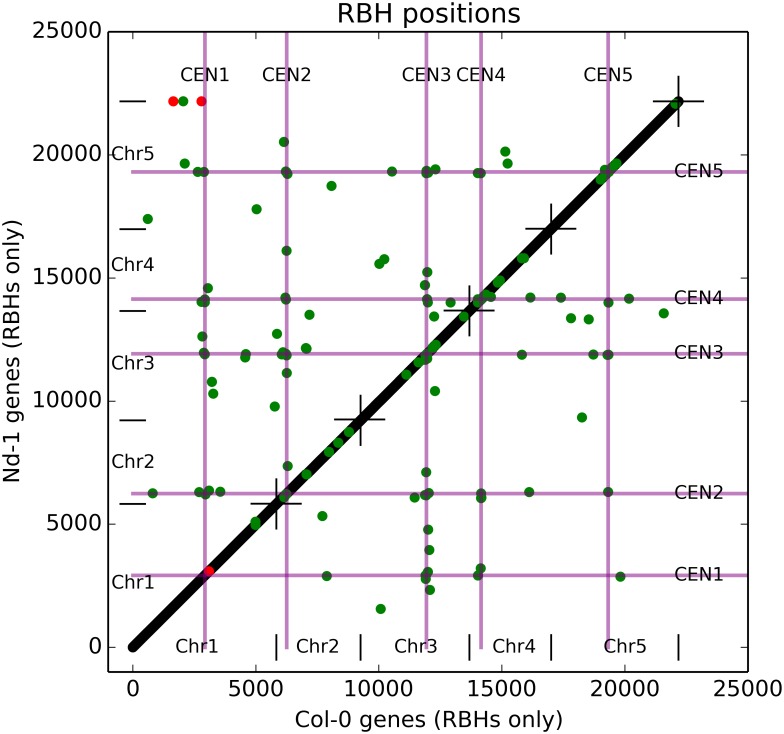
Reciprocal best hits (RHB) synteny of Nd-1 and Col-0. All five pseudochromosomes of the two genome sequences were ordered by their number to provide the x (Col-0) and y (Nd-1) axes of the diagram. Positions of each RBH pair in the two genome assemblies were plotted, resulting in a bisecting line formed from black dots representing perfectly matching RBH pairs. RBH gene pair positions deviating from a fully syntenic position, i.e. the outliers, are represented by green dots for RBH pairs with ambiguous best hits in RBH pair identification, and by red dots for RBH pairs with deviating (non-syntenic) gene positions. Since two red dots overlap each other, only three locations are visible. Positions of the centromeres (CEN1 to CEN5) are indicated by purple lines. Ends of pseudochromosomes (telomers) are indicated by short black lines at the bisectrix (forming crosses) and on both axis. Formally, the unmapped fraction of Nd-1 contigs is appended after pseudochromosome 5, but this sequence of about 134 kbp in length becomes invisible due to the limited resolution of the figure.

Colinearity analysis of the genomic positions of the 22,178 RBHs (see S2 File for a list) between Nd-1 and Col-0 revealed a very high synteny of both genomes (Fig 2). While nearly all RBHs are properly preceeded and followed by their syntenic homologs and therefore result in a diagonal line in the plot, there are exactly 200 outliers. These were separated into 196 “random” outliers (green), which have multiple equally good BLASTp hits for genes at different genomic positions, and four “real” outliers (red), which have only a single good BLASTp hit. “Random” outliers occur most often in pericentromeric regions.

### Comparison of the exon and gene space of Nd-1 and Col-0

As a first step and also to further validate the assembly, the set of 1,325 Nd-1 ESTs was mapped to the Nd-1 genome sequence. A total of 96% of the ESTs mapped to 902 different loci in the genome sequence, supporting the completeness of the assembly.

The TAIR10 Col-0 annotation contains 206,604 nucleome exons with a sequence length above 50 bp. In order to investigate the completeness of the Nd-1 gene space, all of these Col-0 exons were subjected to a BLASTn search against the Nd-1 assembly. In total, 203,634 (98.6%) of the Col-0 exons were identified in the Nd-1 assembly. The undetected 1,389 exons belong to 1,246 different genes, and most of these genes are annotated as either TE genes (771), genes with unknown function (126) or pseudogenes (77).

### Assembly validation with REAPR

Almost complete coverage of the Col-0 reference sequence and the detection of most exons indicated a high completeness of the Nd-1 assembly. REAPR reported 44 errors within the initial CLC assembly. After scaffolding with SSPACE an increased value of 383 errors was reported. Manual splitting of scaffolds at 166 critical positions lead to the final assembly version. REAPR still reports 254 errors, but the error locations clustered in the pericentromeric regions, were in conflict with SSPACE and do not affect syntenic gene order.

### Nd-1 genome size

Based on k-mer analysis of raw sequencing read data the total Nd-1 genome size was estimated to 146 Mbp (see Methods). The most abundant repeat in *A*. *thaliana*, the 180 bp centromeric repeat, was identified in the Nd-1 assembly. Based on read coverage, this repeat sequence appears about 60,000 times in the Nd-1 genome which should contribute about 11 Mbp to a complete genome sequence. However, the Nd-1 assembly contains only 2,575 copies of this repeat (Fig 1), and in the Col-0 reference includes 799 copies. Also based on read coverage, the copy number of nucleolus organizing region (NOR) repeats which encode the 45S rRNA transcription unit amounts to about 400 and results in a total NOR size of about 4 Mbp. Taken together, the assembly with a total length of 117 Mbp misses about 29 Mbp of sequence of which 25 Mbp most probably belong to centromeres and telomeres. Assuming equal size of the five centromeres, each might cover about 4.5 Mbp of which one half is represented by the 180 bp centromeric repeat. Sequence variation in the form of InDels, PAV and CNV (see below) within the mapped scaffolds also contributes to differences in the assembly sizes of Nd-1 and Col-0, but only to a quite minor extend.

### Small variants

High quality Illumina reads of Nd-1 were mapped to the Col-0 reference sequence for identification of small sequence variation. A total of 485,887 variations including 410,158 SNPs were identified (S3 File). Among the SNPs, the number of transitions is higher than the number of transversions. The average genomic SNP and InDel variation frequency was one in 244 bp. The variation frequency ranged from one in 285 bp on chromosome 5 to one in 223 bp on chromosome 4 (S4 Fig). Taking only SNPs into account, the total frequency was one SNP in 292 bp. As expected, these SNPs were found predominantly in non-coding sequences (1 in 234 bp) and less often in protein coding sequences (1 in 524 bp). Length of InDels in coding sequences is more often a multiple of three than in non-coding sequences (S5 Fig). SNPs identified of this work matched 76% of the SNPs in the data set of Illumina reads submitted in 2012 to the 1001 genomes website.

The effects of the 485,887 small variants were predicted and categorized according to their importance using SnpEff (see Methods). The impact of 2,388 variants (0.5%) was classified as ‘high’, the impact of 38,106 variants (7.9%) was classified as ‘moderate’, the impact of 52,813 variants (10.9%) was classified as low, and the impact of the remaining 390,939 variants (80.7%) was designated as ‘modifier’. High impact variants were extracted (S6 Table). Premature stop codons were predicted in 314 genes, lost stop codons in 117 genes, splice site variants in 71 genes, and frameshifts in 1,228 genes.

### PAV and HDRs

Mapping of Nd-1 reads against the Col-0 reference sequence was used to identify ‘zero coverage regions’ (ZCRs). In total, 9,558 ZCRs larger than 100 bp were detected in Col-0; the largest displayed a size of 53 kbp (Table 2, S6 and S7 Figs). The median ZCR size was 237 bp summing up to a cumulative total length of about 5.5 Mbp for all ZCRs. The distance between sequences flanking ZCRs in the Col-0 reference sequence was used to distinguish between HDRs and PAV (see Methods and S1 Fig). In total and by focusing on Nd-1, we detected 746 deletions (S7 Table) relative to Col-0 with a median size of 325 bp and a cumulative length of 626.5 kbp. The remaining 8,812 ZCRs with a minimal length of 100 bp and a cumulative length of 4.9 Mbp, were considered as HDRs, comprising about 4% of the Col-0 genome sequence.

**Table 2:** Summary of the sizes of large insertions, deletions and HDRs. The data were compiled from reciprocal read mapping of Nd-1 reads to the Col-0 genome sequence and vice versa. However, the table presents the results regarding PAV from the view of Nd-1; an insertion in Nd-1 is at the same time a deletion in Col-0, and a deletion in Nd-1 is at the same time an insertion in Col-0.

Variant length [bp]	ZCRs (Col-0 reads)	Insertions in Nd-1	ZCRs (Nd-1 reads)	Deletions in Nd-1
**100–200**	3,331 (480,416 bp)	244 (34,606 bp)	4,021 (569,529 bp)	227 (31,974 bp)
**201–400**	2,817 (794,403 bp)	220 (60,698 bp)	2,644 (738,991 bp)	207 (58,734 bp)
**401–800**	2,112 (1,196,028 bp)	140 (79,879 bp)	1,461 (808,370 bp)	121 (67,725 bp)
**801–1600**	1,141 (1,281,170 bp)	106 (118,182 bp)	775 (862,766 bp)	99 (110,558 bp)
**1601–3200**	631 (1,416,843 bp)	42 (92,912 bp)	380 (834,816 bp)	41 (91,758 bp)
**3201–6400**	411 (1,857,365 bp)	57 (264,498 bp)	211 (946,860 bp)	42 (195,585 bp)
**6401–12800**	119 (1,029,274 bp)	15 (116,191 bp)	57 (469,079 bp)	8 (56,713 bp)
**12801–25600**	25 (410,562 bp)	2 (26,505 bp)	4 (61,067 bp)	1 (13,487 bp)
**>25600**	3 (103,639 bp)	-	5 (206,506 bp)	-
**Total:**	**10,590 (8,569,700 bp)**	**826 (793,471 bp)**	**9,558 (5,497,984 bp)**	**746 (626,534 bp)**

The other way around, sequence reads from Col-0 were used to identify ZCRs in the Nd-1 genome sequence. In total, there were 10,590 ZCRs in the Nd-1 assembly with a median size of 313 bp summing up to a cumulative length of 8.6 Mbp. There were 826 insertions larger than 100 bp with a maximum length of 39 kbp, a median size of 321 bp and a cumulative length of 793 kbp (Table 2). Moreover, there are 9,764 HDRs with a minimal length of 100 bp and a cumulative length of 7.8 Mbp. This comprises about 6.5% of the Col-0 reference sequence. Detected PAV and HDRs were equally distributed over the five chromosomes (S8 Table).

One of the identified PAV depicts a known 6.8 kbp deletion of the flowering locus M in the Nd-1 genome [63], validating the results obtained. PAV candidates were *in silico* checked using distance deviations of mapped read pairs. This check supported 1,130 of the 1,572 PAV candidates (Table 2; S7 Table). For further validation, we experimentally confirmed randomly selected cases of PAV with a range of different sizes (from 0.9 kbp to 13.5 kbp) in the Nd-1 genome. Primers that anneal to regions flanking the individual PAV were used to amplify the region of interest from Col-0 and Nd-1 (S9 Table). In total, 24 of the 31 experimentally addressed PAV predictions were confirmed. The set included 13 deletions (0.96 kbp– 5.6 kbp, all confirmed) and 18 insertions (0.95 kbp– 13.5 kbp, 11 confirmed) in Nd-1.

## Discussion

In order to address the occurrence of large variations between *A*. *thaliana* accessions, we performed a *de novo* NGS-based genome assembly for the Nd-1 accession using up-to-date assembly and scaffolding technologies for short read assemblies.

### Genome sequence of the *A*. *thaliana* accession Nd-1

Mapping of all Nd-1 NGS sequencing reads to the Col-0 reference sequence revealed that 96% of the reference genome sequence was covered. This is in the same range reported for reference-guided assemblies in an earlier study with four other *A*. *thaliana* accessions [12]. Also reads from the unmapped fraction were incorporated in scaffolds during the assembly procedure. The scaffolds themselves span smaller and larger regions with higher divergence to Col-0 where individual Nd-1 reads did not map to the Col-0 genome sequence. All assembled Nd-1 scaffolds cover about 99.8% of the Col-0 reference sequence. The remaining differences could be traced back mainly to the pericentromeric regions. This is also indicated by the highly increased fragmentation of the Nd-1 assembly close to the centromeres (Fig 1). Similar differences between Col-0 and 20 other accessions of about 4% have been reported before on the basis of read mapping analyses [64]. Moreover, the current Col-0 reference sequence does not cover the entire genome. There are a few remaining known gaps in the sequence caused by highly repetitive regions that are difficult to assemble, including the centromeres [3–5]. This might cause misplacement of scaffolds in the Nd-1 assembly, because potentially correctly assembled parts of Nd-1 centromeric sequences cannot find there homologous sequence from Col-0 and end up at more or less random positions in the pericentromeric region.

### Nd-1 genome size

We used the frequency of the appearance of the 180 bp *A*. *thaliana* centromeric repeat to confirm positioning of the centromeres in Nd-1, and also for genome size calculations. Large arrays of centromeric 180 bp repeats comprise a huge fraction of *A*. *thaliana* centromere sequences [65]. These repeats are a key component of *A*. *thaliana* centromeres [62] which are usually operationally defined by the presence of CenH3 [62, 66, 67]. At the DNA sequence level, transposable elements and other repeats are located in *A*. *thaliana* centromeric regions in addition to the 180 bp repeat [68, 69].

Another fraction of the genome that contributes a significant part of sequence length is the NOR, that is the array of rDNA repeats encoding the 10 kbp long 45S transcription unit [70]. Our CLC assembly did not contain a correct rDNA repeat sequence. This was probably due to the very high read coverage of slightly divergent sequences and was solved by manual assembly of the rDNA repeat unit. In the final Nd-1 assembly, the NOR region is represented by three copies of this manually assembled repeat in the north of chromosome 2 and in the north of chromosome 4. Based on read coverage, we estimated the number of NOR repeats in Nd-1 to about 400. This is lower than the 570–750 copies reported before for Col-0 [71], and also lower than the total NOR size of the 7–8 Mbp reported before for L*er* [72].

Considering assembly size of mainly genic sequences of 117 Mbp and a k-mer based total size estimation of 146 Mbp, the difference of 29 Mbp is well explained by NOR sequences (4 Mbp) and 5 centromeres of 4.5 to 5 Mbp each. This derived average Nd-1 centromere size matched previous results [4, 5, 73].

### *De novo* assembly approach

Early versions of the Nd-1 genome assemblies did also include data from 454 (SRX1434931) and Ion Torrent (SRX1434934) sequencing runs as well as older short Illumina reads (SRX1434943, SRX1434947). However, during optimization of the assemblies and selection of the best input data these older sequence data were excluded. Inclusion of these data did not contribute to increased assembly quality but increased the run time of the assembly process significantly. The main contribution to the quality of the Nd-1 assembly are the 2 x 100 nt PE as well as the 2 x 250 nt PE and MP data, supported by improved assembly and scaffolding technologies. Together, this allowed the *de novo* genome sequence assembly of an *A*. *thaliana* accession, which was not satisfyingly possible five years ago [12].

The unequal size distribution of Nd-1 scaffolds with shorter sequences covering pericentromeric regions is due to varying content of repetitive sequences across the genome. This conclusion is also supported by the fact that many scaffold ends fall into sequences annotated as TEs. This pinpoints to the limits of short read assemblies for eukaryotic genomes even if mate pair or jumping libraries have been included. A correlation between presence of TEs and assembly breaks has previously been reported [74, 75]. Assemblies based on long read technologies like Single Molecule, Real Time (SMRT) sequencing are required to reach significantly better assembly quality [76].

The presented Nd-1 *de novo* assembly is fully independent of the Col-0 sequence and allows analyses of SV. However, due to the fact that TEs limit scaffold length, we had to exclude CNV from our analyses because TEs are responsible for a large part of CNV. On the other hand, while searching for differences in gene order between Nd-1 and Col-0 based on RBH [37] relations, all candidate cases of genome rearrangements detected in pre-final versions of the assembly were caused by assembly errors. Almost all of these errors were caused by over-scaffolding, resulting in wrongly positioned contigs due to overruling of the position of the tagged-on contig by the main part of the scaffold. Splitting of scaffolds that contained bad joins and re-positioning of the resulting parts produced a significantly improved assembly. The result shown in Fig 2 indicates the synteny between the two closely related genotypes. Such RBH-based assembly polishing, which is based on synteny of gene order, could also be used for other *de novo* assemblies with available reference sequence.

### Small variants and their effect

The detected SNP frequency between Col-0 and Nd-1 of one SNP in 292 bp is in the same range as reported between other *A*. *thaliana* accessions (e.g. between Bur-0 and Col-0 with one variant in 199 bp and between Bur-0 and Col-0 with one variant in 227 bp [32]) and for other plant species (e.g. SNP frequency in different apple strains varies between one in 378 bp and one in 186 bp [77]). Explanations for the higher number of transitions compared to transversions are spontaneous deaminations of methylated cytosines, leading to thymine substitutions [78, 79] or ultraviolet light-induced mutagenesis [80]. However, the ratio between transitions and transversions is not as high as described before in the comparison of five *A*. *thaliana* lines after 30 generations [81]. Selection against a strong increase in AT content could be one reason for the observed difference.

As expected, the SNP frequency within coding sequences is lower than in the whole genome sequence, because selection slows down the accumulation of mutations inside CDS. Also distribution of InDels varies between coding and non-coding sequences displaying clear signatures of selection. InDels with lengths of three or multiples of three were detected more often within coding sequences than InDels of other lengths, because multiples of three do not disturb the reading frame [82].

Within the 1,700 predicted high impact effects (S6 Table), three previously known, biological relevant variants were recovered. Variations leading to nonsense-mutations in *MYB114* (At1g66380 [83]) and *BGLU6* (At1g60270 [84]) were detected properly, validating the method applied. Moreover, a predicted lost stop codon in *RRS1* (At5g45260) led to a longer protein sequence in Nd-1. This locus was previously studied by genetic mapping approaches [85, 86]. The small variant causing the lost stop codon results in resistance of Nd-1 against *Ralstonia solanacearum* [87] while Col is sensitive.

### Presence/absence variation (PAV)

The 1,572 individual cases of PAV detected between Nd-1 and Col-0 (S7 Table) is only a lower limit because there might be additional deletions in Nd-1 that escaped the BLASTn hit distance check of flanking sequences. This could be due to their location at the end of or between scaffolds. In addition, highly diverged flanking sequences could prevent the detection of deletions by BLASTn. The average sizes of identified insertions and deletions were larger than previously reported for a comparison of Col-0 and the reference-guided Ler-1 assembly [12]. Again, this is most probably caused by improved read length and reduced error rate of the 2 x 250 nt PE and MP data in our analysis compared to 40 nt or 80 nt in former analysis [32, 64]. Due to the differences in the data source in terms of read length, quality and coverage, conclusions about the phylogenetic distance between the three accessions, Col-0, Ler-1 and Nd-1 are not appropriate. It should also be noted that numbers and sizes of PAV detected in different studies must, for the reasons mentioned above, be compared with caution. Nevertheless, a Nd-1-specific deletion of 6.8 kbp that removes the entire transcribed region of the *FLOWERING LOCUS M (FLM*, At1g77080), leading to early flowering phenotype under short-day conditions [63], was among the detected deletions.

The results of the experimental validation of predicted PAV indicate the proper identification of the majority of the 1,572 cases. However, the number of PCR-confirmable predicted deletions in Nd-1 was higher. This could be due to the higher amount of sequencing reads of Nd-1 which were used for the identification of ZCRs in Col-0. We validated 24 out of 31 candidates experimentally and interpreted the PCR results conservatively, meaning that experimental contributions to negative results like primer failure or fade fragments increased the ‘failed’ fraction.

One remarkable difference between the Nd-1 assembly and the Col-0 reference sequence is the *SEC10* locus (At5g12370), which was described as a hidden error in the Col-0 reference sequence previously [50]. *SEC10* was assembled to two copies in Nd-1, but is collapsed into a single copy in the Col-0 reference sequence. This results in a case of CNV between Nd-1 and the Col-0 reference sequence. We attempted to use this case for validation of methods to use read coverage for the detection of duplicated genes, but the noise in the sequence read data was too high for reliable results.

## Conclusion

We report the first whole genome *de novo* assembly of the *A*. *thaliana* ecotype Niederzenz-1 (Nd-1). Comparison of the Nd-1 gene set with the Col-0 reference genes revealed 22,178 RBHs which were used to optimize scaffolding of the Nd-1 genome sequence based on synteny. The assembly was used to study PAV between Nd-1 and Col-0 on a genome scale. We provide a structural gene annotation suitable for analyzing the CDS of genes in the assembly for variants. Our work contributes to the emerging *A*. *thaliana* pan-genome by adding new sequences that were not known from the Col-0 reference sequence.

We believe that the results generated in this study could be improved if a less fragmented genome sequence becomes available. Data from long sequence read technology and scaffolding information like those obtained from SMRT sequencing [11] and optical mapping [88, 89] will be required to generate such superior genome sequence assemblies.

## Supporting Information

S1 Fig Concept of ZCR validation by BLASTn. Sequencing reads of one accession (Col-0 in the example shown) were mapped to the genome sequence of the other accession (here Nd-1). ZCRs were identified from the read coverage graph. Flanking sequences of ZCRs were subjected to BLASTn against the genome sequence of the read source accession. Adjacent BLASTn hits in correct orientation confirm the absence of the ZCR in the genome that provided the reads, and indicates PAV between the two genomes studied.
(TIF)

S2 Fig Concept of experimental validation of insertions in Nd-1. The concept is visualized by using a PAV of about 13 kbp in length that is present in Nd-1 and absent from Col-0 as an example. This figure shows the primer positions used for experimental validation (bottom). Outer primers (Nd66 and Nd67) were used for standard PCR on genomic DNA of Col-0 and Nd-1 (gel picture of amplicons, top left) and for long range PCR on genomic DNA of Nd-1 (top right). Inner primers were used for amplicon generation in standard PCR with genomic DNA of Nd-1.
(JPG)

S3 Fig Average read coverage in predicted Nd-1 genes. Nd-1 sequencing reads were mapped to the assembly. The average coverage within predicted genes is 112x +/- 1.1x. The average coverage inside of RBHs (blue) and inside of non-RBHs (green) is shown.
(PNG)

S4 Fig Genome wide distribution of small variants. Numbers of SNPs (black) and InDels (red) in a given interval on the chromosomes are shown. Both variant types were identified using GATK and CLC genomics workbench as described in the method section. The overlap of both tools was considered as the best choice.
(PNG)

S5 Fig InDel size distribution. Most frequent InDel sizes differ between coding and non-coding regions. Multiple of three are much more common in coding sequences.
(PNG)

S6 Fig Genome wide distribution of ZCRs. ZCRs identified via mapping of Nd-1 reads to the Col-0 reference sequence are shown.
(PNG)

S7 Fig Genome wide distribution of PAV. Only ZCRs with expected PAV as cause of the missing read coverage are shown.
(PNG)

S1 File Nd-1 AGP file. The Nd-1 scaffolds are sorted into five pseudochromosomes and ‘Random’.
(AGP)

S2 File RBH pairs. The identified RBH pairs between Col-0 and Nd-1 are listed.
(TXT)

S3 File Summary of SnpEff results. This file was constructed by SnpEff while processing the detected variants as a summary.
(HTML)

S1 Table NGS data overview. Sequencing data produced for the Nd-1 *de novo* assembly are listed. Use of the data for the assembly, scaffolding or submission for documentation as example case only is indicated.
(XLSX)

S2 Table SSPACE options. Options are listed, if they were changed from default.
(XLSX)

S3 Table GapFiller options. Options are listed, if they were changed from default.
(XLSX)

S4 Table Sequence types and species designations for removal of contaminations. All scaffolds, which matched sequences with the listed terms in their annotation were removed from the assembly. We expect these sequences to be derived from previous or parallel sequencing projects in our sequencing core facility.
(XLSX)

S5 Table Effects of different read coverages on detected SV. Detected SV per relative coverage of all Col-0 reads are listed.
(XLSX)

S6 Table High impact small variants. Small variants annotated by SnpEff as ‘stop_gained’, ‘splice_region_variant’ or ‘frameshift’ are listed.
(XLSX)

S7 Table Predicted PAV. Positions of predicted PAV between Col-0 and Nd-1 are listed.
(XLSX)

S8 Table Distribution of variants over Col-0 chromosomes. Positions of insertions, HDRs and deletions in Nd-1 were associated with one of the Col-0 chromosomes. The number and the cumulative length of the events per chromosome were calculated.
(XLSX)

S9 Table Experimentally confirmed PAV. ZCRs identified via read mapping indicated PAV. Candidates shown here were randomly selected from this data set. PCR amplification of the region of interest was used to confirm PAV.
(XLSX)

## Abbreviations

CNV: copy number variants
HDR: highly diverged regions
NOR: nucleolus organizing region
RBH: reciprocal best hit
ZCR: zero coverage region
