# Supplementary figures and images for: A De Novo Genome Sequence Assembly of the Arabidopsis thaliana Accession Niederzenz-1 Displays Presence/Absence Variation and Strong Synteny
Source: PLoS One. 2016 Oct 6;11(10):e0164321. doi: 10.1371/journal.pone.0164321 (PMC5053417; doi:10.1371/journal.pone.0164321)

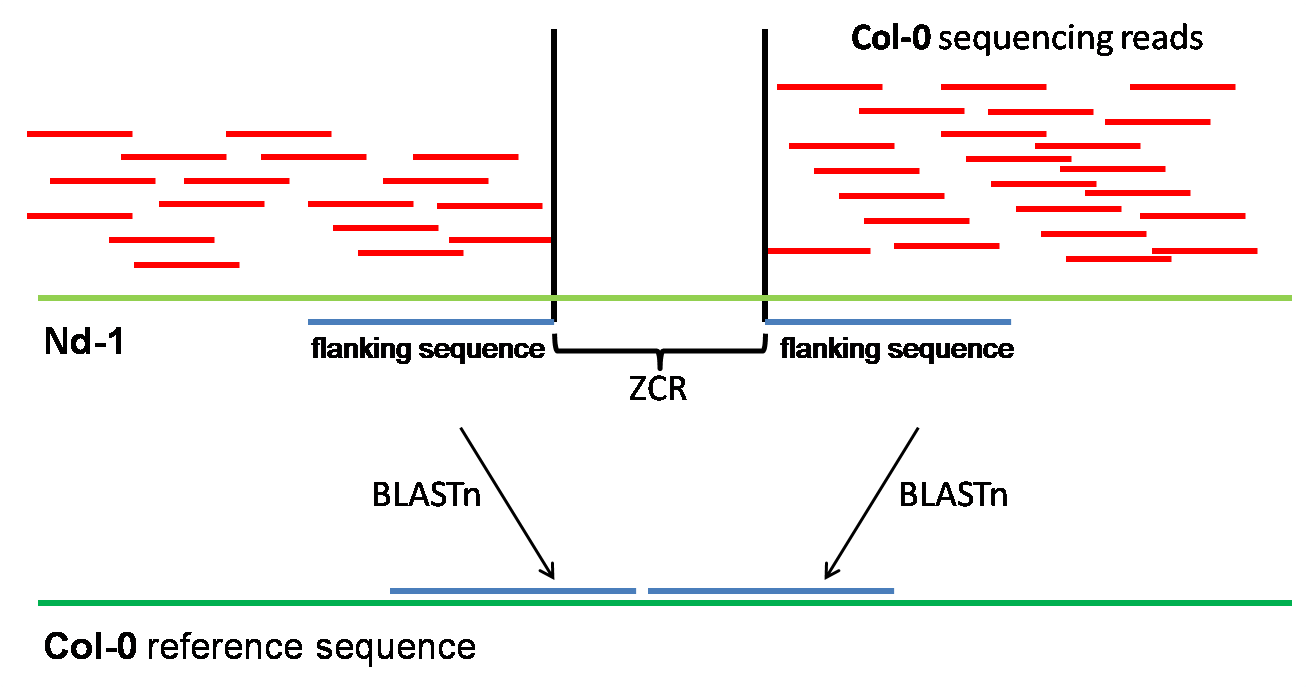

Supplement: S1 Fig — Sequencing reads of one accession (Col-0 in the example shown) were mapped to the genome sequence of the other accession (here Nd-1). ZCRs were identified from the read coverage graph. Flanking sequences of ZCRs were subjected to BLASTn against the genome sequence of the read source accession. Adjacent BLASTn hits in correct orientation confirm the absence of the ZCR in the genome that provided the reads, and indicates PAV between the two genomes studied. (TIF) [file pone.0164321.s001.tif]

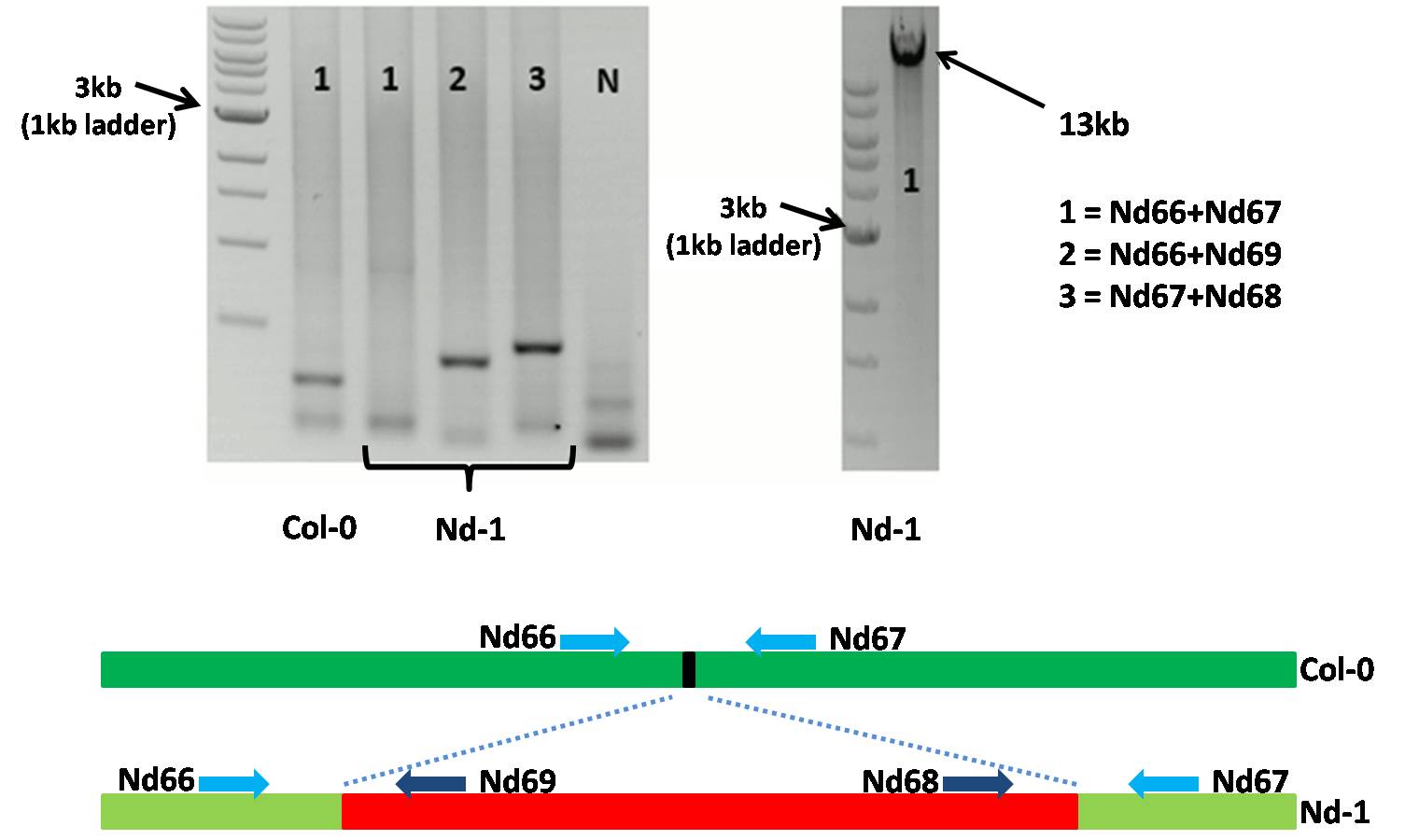

Supplement: S2 Fig — The concept is visualized by using a PAV of about 13 kbp in length that is present in Nd-1 and absent from Col-0 as an example. This figure shows the primer positions used for experimental validation (bottom). Outer primers (Nd66 and Nd67) were used for standard PCR on genomic DNA of Col-0 and Nd-1 (gel picture of amplicons, top left) and for long range PCR on genomic DNA of Nd-1 (top right). Inner primers were used for amplicon generation in standard PCR with genomic DNA of Nd-1. (JPG) [file pone.0164321.s002.jpg]

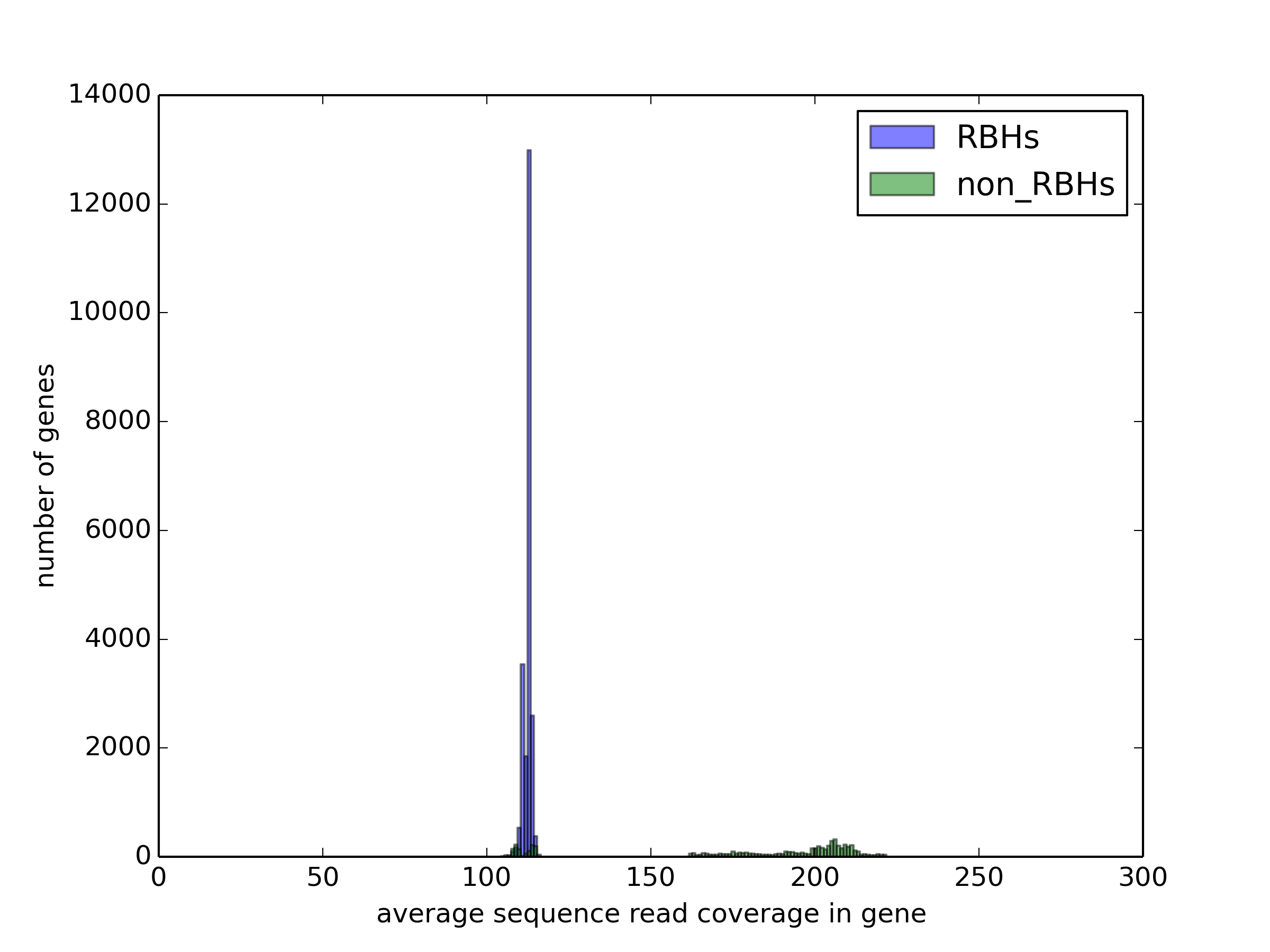

Supplement: S3 Fig — Nd-1 sequencing reads were mapped to the assembly. The average coverage within predicted genes is 112x +/- 1.1x. The average coverage inside of RBHs (blue) and inside of non-RBHs (green) is shown. (PNG) [file pone.0164321.s003.png]

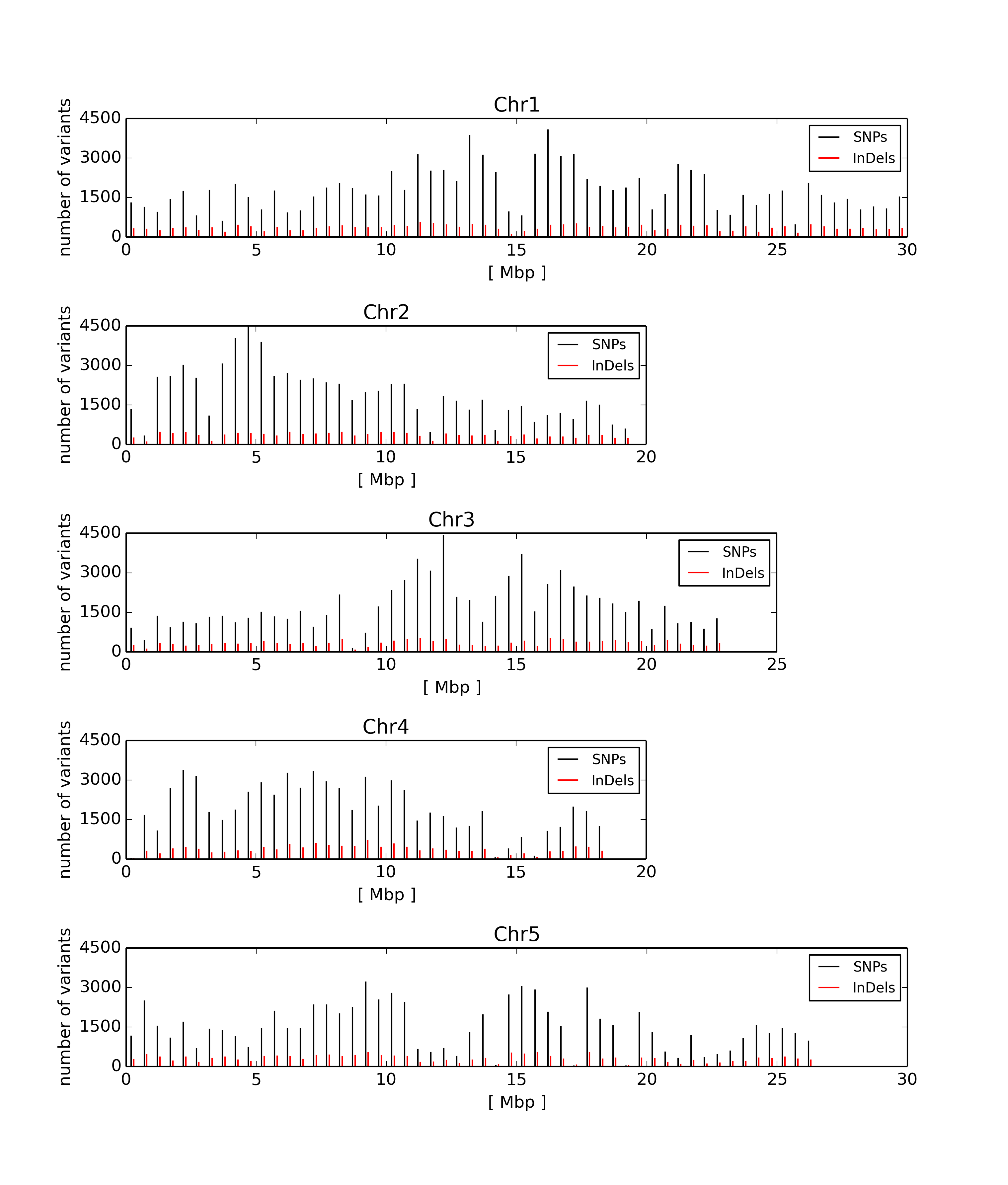

Supplement: S4 Fig — Numbers of SNPs (black) and InDels (red) in a given interval on the chromosomes are shown. Both variant types were identified using GATK and CLC genomics workbench as described in the method section. The overlap of both tools was considered as the best choice. (PNG) [file pone.0164321.s004.png]

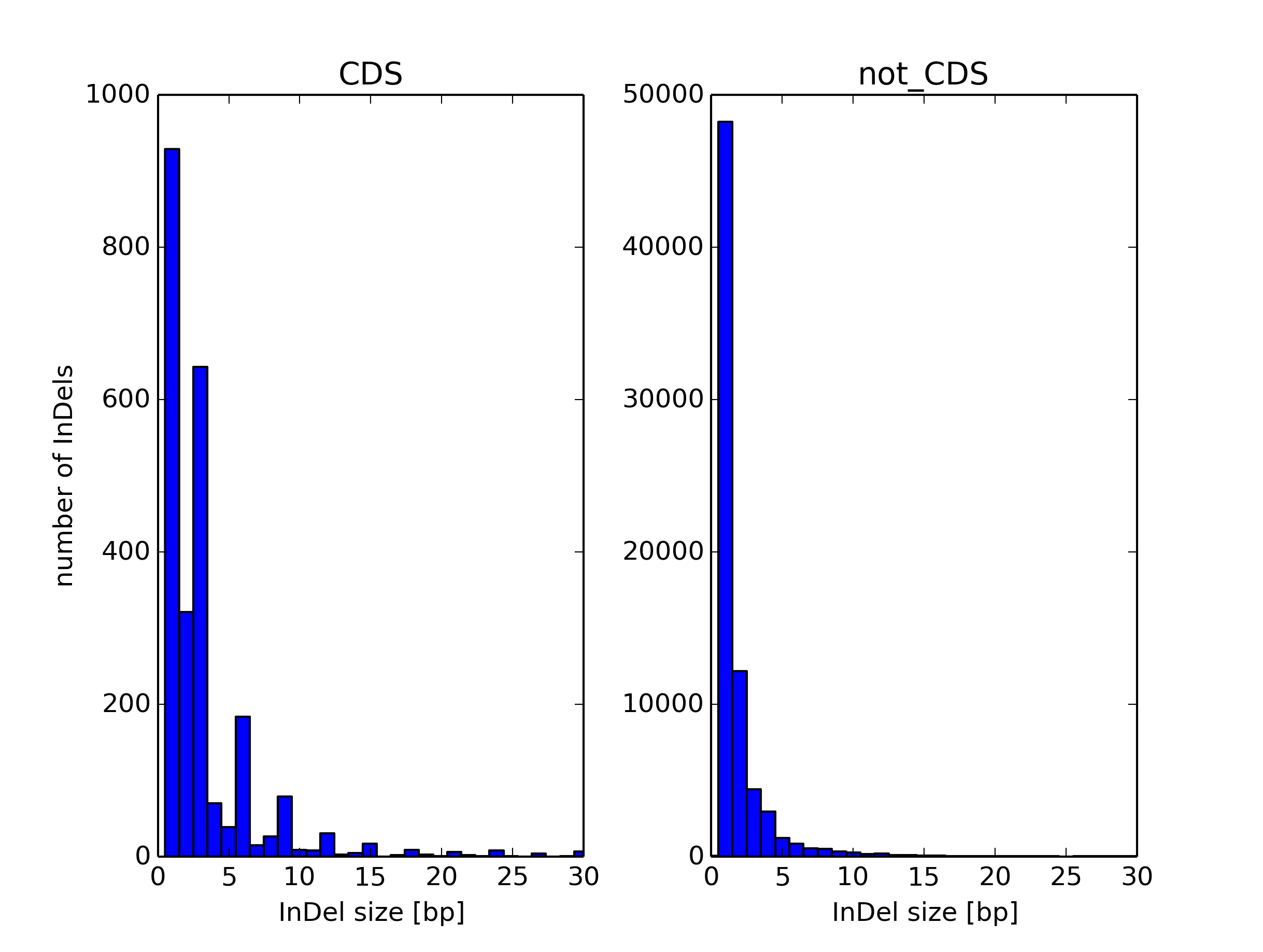

Supplement: S5 Fig — Most frequent InDel sizes differ between coding and non-coding regions. Multiple of three are much more common in coding sequences. (PNG) [file pone.0164321.s005.png]

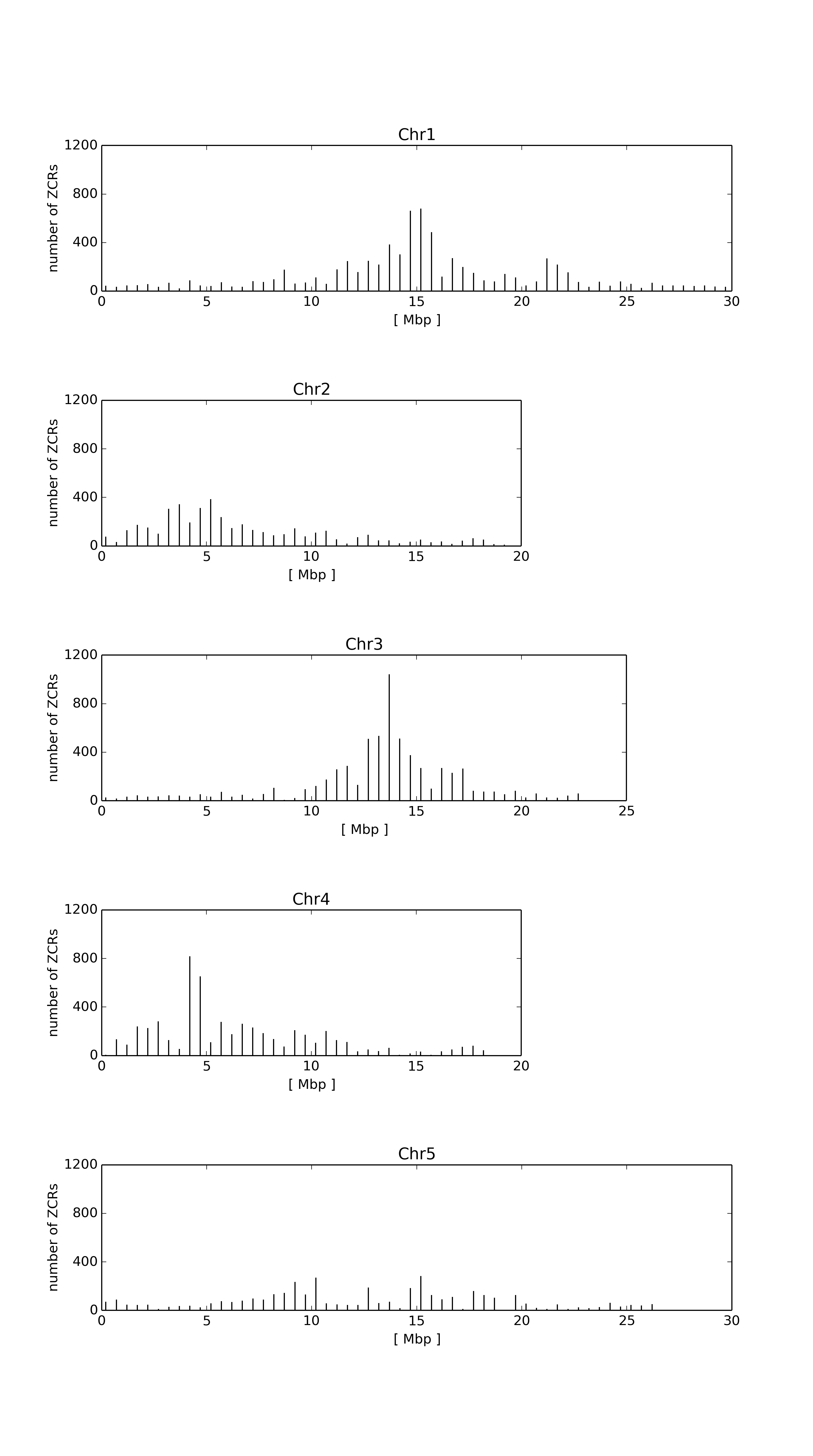

Supplement: S6 Fig — ZCRs identified via mapping of Nd-1 reads to the Col-0 reference sequence are shown. (PNG) [file pone.0164321.s006.png]

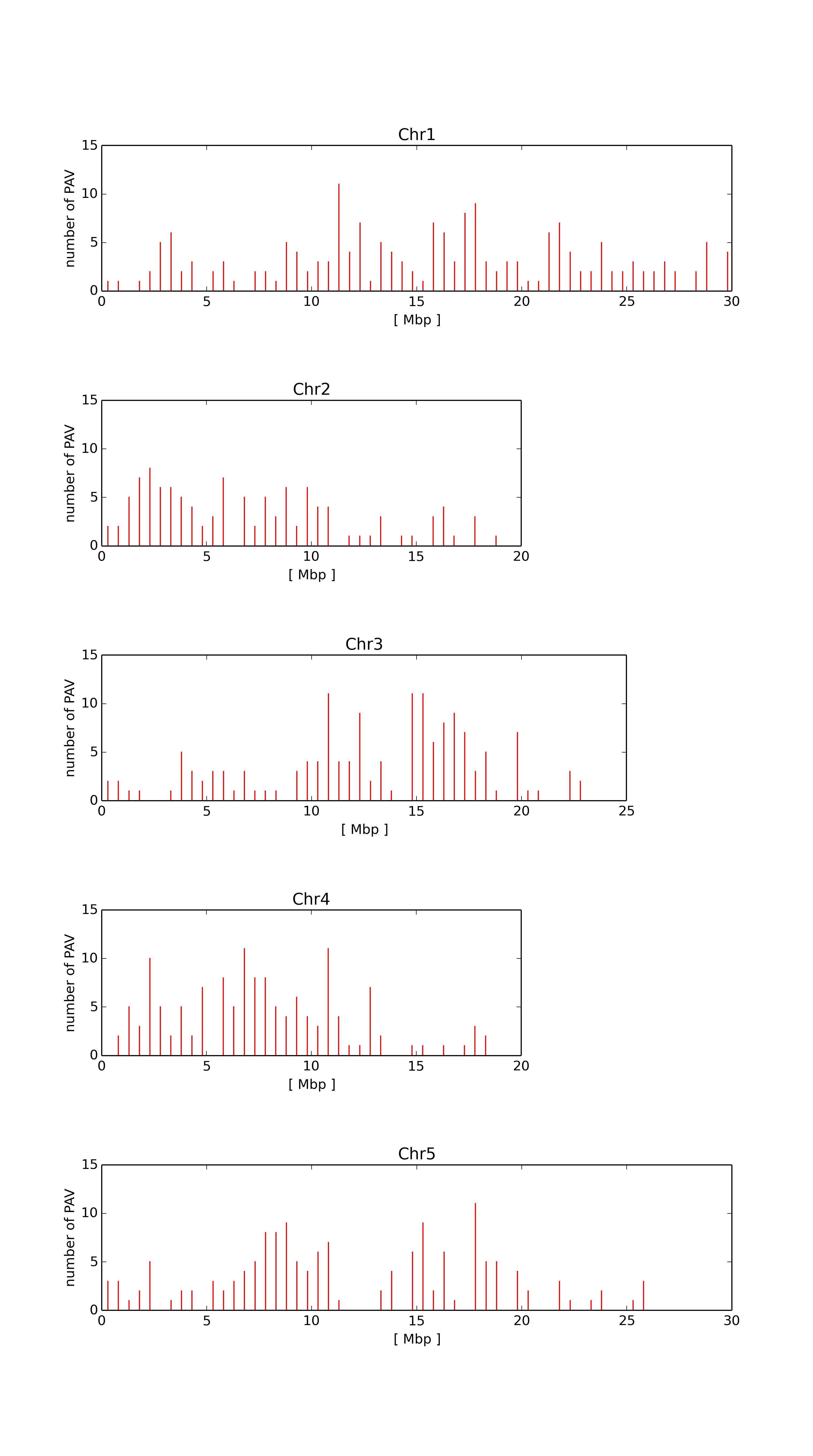

Supplement: S7 Fig — Only ZCRs with expected PAV as cause of the missing read coverage are shown. (PNG) [file pone.0164321.s007.png]
