# Supplementary material for: A De Novo Genome Sequence Assembly of the Arabidopsis thaliana Accession Niederzenz-1 Displays Presence/Absence Variation and Strong Synteny
Source: PLoS One. 2016 Oct 6;11(10):e0164321. doi: 10.1371/journal.pone.0164321 (PMC5053417; doi:10.1371/journal.pone.0164321)
Supplement: S3 File — This file was constructed by SnpEff while processing the detected variants as a summary. (HTML) [file pone.0164321.s010.html]

### SnpEff: Variant analysis

|  |
| --- |
| **Contents** Summary   Variant rate by chromosome  Variants by type   Number of variants by impact    Number of variants by functional class    Number of variants by effect   Quality histogram  InDel length histogram  Base variant table  Transition vs transversions (ts/tv)   Allele frequency    Allele Count    Codon change table    Amino acid change table    Chromosome variants plots    Details by gene |


---


**Summary**

|  |  |
| --- | --- |
| **Genome** | athalianaTair10 |
| **Date** | xxx |
| **SnpEff version** | ``` SnpEff 4.1f (build 2015-05-12), by Pablo Cingolani ``` |
| **Command line arguments** | ``` SnpEff  athalianaTair10 small_variants.vcf ``` |
| **Warnings** | 12,683 |
| **Errors** | 28 |
| **Number of lines (input file)** | 485,887 |
| **Number of variants (before filter)** | 486,515 |
| **Number of not variants  (i.e. reference equals alternative)** | 0 |
| **Number of variants processed   (i.e. after filter and non-variants)** | 486,515 |
| **Number of known variants  (i.e. non-empty ID)** | 0 ( 0% ) |
| **Number of multi-allelic VCF entries  (i.e. more than two alleles)** | 626 |
| **Number of effects** | 3,868,926 |
| **Genome total length** | 119,667,750 |
| **Genome effective length** | 119,146,348 |
| **Variant rate** | 1 variant every 244 bases |


---


 **Variants rate details** 

| Chromosome | Length | Variants | Variants rate |
| --- | --- | --- | --- |
| 1 | 30,427,671 | 128,436 | 236 |
| 2 | 19,698,289 | 86,309 | 228 |
| 3 | 23,459,830 | 94,376 | 248 |
| 4 | 18,585,056 | 83,031 | 223 |
| 5 | 26,975,502 | 94,335 | 285 |
| Total | 119,146,348 | 486,515 | 244 |


---


 **Number variantss by type**

| **Type** | **Total** |
| --- | --- |
| **SNP** | 410,093 |
| **MNP** | 0 |
| **INS** | 36,949 |
| **DEL** | 39,473 |
| **MIXED** | 0 |
| **INTERVAL** | 0 |
| **Total** | 486,515 |
| --- | --- |


---


 **Number of effects by impact** 

| **Type (alphabetical order)** |  | Count | Percent |
| --- | --- | --- | --- |
| **HIGH** |  | 5,237 | 0.135% |
| **LOW** |  | 133,414 | 3.448% |
| **MODERATE** |  | 92,802 | 2.399% |
| **MODIFIER** |  | 3,637,473 | 94.018% |


---


 **Number of effects by functional class** 

| **Type (alphabetical order)** |  | Count | Percent |
| --- | --- | --- | --- |
| **MISSENSE** |  | 90,724 | 44.025% |
| **NONSENSE** |  | 752 | 0.365% |
| **SILENT** |  | 114,600 | 55.611% |

  

Missense / Silent ratio: 0.7917 |


---


 **Number of effects by type and region** 

| Type | Region |
| --- | --- |
| | **Type (alphabetical order)** |  | Count | Percent | | --- | --- | --- | --- | |  |  | 28 | 0.001% | | **3\_prime\_UTR\_variant** |  | 24,877 | 0.643% | | **5\_prime\_UTR\_premature\_start\_codon\_gain\_variant** |  | 1,362 | 0.035% | | **5\_prime\_UTR\_variant** |  | 15,720 | 0.406% | | **disruptive\_inframe\_deletion** |  | 890 | 0.023% | | **disruptive\_inframe\_deletion+splice\_region\_variant** |  | 12 | 0% | | **disruptive\_inframe\_insertion** |  | 644 | 0.017% | | **disruptive\_inframe\_insertion+splice\_region\_variant** |  | 10 | 0% | | **downstream\_gene\_variant** |  | 1,510,970 | 39.054% | | **exon\_loss\_variant+splice\_acceptor\_variant+splice\_region\_variant+splice\_region\_variant+splice\_region\_variant+intron\_variant** |  | 1 | 0% | | **frameshift\_variant** |  | 2,837 | 0.073% | | **frameshift\_variant+splice\_acceptor\_variant+splice\_region\_variant+intron\_variant** |  | 2 | 0% | | **frameshift\_variant+splice\_acceptor\_variant+splice\_region\_variant+splice\_region\_variant+intron\_variant** |  | 6 | 0% | | **frameshift\_variant+splice\_donor\_variant+splice\_region\_variant+intron\_variant** |  | 8 | 0% | | **frameshift\_variant+splice\_donor\_variant+splice\_region\_variant+splice\_region\_variant+intron\_variant** |  | 2 | 0% | | **frameshift\_variant+splice\_region\_variant** |  | 45 | 0.001% | | **frameshift\_variant+start\_lost** |  | 184 | 0.005% | | **frameshift\_variant+start\_lost+splice\_region\_variant** |  | 2 | 0% | | **frameshift\_variant+stop\_gained** |  | 100 | 0.003% | | **frameshift\_variant+stop\_gained+splice\_region\_variant** |  | 2 | 0% | | **frameshift\_variant+stop\_lost** |  | 22 | 0.001% | | **frameshift\_variant+stop\_lost+splice\_acceptor\_variant+splice\_region\_variant+splice\_region\_variant+intron\_variant** |  | 1 | 0% | | **frameshift\_variant+stop\_lost+splice\_region\_variant** |  | 37 | 0.001% | | **frameshift\_variant+stop\_lost+splice\_region\_variant+splice\_region\_variant** |  | 1 | 0% | | **inframe\_deletion** |  | 416 | 0.011% | | **inframe\_insertion** |  | 514 | 0.013% | | **inframe\_insertion+splice\_region\_variant** |  | 6 | 0% | | **initiator\_codon\_variant** |  | 26 | 0.001% | | **intergenic\_region** |  | 242,458 | 6.267% | | **intragenic\_variant** |  | 33 | 0.001% | | **intron\_variant** |  | 201,791 | 5.216% | | **missense\_variant** |  | 89,064 | 2.302% | | **missense\_variant+splice\_region\_variant** |  | 1,246 | 0.032% | | **non\_coding\_exon\_variant** |  | 45,444 | 1.175% | | **splice\_acceptor\_variant+3\_prime\_UTR\_variant+intron\_variant** |  | 2 | 0% | | **splice\_acceptor\_variant+5\_prime\_UTR\_variant+intron\_variant** |  | 2 | 0% | | **splice\_acceptor\_variant+inframe\_deletion+splice\_region\_variant+splice\_region\_variant+intron\_variant** |  | 4 | 0% | | **splice\_acceptor\_variant+intron\_variant** |  | 390 | 0.01% | | **splice\_acceptor\_variant+splice\_region\_variant+3\_prime\_UTR\_variant+intron\_variant** |  | 1 | 0% | | **splice\_acceptor\_variant+splice\_region\_variant+5\_prime\_UTR\_variant+intron\_variant** |  | 1 | 0% | | **splice\_acceptor\_variant+splice\_region\_variant+intron\_variant** |  | 23 | 0.001% | | **splice\_acceptor\_variant+splice\_region\_variant+splice\_region\_variant+intron\_variant+non\_coding\_exon\_variant** |  | 1 | 0% | | **splice\_donor\_variant+intron\_variant** |  | 340 | 0.009% | | **splice\_donor\_variant+splice\_region\_variant+3\_prime\_UTR\_variant+intron\_variant** |  | 2 | 0% | | **splice\_donor\_variant+splice\_region\_variant+5\_prime\_UTR\_variant+intron\_variant** |  | 1 | 0% | | **splice\_donor\_variant+splice\_region\_variant+intron\_variant** |  | 38 | 0.001% | | **splice\_region\_variant** |  | 501 | 0.013% | | **splice\_region\_variant+downstream\_gene\_variant** |  | 1 | 0% | | **splice\_region\_variant+intron\_variant** |  | 16,759 | 0.433% | | **splice\_region\_variant+non\_coding\_exon\_variant** |  | 165 | 0.004% | | **splice\_region\_variant+stop\_retained\_variant** |  | 120 | 0.003% | | **splice\_region\_variant+synonymous\_variant** |  | 1,696 | 0.044% | | **start\_lost** |  | 158 | 0.004% | | **start\_lost+disruptive\_inframe\_insertion** |  | 2 | 0% | | **start\_lost+inframe\_deletion** |  | 12 | 0% | | **start\_lost+inframe\_insertion** |  | 10 | 0% | | **stop\_gained** |  | 742 | 0.019% | | **stop\_gained+inframe\_insertion** |  | 6 | 0% | | **stop\_gained+splice\_region\_variant** |  | 10 | 0% | | **stop\_lost** |  | 55 | 0.001% | | **stop\_lost+inframe\_deletion** |  | 2 | 0% | | **stop\_lost+inframe\_deletion+splice\_region\_variant** |  | 10 | 0% | | **stop\_lost+splice\_region\_variant** |  | 173 | 0.004% | | **stop\_lost+splice\_region\_variant+splice\_region\_variant** |  | 2 | 0% | | **stop\_retained\_variant** |  | 78 | 0.002% | | **synonymous\_variant** |  | 112,706 | 2.913% | | **transcript** |  | 9,787 | 0.253% | | **upstream\_gene\_variant** |  | 1,586,365 | 41.003% | | | **Type (alphabetical order)** |  | Count | Percent | | --- | --- | --- | --- | | **DOWNSTREAM** |  | 1,510,970 | 39.054% | | **EXON** |  | 255,280 | 6.598% | | **INTERGENIC** |  | 242,458 | 6.267% | | **INTRON** |  | 201,791 | 5.216% | | **NONE** |  | 9,848 | 0.255% | | **SPLICE\_SITE\_ACCEPTOR** |  | 424 | 0.011% | | **SPLICE\_SITE\_DONOR** |  | 381 | 0.01% | | **SPLICE\_SITE\_REGION** |  | 19,242 | 0.497% | | **TRANSCRIPT** |  | 208 | 0.005% | | **UPSTREAM** |  | 1,586,365 | 41.003% | | **UTR\_3\_PRIME** |  | 24,877 | 0.643% | | **UTR\_5\_PRIME** |  | 17,082 | 0.442% | |


---


 **Quality:**

```
|  |  |
| --- | --- |
| Min | 13 |
| Max | 314,372 |
| Mean | 2,544.324 |
| Median | 2,557 |
| Standard deviation | 1,420.379 |
| Values | 13,14,15,16,18,20,22,23,24,30,31,32,33,34,35,36,37,38,39,40,41,42,43,44,45,46,47,48,49,50,51,52,53,54,55,56,57,58,59,60,61,62,63,64,65,66,67,68,69,70,71,72,73,74,75,76,77,78,79,80,81,82,83,84,85,86,87,88,89,90,91,92,93,94,95,96,97,98,99,100,101,102,103,104,105,106,107,108,109,110,111,112,113,114,115,116,117,118,119,120,121,122,123,124,125,126,127,128,129,130,131,132,133,134,135,136,137,138,139,140,141,142,143,144,145,146,147,148,149,150,151,152,153,154,155,156,157,158,159,160,161,162,163,164,165,166,167,168,169,170,171,172,173,174,175,176,177,178,179,180,181,182,183,184,185,186,187,188,189,190,191,192,193,194,195,196,197,198,199,200,201,202,203,204,205,206,207,208,209,210,211,212,213,214,215,216,217,218,219,220,221,222,223,224,225,226,227,228,229,230,231,232,233,234,235,236,237,238,239,240,241,242,243,244,245,246,247,248,249,250,251,252,253,254,255,256,257,258,259,260,261,262,263,264,265,266,267,268,269,270,271,272,273,274,275,276,277,278,279,280,281,282,283,284,285,286,287,288,289,290,291,292,293,294,295,296,297,298,299,300,301,302,303,304,305,306,307,308,309,310,311,312,313,314,315,316,317,318,319,320,321,322,323,324,325,326,327,328,329,330,331,332,333,334,335,336,337,338,339,340,341,342,343,344,345,346,347,348,349,350,351,352,353,354,355,356,357,358,359,360,361,362,363,364,365,366,367,368,369,370,371,372,373,374,375,376,377,378,379,380,381,382,383,384,385,386,387,388,389,390,391,392,393,394,395,396,397,398,399,400,401,402,403,404,405,406,407,408,409,410,411,412,413,414,415,416,417,418,419,420,421,422,423,424,425,426,427,428,429,430,431,432,433,434,435,436,437,438,439,440,441,442,443,444,445,446,447,448,449,450,451,452,453,454,455,456,457,458,459,460,461,462,463,464,465,466,467,468,469,470,471,472,473,474,475,476,477,478,479,480,481,482,483,484,485,486,487,488,489,490,491,492,493,494,495,496,497,498,499,500,501,502,503,504,505,506,507,508,509,510,511,512,513,514,515,516,517,518,519,520,521,522,523,524,525,526,527,528,529,530,531,532,533,534,535,536,537,538,539,540,541,542,543,544,545,546,547,548,549,550,551,552,553,554,555,556,557,558,559,560,561,562,563,564,565,566,567,568,569,570,571,572,573,574,575,576,577,578,579,580,581,582,583,584,585,586,587,588,589,590,591,592,593,594,595,596,597,598,599,600,601,602,603,604,605,606,607,608,609,610,611,612,613,614,615,616,617,618,619,620,621,622,623,624,625,626,627,628,629,630,631,632,633,634,635,636,637,638,639,640,641,642,643,644,645,646,647,648,649,650,651,652,653,654,655,656,657,658,659,660,661,662,663,664,665,666,667,668,669,670,671,672,673,674,675,676,677,678,679,680,681,682,683,684,685,686,687,688,689,690,691,692,693,694,695,696,697,698,699,700,701,702,703,704,705,706,707,708,709,710,711,712,713,714,715,716,717,718,719,720,721,722,723,724,725,726,727,728,729,730,731,732,733,734,735,736,737,738,739,740,741,742,743,744,745,746,747,748,749,750,751,752,753,754,755,756,757,758,759,760,761,762,763,764,765,766,767,768,769,770,771,772,773,774,775,776,777,778,779,780,781,782,783,784,785,786,787,788,789,790,791,792,793,794,795,796,797,798,799,800,801,802,803,804,805,806,807,808,809,810,811,812,813,814,815,816,817,818,819,820,821,822,823,824,825,826,827,828,829,830,831,832,833,834,835,836,837,838,839,840,841,842,843,844,845,846,847,848,849,850,851,852,853,854,855,856,857,858,859,860,861,862,863,864,865,866,867,868,869,870,871,872,873,874,875,876,877,878,879,880,881,882,883,884,885,886,887,888,889,890,891,892,893,894,895,896,897,898,899,900,901,902,903,904,905,906,907,908,909,910,911,912,913,914,915,916,917,918,919,920,921,922,923,924,925,926,927,928,929,930,931,932,933,934,935,936,937,938,939,940,941,942,943,944,945,946,947,948,949,950,951,952,953,954,955,956,957,958,959,960,961,962,963,964,965,966,967,968,969,970,971,972,973,974,975,976,977,978,979,980,981,982,983,984,985,986,987,988,989,990,991,992,993,994,995,996,997,998,999,1000,1001,1002,1003,1004,1005,1006,1007,1008,1009,1010,1011,1012,1013,1014,1015,1016,1017,1018,1019,1020,1021,1022,1023,1024,1025,1026,1027,1028,1029,1030,1031,1032,1033,1034,1035,1036,1037,1038,1039,1040,1041,1042,1043,1044,1045,1046,1047,1048,1049,1050,1051,1052,1053,1054,1055,1056,1057,1058,1059,1060,1061,1062,1063,1064,1065,1066,1067,1068,1069,1070,1071,1072,1073,1074,1075,1076,1077,1078,1079,1080,1081,1082,1083,1084,1085,1086,1087,1088,1089,1090,1091,1092,1093,1094,1095,1096,1097,1098,1099,1100,1101,1102,1103,1104,1105,1106,1107,1108,1109,1110,1111,1112,1113,1114,1115,1116,1117,1118,1119,1120,1121,1122,1123,1124,1125,1126,1127,1128,1129,1130,1131,1132,1133,1134,1135,1136,1137,1138,1139,1140,1141,1142,1143,1144,1145,1146,1147,1148,1149,1150,1151,1152,1153,1154,1155,1156,1157,1158,1159,1160,1161,1162,1163,1164,1165,1166,1167,1168,1169,1170,1171,1172,1173,1174,1175,1176,1177,1178,1179,1180,1181,1182,1183,1184,1185,1186,1187,1188,1189,1190,1191,1192,1193,1194,1195,1196,1197,1198,1199,1200,1201,1202,1203,1204,1205,1206,1207,1208,1209,1210,1211,1212,1213,1214,1215,1216,1217,1218,1219,1220,1221,1222,1223,1224,1225,1226,1227,1228,1229,1230,1231,1232,1233,1234,1235,1236,1237,1238,1239,1240,1241,1242,1243,1244,1245,1246,1247,1248,1249,1250,1251,1252,1253,1254,1255,1256,1257,1258,1259,1260,1261,1262,1263,1264,1265,1266,1267,1268,1269,1270,1271,1272,1273,1274,1275,1276,1277,1278,1279,1280,1281,1282,1283,1284,1285,1286,1287,1288,1289,1290,1291,1292,1293,1294,1295,1296,1297,1298,1299,1300,1301,1302,1303,1304,1305,1306,1307,1308,1309,1310,1311,1312,1313,1314,1315,1316,1317,1318,1319,1320,1321,1322,1323,1324,1325,1326,1327,1328,1329,1330,1331,1332,1333,1334,1335,1336,1337,1338,1339,1340,1341,1342,1343,1344,1345,1346,1347,1348,1349,1350,1351,1352,1353,1354,1355,1356,1357,1358,1359,1360,1361,1362,1363,1364,1365,1366,1367,1368,1369,1370,1371,1372,1373,1374,1375,1376,1377,1378,1379,1380,1381,1382,1383,1384,1385,1386,1387,1388,1389,1390,1391,1392,1393,1394,1395,1396,1397,1398,1399,1400,1401,1402,1403,1404,1405,1406,1407,1408,1409,1410,1411,1412,1413,1414,1415,1416,1417,1418,1419,1420,1421,1422,1423,1424,1425,1426,1427,1428,1429,1430,1431,1432,1433,1434,1435,1436,1437,1438,1439,1440,1441,1442,1443,1444,1445,1446,1447,1448,1449,1450,1451,1452,1453,1454,1455,1456,1457,1458,1459,1460,1461,1462,1463,1464,1465,1466,1467,1468,1469,1470,1471,1472,1473,1474,1475,1476,1477,1478,1479,1480,1481,1482,1483,1484,1485,1486,1487,1488,1489,1490,1491,1492,1493,1494,1495,1496,1497,1498,1499,1500,1501,1502,1503,1504,1505,1506,1507,1508,1509,1510,1511,1512,1513,1514,1515,1516,1517,1518,1519,1520,1521,1522,1523,1524,1525,1526,1527,1528,1529,1530,1531,1532,1533,1534,1535,1536,1537,1538,1539,1540,1541,1542,1543,1544,1545,1546,1547,1548,1549,1550,1551,1552,1553,1554,1555,1556,1557,1558,1559,1560,1561,1562,1563,1564,1565,1566,1567,1568,1569,1570,1571,1572,1573,1574,1575,1576,1577,1578,1579,1580,1581,1582,1583,1584,1585,1586,1587,1588,1589,1590,1591,1592,1593,1594,1595,1596,1597,1598,1599,1600,1601,1602,1603,1604,1605,1606,1607,1608,1609,1610,1611,1612,1613,1614,1615,1616,1617,1618,1619,1620,1621,1622,1623,1624,1625,1626,1627,1628,1629,1630,1631,1632,1633,1634,1635,1636,1637,1638,1639,1640,1641,1642,1643,1644,1645,1646,1647,1648,1649,1650,1651,1652,1653,1654,1655,1656,1657,1658,1659,1660,1661,1662,1663,1664,1665,1666,1667,1668,1669,1670,1671,1672,1673,1674,1675,1676,1677,1678,1679,1680,1681,1682,1683,1684,1685,1686,1687,1688,1689,1690,1691,1692,1693,1694,1695,1696,1697,1698,1699,1700,1701,1702,1703,1704,1705,1706,1707,1708,1709,1710,1711,1712,1713,1714,1715,1716,1717,1718,1719,1720,1721,1722,1723,1724,1725,1726,1727,1728,1729,1730,1731,1732,1733,1734,1735,1736,1737,1738,1739,1740,1741,1742,1743,1744,1745,1746,1747,1748,1749,1750,1751,1752,1753,1754,1755,1756,1757,1758,1759,1760,1761,1762,1763,1764,1765,1766,1767,1768,1769,1770,1771,1772,1773,1774,1775,1776,1777,1778,1779,1780,1781,1782,1783,1784,1785,1786,1787,1788,1789,1790,1791,1792,1793,1794,1795,1796,1797,1798,1799,1800,1801,1802,1803,1804,1805,1806,1807,1808,1809,1810,1811,1812,1813,1814,1815,1816,1817,1818,1819,1820,1821,1822,1823,1824,1825,1826,1827,1828,1829,1830,1831,1832,1833,1834,1835,1836,1837,1838,1839,1840,1841,1842,1843,1844,1845,1846,1847,1848,1849,1850,1851,1852,1853,1854,1855,1856,1857,1858,1859,1860,1861,1862,1863,1864,1865,1866,1867,1868,1869,1870,1871,1872,1873,1874,1875,1876,1877,1878,1879,1880,1881,1882,1883,1884,1885,1886,1887,1888,1889,1890,1891,1892,1893,1894,1895,1896,1897,1898,1899,1900,1901,1902,1903,1904,1905,1906,1907,1908,1909,1910,1911,1912,1913,1914,1915,1916,1917,1918,1919,1920,1921,1922,1923,1924,1925,1926,1927,1928,1929,1930,1931,1932,1933,1934,1935,1936,1937,1938,1939,1940,1941,1942,1943,1944,1945,1946,1947,1948,1949,1950,1951,1952,1953,1954,1955,1956,1957,1958,1959,1960,1961,1962,1963,1964,1965,1966,1967,1968,1969,1970,1971,1972,1973,1974,1975,1976,1977,1978,1979,1980,1981,1982,1983,1984,1985,1986,1987,1988,1989,1990,1991,1992,1993,1994,1995,1996,1997,1998,1999,2000,2001,2002,2003,2004,2005,2006,2007,2008,2009,2010,2011,2012,2013,2014,2015,2016,2017,2018,2019,2020,2021,2022,2023,2024,2025,2026,2027,2028,2029,2030,2031,2032,2033,2034,2035,2036,2037,2038,2039,2040,2041,2042,2043,2044,2045,2046,2047,2048,2049,2050,2051,2052,2053,2054,2055,2056,2057,2058,2059,2060,2061,2062,2063,2064,2065,2066,2067,2068,2069,2070,2071,2072,2073,2074,2075,2076,2077,2078,2079,2080,2081,2082,2083,2084,2085,2086,2087,2088,2089,2090,2091,2092,2093,2094,2095,2096,2097,2098,2099,2100,2101,2102,2103,2104,2105,2106,2107,2108,2109,2110,2111,2112,2113,2114,2115,2116,2117,2118,2119,2120,2121,2122,2123,2124,2125,2126,2127,2128,2129,2130,2131,2132,2133,2134,2135,2136,2137,2138,2139,2140,2141,2142,2143,2144,2145,2146,2147,2148,2149,2150,2151,2152,2153,2154,2155,2156,2157,2158,2159,2160,2161,2162,2163,2164,2165,2166,2167,2168,2169,2170,2171,2172,2173,2174,2175,2176,2177,2178,2179,2180,2181,2182,2183,2184,2185,2186,2187,2188,2189,2190,2191,2192,2193,2194,2195,2196,2197,2198,2199,2200,2201,2202,2203,2204,2205,2206,2207,2208,2209,2210,2211,2212,2213,2214,2215,2216,2217,2218,2219,2220,2221,2222,2223,2224,2225,2226,2227,2228,2229,2230,2231,2232,2233,2234,2235,2236,2237,2238,2239,2240,2241,2242,2243,2244,2245,2246,2247,2248,2249,2250,2251,2252,2253,2254,2255,2256,2257,2258,2259,2260,2261,2262,2263,2264,2265,2266,2267,2268,2269,2270,2271,2272,2273,2274,2275,2276,2277,2278,2279,2280,2281,2282,2283,2284,2285,2286,2287,2288,2289,2290,2291,2292,2293,2294,2295,2296,2297,2298,2299,2300,2301,2302,2303,2304,2305,2306,2307,2308,2309,2310,2311,2312,2313,2314,2315,2316,2317,2318,2319,2320,2321,2322,2323,2324,2325,2326,2327,2328,2329,2330,2331,2332,2333,2334,2335,2336,2337,2338,2339,2340,2341,2342,2343,2344,2345,2346,2347,2348,2349,2350,2351,2352,2353,2354,2355,2356,2357,2358,2359,2360,2361,2362,2363,2364,2365,2366,2367,2368,2369,2370,2371,2372,2373,2374,2375,2376,2377,2378,2379,2380,2381,2382,2383,2384,2385,2386,2387,2388,2389,2390,2391,2392,2393,2394,2395,2396,2397,2398,2399,2400,2401,2402,2403,2404,2405,2406,2407,2408,2409,2410,2411,2412,2413,2414,2415,2416,2417,2418,2419,2420,2421,2422,2423,2424,2425,2426,2427,2428,2429,2430,2431,2432,2433,2434,2435,2436,2437,2438,2439,2440,2441,2442,2443,2444,2445,2446,2447,2448,2449,2450,2451,2452,2453,2454,2455,2456,2457,2458,2459,2460,2461,2462,2463,2464,2465,2466,2467,2468,2469,2470,2471,2472,2473,2474,2475,2476,2477,2478,2479,2480,2481,2482,2483,2484,2485,2486,2487,2488,2489,2490,2491,2492,2493,2494,2495,2496,2497,2498,2499,2500,2501,2502,2503,2504,2505,2506,2507,2508,2509,2510,2511,2512,2513,2514,2515,2516,2517,2518,2519,2520,2521,2522,2523,2524,2525,2526,2527,2528,2529,2530,2531,2532,2533,2534,2535,2536,2537,2538,2539,2540,2541,2542,2543,2544,2545,2546,2547,2548,2549,2550,2551,2552,2553,2554,2555,2556,2557,2558,2559,2560,2561,2562,2563,2564,2565,2566,2567,2568,2569,2570,2571,2572,2573,2574,2575,2576,2577,2578,2579,2580,2581,2582,2583,2584,2585,2586,2587,2588,2589,2590,2591,2592,2593,2594,2595,2596,2597,2598,2599,2600,2601,2602,2603,2604,2605,2606,2607,2608,2609,2610,2611,2612,2613,2614,2615,2616,2617,2618,2619,2620,2621,2622,2623,2624,2625,2626,2627,2628,2629,2630,2631,2632,2633,2634,2635,2636,2637,2638,2639,2640,2641,2642,2643,2644,2645,2646,2647,2648,2649,2650,2651,2652,2653,2654,2655,2656,2657,2658,2659,2660,2661,2662,2663,2664,2665,2666,2667,2668,2669,2670,2671,2672,2673,2674,2675,2676,2677,2678,2679,2680,2681,2682,2683,2684,2685,2686,2687,2688,2689,2690,2691,2692,2693,2694,2695,2696,2697,2698,2699,2700,2701,2702,2703,2704,2705,2706,2707,2708,2709,2710,2711,2712,2713,2714,2715,2716,2717,2718,2719,2720,2721,2722,2723,2724,2725,2726,2727,2728,2729,2730,2731,2732,2733,2734,2735,2736,2737,2738,2739,2740,2741,2742,2743,2744,2745,2746,2747,2748,2749,2750,2751,2752,2753,2754,2755,2756,2757,2758,2759,2760,2761,2762,2763,2764,2765,2766,2767,2768,2769,2770,2771,2772,2773,2774,2775,2776,2777,2778,2779,2780,2781,2782,2783,2784,2785,2786,2787,2788,2789,2790,2791,2792,2793,2794,2795,2796,2797,2798,2799,2800,2801,2802,2803,2804,2805,2806,2807,2808,2809,2810,2811,2812,2813,2814,2815,2816,2817,2818,2819,2820,2821,2822,2823,2824,2825,2826,2827,2828,2829,2830,2831,2832,2833,2834,2835,2836,2837,2838,2839,2840,2841,2842,2843,2844,2845,2846,2847,2848,2849,2850,2851,2852,2853,2854,2855,2856,2857,2858,2859,2860,2861,2862,2863,2864,2865,2866,2867,2868,2869,2870,2871,2872,2873,2874,2875,2876,2877,2878,2879,2880,2881,2882,2883,2884,2885,2886,2887,2888,2889,2890,2891,2892,2893,2894,2895,2896,2897,2898,2899,2900,2901,2902,2903,2904,2905,2906,2907,2908,2909,2910,2911,2912,2913,2914,2915,2916,2917,2918,2919,2920,2921,2922,2923,2924,2925,2926,2927,2928,2929,2930,2931,2932,2933,2934,2935,2936,2937,2938,2939,2940,2941,2942,2943,2944,2945,2946,2947,2948,2949,2950,2951,2952,2953,2954,2955,2956,2957,2958,2959,2960,2961,2962,2963,2964,2965,2966,2967,2968,2969,2970,2971,2972,2973,2974,2975,2976,2977,2978,2979,2980,2981,2982,2983,2984,2985,2986,2987,2988,2989,2990,2991,2992,2993,2994,2995,2996,2997,2998,2999,3000,3001,3002,3003,3004,3005,3006,3007,3008,3009,3010,3011,3012,3013,3014,3015,3016,3017,3018,3019,3020,3021,3022,3023,3024,3025,3026,3027,3028,3029,3030,3031,3032,3033,3034,3035,3036,3037,3038,3039,3040,3041,3042,3043,3044,3045,3046,3047,3048,3049,3050,3051,3052,3053,3054,3055,3056,3057,3058,3059,3060,3061,3062,3063,3064,3065,3066,3067,3068,3069,3070,3071,3072,3073,3074,3075,3076,3077,3078,3079,3080,3081,3082,3083,3084,3085,3086,3087,3088,3089,3090,3091,3092,3093,3094,3095,3096,3097,3098,3099,3100,3101,3102,3103,3104,3105,3106,3107,3108,3109,3110,3111,3112,3113,3114,3115,3116,3117,3118,3119,3120,3121,3122,3123,3124,3125,3126,3127,3128,3129,3130,3131,3132,3133,3134,3135,3136,3137,3138,3139,3140,3141,3142,3143,3144,3145,3146,3147,3148,3149,3150,3151,3152,3153,3154,3155,3156,3157,3158,3159,3160,3161,3162,3163,3164,3165,3166,3167,3168,3169,3170,3171,3172,3173,3174,3175,3176,3177,3178,3179,3180,3181,3182,3183,3184,3185,3186,3187,3188,3189,3190,3191,3192,3193,3194,3195,3196,3197,3198,3199,3200,3201,3202,3203,3204,3205,3206,3207,3208,3209,3210,3211,3212,3213,3214,3215,3216,3217,3218,3219,3220,3221,3222,3223,3224,3225,3226,3227,3228,3229,3230,3231,3232,3233,3234,3235,3236,3237,3238,3239,3240,3241,3242,3243,3244,3245,3246,3247,3248,3249,3250,3251,3252,3253,3254,3255,3256,3257,3258,3259,3260,3261,3262,3263,3264,3265,3266,3267,3268,3269,3270,3271,3272,3273,3274,3275,3276,3277,3278,3279,3280,3281,3282,3283,3284,3285,3286,3287,3288,3289,3290,3291,3292,3293,3294,3295,3296,3297,3298,3299,3300,3301,3302,3303,3304,3305,3306,3307,3308,3309,3310,3311,3312,3313,3314,3315,3316,3317,3318,3319,3320,3321,3322,3323,3324,3325,3326,3327,3328,3329,3330,3331,3332,3333,3334,3335,3336,3337,3338,3339,3340,3341,3342,3343,3344,3345,3346,3347,3348,3349,3350,3351,3352,3353,3354,3355,3356,3357,3358,3359,3360,3361,3362,3363,3364,3365,3366,3367,3368,3369,3370,3371,3372,3373,3374,3375,3376,3377,3378,3379,3380,3381,3382,3383,3384,3385,3386,3387,3388,3389,3390,3391,3392,3393,3394,3395,3396,3397,3398,3399,3400,3401,3402,3403,3404,3405,3406,3407,3408,3409,3410,3411,3412,3413,3414,3415,3416,3417,3418,3419,3420,3421,3422,3423,3424,3425,3426,3427,3428,3429,3430,3431,3432,3433,3434,3435,3436,3437,3438,3439,3440,3441,3442,3443,3444,3445,3446,3447,3448,3449,3450,3451,3452,3453,3454,3455,3456,3457,3458,3459,3460,3461,3462,3463,3464,3465,3466,3467,3468,3469,3470,3471,3472,3473,3474,3475,3476,3477,3478,3479,3480,3481,3482,3483,3484,3485,3486,3487,3488,3489,3490,3491,3492,3493,3494,3495,3496,3497,3498,3499,3500,3501,3502,3503,3504,3505,3506,3507,3508,3509,3510,3511,3512,3513,3514,3515,3516,3517,3518,3519,3520,3521,3522,3523,3524,3525,3526,3527,3528,3529,3530,3531,3532,3533,3534,3535,3536,3537,3538,3539,3540,3541,3542,3543,3544,3545,3546,3547,3548,3549,3550,3551,3552,3553,3554,3555,3556,3557,3558,3559,3560,3561,3562,3563,3564,3565,3566,3567,3568,3569,3570,3571,3572,3573,3574,3575,3576,3577,3578,3579,3580,3581,3582,3583,3584,3585,3586,3587,3588,3589,3590,3591,3592,3593,3594,3595,3596,3597,3598,3599,3600,3601,3602,3603,3604,3605,3606,3607,3608,3609,3610,3611,3612,3613,3614,3615,3616,3617,3618,3619,3620,3621,3622,3623,3624,3625,3626,3627,3628,3629,3630,3631,3632,3633,3634,3635,3636,3637,3638,3639,3640,3641,3642,3643,3644,3645,3646,3647,3648,3649,3650,3651,3652,3653,3654,3655,3656,3657,3658,3659,3660,3661,3662,3663,3664,3665,3666,3667,3668,3669,3670,3671,3672,3673,3674,3675,3676,3677,3678,3679,3680,3681,3682,3683,3684,3685,3686,3687,3688,3689,3690,3691,3692,3693,3694,3695,3696,3697,3698,3699,3700,3701,3702,3703,3704,3705,3706,3707,3708,3709,3710,3711,3712,3713,3714,3715,3716,3717,3718,3719,3720,3721,3722,3723,3724,3725,3726,3727,3728,3729,3730,3731,3732,3733,3734,3735,3736,3737,3738,3739,3740,3741,3742,3743,3744,3745,3746,3747,3748,3749,3750,3751,3752,3753,3754,3755,3756,3757,3758,3759,3760,3761,3762,3763,3764,3765,3766,3767,3768,3769,3770,3771,3772,3773,3774,3775,3776,3777,3778,3779,3780,3781,3782,3783,3784,3785,3786,3787,3788,3789,3790,3791,3792,3793,3794,3795,3796,3797,3798,3799,3800,3801,3802,3803,3804,3805,3806,3807,3808,3809,3810,3811,3812,3813,3814,3815,3816,3817,3818,3819,3820,3821,3822,3823,3824,3825,3826,3827,3828,3829,3830,3831,3832,3833,3834,3835,3836,3837,3838,3839,3840,3841,3842,3843,3844,3845,3846,3847,3848,3849,3850,3851,3852,3853,3854,3855,3856,3857,3858,3859,3860,3861,3862,3863,3864,3865,3866,3867,3868,3869,3870,3871,3872,3873,3874,3875,3876,3877,3878,3879,3880,3881,3882,3883,3884,3885,3886,3887,3888,3889,3890,3891,3892,3893,3894,3895,3896,3897,3898,3899,3900,3901,3902,3903,3904,3905,3906,3907,3908,3909,3910,3911,3912,3913,3914,3915,3916,3917,3918,3919,3920,3921,3922,3923,3924,3925,3926,3927,3928,3929,3930,3931,3932,3933,3934,3935,3936,3937,3938,3939,3940,3941,3942,3943,3944,3945,3946,3947,3948,3949,3950,3951,3952,3953,3954,3955,3956,3957,3958,3959,3960,3961,3962,3963,3964,3965,3966,3967,3968,3969,3970,3971,3972,3973,3974,3975,3976,3977,3978,3979,3980,3981,3982,3983,3984,3985,3986,3987,3988,3989,3990,3991,3992,3993,3994,3995,3996,3997,3998,3999,4000,4001,4002,4003,4004,4005,4006,4007,4008,4009,4010,4011,4012,4013,4014,4015,4016,4017,4018,4019,4020,4021,4022,4023,4024,4025,4026,4027,4028,4029,4030,4031,4032,4033,4034,4035,4036,4037,4038,4039,4040,4041,4042,4043,4044,4045,4046,4047,4048,4049,4050,4051,4052,4053,4054,4055,4056,4057,4058,4059,4060,4061,4062,4063,4064,4065,4066,4067,4068,4069,4070,4071,4072,4073,4074,4075,4076,4077,4078,4079,4080,4081,4082,4083,4084,4085,4086,4087,4088,4089,4090,4091,4092,4093,4094,4095,4096,4097,4098,4099,4100,4101,4102,4103,4104,4105,4106,4107,4108,4109,4110,4111,4112,4113,4114,4115,4116,4117,4118,4119,4120,4121,4122,4123,4124,4125,4126,4127,4128,4129,4130,4131,4132,4133,4134,4135,4136,4137,4138,4139,4140,4141,4142,4143,4144,4145,4146,4147,4148,4149,4150,4151,4152,4153,4154,4155,4156,4157,4158,4159,4160,4161,4162,4163,4164,4165,4166,4167,4168,4169,4170,4171,4172,4173,4174,4175,4176,4177,4178,4179,4180,4181,4182,4183,4184,4185,4186,4187,4188,4189,4190,4191,4192,4193,4194,4195,4196,4197,4198,4199,4200,4201,4202,4203,4204,4205,4206,4207,4208,4209,4210,4211,4212,4213,4214,4215,4216,4217,4218,4219,4220,4221,4222,4223,4224,4225,4226,4227,4228,4229,4230,4231,4232,4233,4234,4235,4236,4237,4238,4239,4240,4241,4242,4243,4244,4245,4246,4247,4248,4249,4250,4251,4252,4253,4254,4255,4256,4257,4258,4259,4260,4261,4262,4263,4264,4265,4266,4267,4268,4269,4270,4271,4272,4273,4274,4275,4276,4277,4278,4279,4280,4281,4282,4283,4284,4285,4286,4287,4288,4289,4290,4291,4292,4293,4294,4295,4296,4297,4298,4299,4300,4301,4302,4303,4304,4305,4306,4307,4308,4309,4310,4311,4312,4313,4314,4315,4316,4317,4318,4319,4320,4321,4322,4323,4324,4325,4326,4327,4328,4329,4330,4331,4332,4333,4334,4335,4336,4337,4338,4339,4340,4341,4342,4343,4344,4345,4346,4347,4348,4349,4350,4351,4352,4353,4354,4355,4356,4357,4358,4359,4360,4361,4362,4363,4364,4365,4366,4367,4368,4369,4370,4371,4372,4373,4374,4375,4376,4377,4378,4379,4380,4381,4382,4383,4384,4385,4386,4387,4388,4389,4390,4391,4392,4393,4394,4395,4396,4397,4398,4399,4400,4401,4402,4403,4404,4405,4406,4407,4408,4409,4410,4411,4412,4413,4414,4415,4416,4417,4418,4419,4420,4421,4422,4423,4424,4425,4426,4427,4428,4429,4430,4431,4432,4433,4434,4435,4436,4437,4438,4439,4440,4441,4442,4443,4444,4445,4446,4447,4448,4449,4450,4451,4452,4453,4454,4455,4456,4457,4458,4459,4460,4461,4462,4463,4464,4465,4466,4467,4468,4469,4470,4471,4472,4473,4474,4475,4476,4477,4478,4479,4480,4481,4482,4483,4484,4485,4486,4487,4488,4489,4490,4491,4492,4493,4494,4495,4496,4497,4498,4499,4500,4501,4502,4503,4504,4505,4506,4507,4508,4509,4510,4511,4512,4513,4514,4515,4516,4517,4518,4519,4520,4521,4522,4523,4524,4525,4526,4527,4528,4529,4530,4531,4532,4533,4534,4535,4536,4537,4538,4539,4540,4541,4542,4543,4544,4545,4546,4547,4548,4549,4550,4551,4552,4553,4554,4555,4556,4557,4558,4559,4560,4561,4562,4563,4564,4565,4566,4567,4568,4569,4570,4571,4572,4573,4574,4575,4576,4577,4578,4579,4580,4581,4582,4583,4584,4585,4586,4587,4588,4589,4590,4591,4592,4593,4594,4595,4596,4597,4598,4599,4600,4601,4602,4603,4604,4605,4606,4607,4608,4609,4610,4611,4612,4613,4614,4615,4616,4617,4618,4619,4620,4621,4622,4623,4624,4625,4626,4627,4628,4629,4630,4631,4632,4633,4634,4635,4636,4637,4638,4639,4640,4641,4642,4643,4644,4645,4646,4647,4648,4649,4650,4651,4652,4653,4654,4655,4656,4657,4658,4659,4660,4661,4662,4663,4664,4665,4666,4667,4668,4669,4670,4671,4672,4673,4674,4675,4676,4677,4678,4679,4680,4681,4682,4683,4684,4685,4686,4687,4688,4689,4690,4691,4692,4693,4694,4695,4696,4697,4698,4699,4700,4701,4702,4703,4704,4705,4706,4707,4708,4709,4710,4711,4712,4713,4714,4715,4716,4717,4718,4719,4720,4721,4722,4723,4724,4725,4726,4727,4728,4729,4730,4731,4732,4733,4734,4735,4736,4737,4738,4739,4740,4741,4742,4743,4744,4745,4746,4747,4748,4749,4750,4751,4752,4753,4754,4755,4756,4757,4758,4759,4760,4761,4762,4763,4764,4765,4766,4767,4768,4769,4770,4771,4772,4773,4774,4775,4776,4777,4778,4779,4780,4781,4782,4783,4784,4785,4786,4787,4788,4789,4790,4791,4792,4793,4794,4795,4796,4797,4798,4799,4800,4801,4802,4803,4804,4805,4806,4807,4808,4809,4810,4811,4812,4813,4814,4815,4816,4817,4818,4819,4820,4821,4822,4823,4824,4825,4826,4827,4828,4829,4830,4831,4832,4833,4834,4835,4836,4837,4838,4839,4840,4841,4842,4843,4844,4845,4846,4847,4848,4849,4850,4851,4852,4853,4854,4855,4856,4857,4858,4859,4860,4861,4862,4863,4864,4865,4866,4867,4868,4869,4870,4871,4872,4873,4874,4875,4876,4877,4878,4879,4880,4881,4882,4883,4884,4885,4886,4887,4888,4889,4890,4891,4892,4893,4894,4895,4896,4897,4898,4899,4900,4901,4902,4903,4904,4905,4906,4907,4908,4909,4910,4911,4912,4913,4914,4915,4916,4917,4918,4919,4920,4921,4922,4923,4924,4925,4926,4927,4928,4929,4930,4931,4932,4933,4934,4935,4936,4937,4938,4939,4940,4941,4942,4943,4944,4945,4946,4947,4948,4949,4950,4951,4952,4953,4954,4955,4956,4957,4958,4959,4960,4961,4962,4963,4964,4965,4966,4967,4968,4969,4970,4971,4972,4973,4974,4975,4976,4977,4978,4979,4980,4981,4982,4983,4984,4985,4986,4987,4988,4989,4990,4991,4992,4993,4994,4995,4996,4997,4998,4999,5000,5001,5002,5003,5004,5005,5006,5007,5008,5009,5010,5011,5013,5014,5015,5016,5017,5018,5019,5020,5021,5022,5023,5024,5025,5026,5027,5028,5029,5030,5031,5032,5033,5034,5035,5036,5037,5038,5039,5040,5041,5042,5043,5044,5045,5046,5047,5048,5049,5050,5051,5052,5053,5054,5055,5056,5057,5058,5059,5060,5061,5062,5063,5064,5065,5066,5067,5068,5069,5070,5071,5072,5073,5074,5075,5076,5077,5078,5079,5080,5081,5082,5083,5084,5085,5086,5087,5088,5089,5090,5091,5092,5093,5094,5095,5096,5097,5098,5099,5100,5101,5102,5103,5104,5105,5106,5107,5108,5109,5110,5111,5112,5113,5114,5115,5116,5117,5118,5119,5120,5121,5122,5123,5124,5125,5126,5127,5128,5129,5130,5131,5132,5133,5134,5135,5136,5137,5138,5139,5140,5141,5142,5143,5144,5145,5146,5147,5148,5149,5150,5151,5152,5153,5154,5155,5156,5157,5158,5159,5160,5161,5162,5163,5164,5165,5166,5167,5169,5170,5171,5172,5173,5174,5175,5176,5177,5178,5179,5180,5181,5182,5183,5184,5185,5186,5187,5188,5189,5190,5191,5192,5193,5194,5195,5196,5197,5198,5199,5200,5201,5202,5203,5204,5205,5206,5208,5209,5210,5211,5212,5213,5214,5215,5216,5217,5218,5219,5220,5221,5222,5223,5224,5225,5226,5227,5228,5229,5230,5231,5232,5233,5234,5235,5236,5237,5238,5239,5240,5241,5242,5243,5244,5245,5246,5247,5248,5249,5250,5251,5252,5253,5254,5255,5256,5257,5258,5259,5260,5261,5262,5263,5264,5265,5266,5267,5268,5269,5270,5271,5272,5273,5274,5275,5276,5278,5279,5280,5281,5282,5283,5284,5285,5287,5288,5289,5290,5291,5292,5293,5294,5295,5296,5297,5299,5300,5301,5302,5304,5305,5306,5307,5308,5310,5311,5312,5313,5314,5315,5316,5317,5318,5319,5320,5321,5322,5323,5324,5325,5326,5327,5328,5329,5330,5331,5332,5333,5334,5335,5336,5337,5338,5339,5340,5341,5342,5343,5344,5345,5346,5347,5348,5349,5351,5352,5353,5354,5355,5356,5357,5358,5359,5360,5361,5362,5363,5364,5365,5366,5367,5368,5369,5370,5371,5372,5373,5374,5375,5376,5377,5378,5379,5380,5381,5382,5383,5384,5385,5386,5387,5388,5389,5390,5391,5392,5393,5394,5395,5396,5397,5398,5399,5400,5401,5402,5403,5404,5405,5406,5407,5408,5409,5410,5411,5413,5414,5415,5416,5417,5418,5419,5420,5421,5422,5423,5424,5425,5426,5427,5428,5429,5430,5431,5433,5434,5435,5436,5437,5438,5439,5440,5441,5442,5443,5444,5445,5446,5447,5448,5449,5450,5451,5452,5454,5455,5456,5457,5458,5459,5460,5461,5462,5463,5464,5465,5466,5467,5468,5469,5470,5471,5473,5474,5475,5477,5478,5479,5481,5482,5483,5484,5486,5487,5488,5489,5490,5491,5492,5493,5494,5495,5496,5497,5498,5499,5500,5501,5502,5503,5504,5505,5506,5508,5509,5510,5511,5512,5514,5515,5516,5517,5518,5519,5520,5522,5523,5524,5525,5526,5527,5528,5529,5531,5532,5533,5534,5535,5536,5537,5538,5539,5540,5541,5542,5543,5544,5545,5547,5548,5550,5552,5553,5554,5556,5557,5558,5559,5560,5561,5562,5565,5566,5567,5568,5569,5570,5571,5572,5573,5574,5575,5576,5578,5581,5583,5584,5586,5587,5588,5589,5590,5592,5593,5594,5595,5598,5599,5600,5601,5602,5603,5604,5605,5606,5607,5608,5609,5610,5611,5613,5614,5615,5617,5618,5620,5622,5623,5624,5626,5627,5628,5630,5631,5632,5633,5634,5635,5636,5638,5639,5640,5642,5644,5645,5647,5648,5649,5650,5651,5652,5653,5654,5655,5656,5657,5658,5659,5660,5661,5664,5665,5666,5667,5668,5670,5672,5674,5675,5676,5677,5678,5679,5681,5682,5683,5684,5685,5686,5687,5688,5689,5690,5691,5692,5693,5694,5695,5696,5697,5698,5699,5702,5703,5704,5706,5707,5708,5709,5712,5713,5716,5717,5718,5719,5720,5721,5723,5724,5725,5726,5727,5728,5729,5730,5731,5732,5733,5734,5737,5739,5740,5741,5742,5743,5744,5746,5747,5748,5751,5752,5753,5755,5756,5757,5758,5759,5760,5761,5762,5763,5764,5766,5769,5770,5771,5772,5773,5774,5775,5777,5778,5780,5781,5782,5785,5786,5787,5788,5790,5791,5792,5793,5795,5796,5797,5798,5799,5800,5801,5803,5804,5805,5807,5808,5811,5812,5813,5814,5815,5817,5823,5824,5825,5826,5827,5829,5830,5831,5834,5835,5836,5838,5839,5840,5842,5843,5845,5846,5848,5850,5851,5852,5854,5855,5857,5858,5860,5861,5862,5863,5864,5865,5866,5867,5868,5870,5873,5874,5876,5877,5879,5881,5882,5884,5885,5887,5889,5890,5891,5892,5893,5894,5895,5896,5898,5900,5902,5904,5907,5908,5909,5911,5913,5914,5915,5916,5919,5920,5921,5922,5924,5925,5926,5927,5928,5929,5930,5933,5934,5936,5937,5938,5939,5940,5941,5943,5944,5945,5946,5947,5948,5949,5950,5951,5952,5954,5955,5956,5958,5962,5963,5964,5965,5967,5969,5970,5971,5972,5973,5976,5978,5979,5980,5982,5983,5985,5986,5987,5989,5990,5991,5993,5994,5995,5996,5997,5999,6000,6002,6003,6005,6006,6007,6008,6009,6012,6014,6015,6017,6018,6019,6020,6021,6025,6026,6027,6032,6033,6034,6036,6037,6038,6040,6042,6045,6048,6049,6050,6051,6056,6057,6058,6059,6061,6062,6063,6066,6068,6071,6072,6074,6076,6077,6078,6079,6080,6081,6086,6087,6090,6096,6099,6100,6101,6102,6104,6105,6107,6109,6110,6111,6112,6113,6114,6115,6121,6123,6125,6126,6129,6130,6133,6135,6138,6140,6143,6146,6147,6149,6150,6151,6157,6159,6160,6163,6164,6165,6167,6168,6169,6170,6175,6178,6179,6181,6182,6183,6184,6187,6190,6191,6193,6194,6195,6198,6202,6203,6206,6209,6210,6211,6213,6215,6216,6218,6220,6221,6222,6223,6226,6227,6228,6230,6231,6232,6233,6234,6237,6240,6243,6245,6251,6253,6255,6257,6258,6259,6260,6262,6263,6264,6265,6267,6268,6269,6271,6276,6277,6281,6282,6283,6284,6285,6286,6289,6292,6295,6298,6299,6300,6301,6304,6305,6307,6308,6310,6317,6321,6322,6325,6327,6328,6329,6331,6332,6334,6337,6338,6340,6341,6343,6344,6345,6346,6351,6352,6353,6355,6356,6358,6359,6360,6363,6366,6370,6371,6374,6375,6377,6378,6380,6381,6382,6385,6386,6387,6390,6398,6399,6402,6403,6404,6406,6407,6408,6411,6412,6413,6414,6416,6420,6421,6424,6427,6428,6432,6435,6436,6437,6439,6448,6450,6452,6453,6454,6456,6458,6459,6462,6463,6464,6465,6467,6470,6473,6474,6478,6479,6480,6483,6484,6486,6487,6490,6491,6495,6496,6497,6498,6500,6502,6504,6506,6508,6509,6511,6512,6514,6515,6517,6529,6533,6534,6535,6537,6540,6542,6545,6547,6548,6558,6561,6578,6579,6580,6584,6585,6587,6588,6589,6590,6591,6592,6597,6600,6601,6605,6607,6608,6611,6612,6613,6614,6619,6620,6622,6623,6625,6627,6628,6630,6631,6632,6640,6643,6645,6647,6648,6651,6652,6654,6655,6657,6658,6659,6665,6669,6672,6673,6674,6676,6678,6682,6683,6687,6690,6692,6693,6694,6696,6701,6709,6711,6720,6722,6726,6727,6729,6732,6735,6738,6741,6742,6748,6756,6766,6767,6771,6774,6775,6778,6781,6782,6783,6786,6790,6792,6794,6797,6801,6804,6808,6809,6811,6813,6816,6817,6821,6823,6825,6827,6829,6830,6832,6833,6845,6846,6847,6848,6852,6855,6857,6861,6865,6866,6868,6870,6873,6876,6879,6882,6884,6887,6889,6891,6894,6896,6901,6903,6916,6919,6920,6922,6923,6925,6927,6928,6931,6934,6939,6941,6943,6944,6946,6951,6952,6963,6968,6969,6970,6974,6980,6982,6987,6990,6993,6995,7004,7007,7008,7011,7013,7015,7018,7020,7021,7024,7028,7030,7034,7036,7042,7044,7046,7050,7058,7061,7064,7067,7069,7070,7071,7072,7074,7076,7077,7078,7079,7080,7081,7087,7090,7091,7092,7094,7096,7097,7100,7101,7102,7105,7108,7120,7122,7123,7127,7128,7129,7133,7134,7136,7143,7147,7151,7152,7153,7155,7159,7160,7161,7162,7168,7169,7170,7173,7174,7178,7182,7185,7187,7188,7191,7195,7196,7197,7198,7201,7202,7203,7208,7212,7213,7214,7217,7224,7225,7226,7227,7235,7238,7244,7251,7252,7253,7254,7256,7257,7261,7278,7280,7285,7295,7296,7298,7300,7302,7304,7305,7307,7308,7309,7312,7313,7317,7321,7323,7324,7329,7330,7333,7337,7341,7345,7346,7347,7350,7351,7352,7353,7354,7356,7363,7364,7368,7371,7372,7373,7378,7382,7383,7390,7392,7393,7396,7397,7398,7402,7413,7414,7418,7426,7427,7429,7438,7440,7442,7443,7445,7447,7452,7453,7454,7455,7461,7462,7467,7469,7471,7480,7484,7486,7488,7491,7494,7498,7500,7502,7503,7505,7508,7509,7510,7516,7523,7527,7533,7534,7537,7541,7542,7545,7551,7556,7558,7560,7561,7575,7576,7581,7583,7591,7593,7595,7597,7598,7600,7603,7605,7606,7607,7610,7611,7612,7616,7617,7618,7620,7621,7623,7626,7627,7628,7634,7639,7641,7643,7650,7658,7661,7662,7664,7668,7675,7678,7679,7680,7682,7686,7687,7689,7693,7694,7698,7702,7705,7706,7717,7722,7728,7732,7734,7735,7740,7743,7745,7753,7758,7761,7762,7764,7772,7774,7775,7776,7780,7786,7787,7792,7801,7803,7804,7813,7817,7822,7827,7830,7832,7833,7839,7842,7847,7851,7852,7856,7861,7864,7877,7879,7885,7891,7899,7902,7905,7909,7922,7929,7935,7941,7948,7949,7954,7957,7972,7973,7980,7986,7992,7998,8007,8008,8009,8028,8029,8037,8039,8055,8057,8062,8065,8066,8069,8072,8078,8083,8095,8099,8104,8115,8121,8131,8133,8142,8147,8150,8156,8159,8160,8164,8173,8181,8183,8196,8203,8216,8221,8224,8227,8228,8233,8245,8251,8252,8256,8257,8260,8276,8280,8283,8299,8300,8302,8305,8311,8324,8325,8330,8338,8341,8354,8364,8369,8371,8379,8381,8384,8405,8419,8423,8436,8440,8442,8446,8448,8459,8462,8463,8465,8491,8499,8504,8505,8509,8511,8516,8521,8535,8538,8550,8557,8572,8573,8581,8595,8601,8640,8641,8651,8659,8666,8667,8683,8688,8725,8731,8736,8738,8739,8741,8748,8749,8752,8759,8761,8762,8777,8784,8789,8790,8793,8795,8816,8831,8836,8838,8843,8846,8851,8857,8875,8879,8892,8896,8898,8920,8934,8941,8943,8951,8963,8964,8971,8983,8990,8994,8995,9004,9005,9008,9011,9018,9029,9031,9048,9052,9056,9085,9091,9102,9103,9107,9109,9119,9120,9128,9132,9134,9136,9139,9145,9147,9165,9172,9175,9194,9206,9210,9214,9217,9219,9221,9225,9226,9229,9232,9259,9262,9270,9271,9277,9282,9283,9284,9288,9289,9291,9292,9306,9316,9324,9331,9337,9338,9358,9387,9389,9401,9407,9431,9444,9451,9454,9472,9473,9491,9511,9516,9523,9531,9551,9562,9566,9574,9578,9580,9584,9601,9632,9636,9638,9654,9655,9659,9666,9668,9688,9696,9704,9713,9721,9748,9760,9761,9775,9779,9783,9789,9797,9799,9802,9803,9806,9811,9817,9824,9841,9847,9864,9866,9883,9904,9911,9912,9917,9930,9931,9951,9979,9980,9984,9986,9990,9996,10017,10024,10027,10034,10052,10072,10077,10090,10099,10103,10104,10106,10116,10150,10152,10172,10176,10216,10218,10223,10236,10268,10273,10284,10288,10302,10325,10335,10399,10418,10429,10432,10441,10453,10470,10482,10488,10493,10494,10504,10509,10513,10519,10534,10557,10567,10571,10572,10612,10613,10647,10664,10689,10710,10718,10724,10727,10743,10784,10794,10803,10809,10811,10819,10822,10824,10829,10850,10867,10876,10920,10930,10934,10936,10938,10940,10945,10971,10980,10981,10990,10998,11007,11032,11043,11050,11060,11066,11087,11093,11104,11107,11110,11123,11150,11187,11201,11206,11211,11231,11242,11257,11260,11266,11271,11279,11295,11296,11312,11334,11381,11399,11451,11455,11474,11478,11493,11503,11517,11544,11623,11644,11654,11668,11672,11728,11739,11744,11745,11747,11758,11774,11800,11804,11833,11871,11886,11904,11921,11939,11972,11977,11992,12001,12035,12076,12079,12085,12097,12106,12107,12118,12170,12171,12185,12196,12198,12200,12207,12231,12303,12305,12318,12344,12360,12403,12414,12442,12463,12513,12531,12539,12575,12584,12624,12663,12676,12689,12699,12740,12741,12743,12746,12794,12819,12835,12836,12847,12881,12914,12920,12942,12997,13014,13048,13069,13102,13128,13144,13177,13192,13211,13255,13312,13325,13382,13383,13401,13451,13456,13529,13545,13572,13575,13580,13599,13601,13622,13656,13714,13719,13736,13739,13753,13758,13761,13797,13839,13920,13935,13960,13974,14009,14014,14059,14069,14095,14101,14184,14200,14216,14245,14257,14289,14312,14313,14333,14344,14387,14405,14414,14518,14526,14623,14632,14651,14706,14715,14724,14807,14847,14852,14928,14942,14963,14997,15036,15126,15160,15273,15303,15317,15436,15439,15455,15472,15504,15525,15535,15548,15557,15561,15942,16023,16357,16435,16524,16536,16542,16595,16607,16621,16624,16631,16708,16806,16910,17005,17032,17033,17061,17305,17317,18107,18260,18554,18647,19070,19274,19558,19562,20235,21116,21663,22415,22543,22690,22732,23190,23299,23364,23422,24470,25155,25411,27591,29972,30192,30498,30557,30713,30839,30950,31954,33814,35153,35311,36381,38281,38615,39916,40610,68809,92257,103107,120822,144822,159073,184647,190559,193355,213554,278349,314372 |
| Count | 2,2,2,3,1,7,1,1,1,9,9,29,11,10,28,8,7,26,7,9,22,14,7,12,8,7,17,4,9,14,6,6,15,12,7,15,7,10,15,11,5,14,9,18,16,13,7,17,5,14,19,7,11,16,3,11,9,13,15,12,12,21,15,11,10,17,6,14,20,13,19,15,10,15,22,13,18,16,8,20,20,12,11,14,10,11,18,11,17,20,12,13,12,13,16,13,12,24,21,13,15,16,12,19,24,5,13,17,7,21,15,18,11,23,17,12,23,13,24,19,12,13,13,18,18,15,14,25,19,11,15,14,10,25,19,12,21,12,10,19,10,21,14,12,16,14,12,14,18,8,12,25,15,9,10,11,14,20,18,9,16,18,21,21,16,10,19,13,23,20,18,10,18,13,12,17,16,21,20,21,9,9,16,11,14,10,13,16,13,11,14,12,12,15,13,10,15,13,18,16,17,13,13,19,11,13,14,16,18,21,9,12,14,16,12,8,12,15,12,16,16,13,16,27,11,12,16,11,20,17,13,14,16,15,11,15,16,12,12,9,19,19,16,7,18,17,11,21,13,7,12,13,8,13,10,9,18,14,10,14,13,10,18,20,16,18,9,25,23,10,10,14,17,8,11,10,13,20,10,8,12,7,13,20,14,15,10,11,13,18,15,10,12,19,14,10,11,13,11,17,12,10,12,12,6,13,15,11,10,5,14,14,24,29,15,7,16,15,11,15,10,9,17,11,12,12,12,11,15,12,11,16,12,6,15,26,17,10,19,9,14,11,18,12,13,9,23,13,21,15,22,16,14,14,21,18,16,34,44,16,12,16,7,13,16,17,13,16,15,13,17,16,17,16,11,12,21,19,20,22,20,13,12,17,17,15,24,18,17,19,22,15,14,15,19,20,16,14,19,22,22,16,36,89,25,5,16,12,15,16,13,21,22,19,15,20,24,16,14,16,19,27,21,19,21,19,17,21,22,25,19,15,16,19,21,19,23,18,16,22,22,14,16,22,27,23,29,79,95,48,33,22,20,17,20,22,26,16,20,26,28,24,23,16,13,30,22,16,21,18,24,27,23,18,20,22,26,16,25,19,29,22,24,27,24,21,32,19,22,29,20,32,69,172,24,19,24,24,23,20,17,22,29,15,15,20,23,15,21,29,23,20,18,25,25,19,27,36,29,23,27,30,29,30,33,32,35,36,34,30,33,42,28,25,31,48,36,90,212,45,22,26,20,29,26,29,19,35,25,26,22,30,25,30,22,23,23,25,23,35,29,26,30,30,36,27,35,26,29,30,30,27,36,29,34,36,38,36,43,30,31,37,108,296,41,24,23,24,16,25,19,37,29,23,28,27,30,31,33,21,32,27,34,26,35,32,40,31,38,30,35,33,33,34,36,38,40,36,49,46,55,41,46,37,49,42,57,118,347,33,24,28,32,32,34,16,25,35,26,25,44,21,33,35,36,35,32,39,25,27,33,35,30,40,36,52,41,48,40,38,41,40,48,47,53,52,54,52,41,48,56,47,118,402,41,27,31,39,27,35,37,38,22,36,39,59,37,26,22,34,38,29,42,37,36,39,38,43,42,44,49,47,31,48,44,52,37,57,54,51,72,62,53,44,51,70,67,100,424,75,34,30,37,35,41,40,35,29,31,37,40,37,43,39,37,50,47,47,38,44,33,44,51,47,34,36,49,61,46,45,44,63,54,57,62,66,66,50,53,58,60,72,115,474,49,26,25,38,34,49,32,39,35,44,40,38,43,49,33,39,38,39,54,38,44,55,37,48,66,53,52,49,59,52,45,55,51,80,53,55,61,69,51,66,60,74,75,132,516,66,44,35,42,39,38,38,38,48,43,39,46,41,34,38,53,58,59,54,47,47,46,40,53,46,49,45,59,58,49,51,46,65,67,83,73,79,65,65,70,66,87,79,182,507,61,36,45,38,53,54,48,57,51,62,56,54,51,53,52,33,50,44,58,47,66,50,63,65,52,64,53,65,57,53,62,69,63,80,68,73,67,83,56,67,64,70,81,187,461,73,42,45,48,54,44,42,49,63,54,52,49,45,46,54,46,57,56,58,56,68,55,52,55,55,63,52,55,72,67,67,76,61,65,70,80,76,80,67,88,71,64,71,194,526,63,49,40,53,59,54,47,55,43,51,46,54,51,44,67,53,57,55,66,52,50,57,54,61,58,56,71,76,69,66,62,72,68,95,83,100,76,112,86,71,72,89,96,232,536,78,60,44,46,48,46,59,52,47,58,50,52,56,47,58,67,40,65,56,61,70,62,76,75,87,55,79,75,77,59,60,70,72,96,70,77,101,103,74,91,75,97,100,232,523,75,50,39,61,58,37,55,69,49,65,54,60,65,59,54,69,56,60,65,59,72,70,67,66,82,60,76,91,85,72,69,74,94,74,83,85,96,97,89,83,90,80,82,270,499,68,54,47,52,46,65,66,62,63,80,53,67,44,68,57,69,64,64,66,61,68,53,72,70,82,83,80,96,72,63,84,99,74,86,89,131,86,102,95,90,96,100,113,270,490,79,51,50,70,48,58,47,59,66,70,64,56,72,52,73,66,71,67,81,57,78,75,73,80,83,82,87,75,78,72,94,98,88,96,87,119,101,114,84,79,93,99,104,309,466,63,55,71,58,45,51,54,68,65,65,77,72,66,61,61,94,75,78,77,85,74,69,85,72,73,85,86,88,97,84,77,86,104,100,101,115,93,100,99,100,107,97,89,298,468,62,54,78,71,82,85,56,73,76,69,70,61,79,78,83,73,77,74,89,78,60,96,71,81,82,86,93,105,98,71,95,91,103,116,98,106,123,102,108,83,111,101,131,303,445,72,69,65,62,79,68,74,85,63,71,75,78,75,90,82,71,88,87,96,86,95,103,91,111,103,111,99,100,120,90,109,107,117,119,131,161,103,114,109,110,119,107,105,318,453,85,80,74,68,65,88,76,89,92,85,65,90,88,80,95,116,76,110,109,96,84,104,96,94,111,99,103,95,111,126,120,116,115,113,136,166,114,118,107,120,106,119,112,305,438,96,86,79,71,69,65,86,77,77,110,88,88,76,91,75,74,102,92,82,94,82,112,108,112,94,114,110,113,95,119,106,113,130,114,156,165,120,108,92,126,104,115,127,320,408,94,69,93,85,79,90,77,85,81,108,102,103,97,102,100,98,105,100,87,89,103,81,99,120,121,124,95,115,99,119,115,129,137,129,185,211,126,116,103,113,105,103,144,359,371,106,100,79,75,98,79,72,89,87,91,90,86,90,95,105,101,75,104,97,104,99,91,97,103,127,112,128,108,129,137,111,124,118,134,205,186,123,119,119,142,109,125,118,328,389,76,77,102,97,96,79,90,99,91,90,89,102,80,90,82,114,109,103,103,106,113,121,124,125,106,113,112,114,115,127,149,114,112,143,197,168,126,115,120,122,124,116,137,312,379,92,95,90,110,92,97,106,99,93,109,79,70,96,98,106,119,95,103,112,105,110,108,119,117,115,114,111,148,116,135,138,126,145,133,183,161,122,137,101,114,111,133,122,337,357,103,95,97,94,94,82,105,86,106,87,89,118,108,85,90,105,113,122,109,111,132,108,132,123,119,107,128,130,129,113,137,147,118,126,197,198,123,132,123,115,129,130,118,340,323,125,100,94,100,91,111,101,87,116,110,105,96,102,96,113,108,91,123,89,108,122,123,123,142,121,125,142,121,115,133,137,137,128,113,200,182,151,115,112,107,123,145,152,341,330,98,95,112,115,89,105,103,84,97,99,87,85,112,96,89,124,135,118,106,134,111,136,118,142,115,111,143,139,145,126,111,109,142,136,208,181,131,130,136,123,129,120,125,328,305,90,101,94,96,110,111,111,117,107,112,118,114,108,132,130,129,118,127,131,123,110,127,118,143,121,129,155,132,126,139,137,123,131,173,204,175,151,117,102,145,147,140,131,322,291,96,101,108,108,81,108,113,99,120,98,101,110,101,114,107,122,115,110,123,136,117,132,134,157,125,127,116,134,130,136,149,129,128,144,209,202,133,139,144,146,138,116,121,307,300,122,98,97,127,103,113,112,113,102,109,113,114,119,139,116,116,123,122,127,138,140,121,120,135,106,123,127,129,118,132,137,130,128,141,217,187,164,142,151,154,145,146,159,316,271,96,96,112,91,126,123,134,98,94,118,107,113,124,115,107,117,116,128,128,132,151,134,131,162,142,162,137,131,127,151,139,164,145,134,197,161,127,145,142,140,139,152,133,312,264,121,120,120,108,133,105,139,130,118,95,126,130,109,124,132,121,113,137,128,118,137,118,162,159,138,150,163,127,159,149,150,159,154,136,174,184,159,161,135,142,130,126,133,367,314,121,128,122,124,110,147,115,150,126,133,115,118,138,111,132,119,127,139,138,132,156,153,157,138,139,117,149,152,160,161,165,146,163,167,190,179,172,132,142,150,130,162,161,317,235,121,122,118,122,102,121,136,128,128,112,141,154,123,129,125,158,131,130,128,136,152,148,149,149,144,163,137,144,140,151,150,137,184,166,197,198,164,151,149,142,133,147,152,318,250,128,108,121,130,132,151,129,120,131,142,138,114,136,128,124,134,153,133,127,137,150,157,152,151,137,135,151,167,129,148,143,163,156,144,221,182,144,170,178,150,139,160,157,328,270,105,134,133,114,100,128,151,156,129,150,139,137,139,137,139,136,171,162,130,125,168,131,154,161,165,158,131,137,148,160,172,156,168,154,212,168,147,142,164,156,146,170,168,336,260,150,124,151,150,139,119,141,120,154,133,129,120,161,148,132,159,144,154,156,136,156,136,162,168,161,128,164,153,179,146,161,140,166,178,201,199,171,163,148,163,171,156,150,282,231,144,133,131,142,116,136,141,139,136,145,129,131,146,120,163,144,131,158,145,169,171,164,144,174,134,156,164,155,168,152,172,166,147,169,237,205,162,173,163,160,158,152,160,320,216,137,142,134,138,152,141,150,156,138,135,157,155,144,151,160,151,190,125,151,148,148,184,154,202,148,173,165,164,163,169,178,155,165,173,197,176,179,162,162,145,166,154,156,343,221,131,138,144,137,128,155,152,157,179,161,152,135,158,150,188,144,176,161,159,150,144,151,165,173,145,176,163,167,166,181,141,158,170,174,221,184,168,134,178,171,173,171,186,320,245,157,153,151,139,159,160,165,147,158,150,141,153,153,170,166,154,153,147,143,151,154,170,161,189,184,187,181,179,145,150,177,160,184,184,270,194,200,160,183,160,158,173,186,332,208,149,149,151,167,145,135,138,159,148,136,154,151,176,156,166,159,181,164,155,158,154,170,181,174,202,178,175,183,164,152,191,187,182,199,232,203,181,161,199,163,170,190,178,331,205,137,160,127,160,167,153,162,146,178,153,152,153,178,175,171,165,150,143,167,178,141,160,144,178,183,184,184,186,183,178,198,175,180,170,234,178,161,175,180,182,184,186,164,324,230,154,143,156,158,142,156,181,173,162,206,169,175,167,163,160,174,192,169,139,172,153,191,161,182,162,176,163,190,197,173,190,184,173,190,233,182,208,170,163,150,139,175,186,291,235,159,163,150,138,161,172,150,187,168,156,166,164,162,152,176,186,159,190,181,157,175,178,177,172,164,194,168,176,163,184,173,146,192,182,222,176,173,178,174,170,176,157,197,308,229,178,132,142,156,172,158,188,181,161,164,160,154,167,173,170,153,179,176,168,185,170,188,186,158,189,177,177,177,192,179,175,175,174,191,228,205,180,194,164,177,188,205,186,329,205,147,159,159,203,164,168,169,156,178,173,166,174,170,153,163,182,188,171,187,175,181,174,192,199,174,162,198,214,165,172,178,192,185,173,204,224,184,199,183,194,175,199,175,325,222,178,149,174,175,180,166,166,179,185,175,166,167,164,158,162,160,172,169,165,183,175,167,189,202,193,198,171,171,193,172,205,188,171,202,235,196,198,166,192,186,175,181,225,310,232,138,172,161,162,168,167,165,176,170,158,187,176,181,173,194,156,175,171,178,166,198,193,179,158,201,223,196,193,183,178,184,190,177,186,207,209,183,202,194,158,189,170,212,301,212,196,166,151,177,150,164,171,172,163,161,162,186,190,181,190,187,185,176,175,180,210,186,188,172,195,188,205,209,189,167,200,176,189,168,209,188,159,163,175,182,188,178,162,304,207,166,179,168,158,149,174,172,172,170,162,157,181,178,185,189,168,169,169,187,179,209,211,194,185,183,207,179,178,197,205,184,170,201,164,228,180,235,172,198,170,179,187,193,260,207,156,173,174,162,176,179,162,173,184,186,141,180,154,166,160,189,167,183,155,175,188,180,178,180,176,196,196,142,167,208,194,178,186,201,218,192,183,157,171,177,159,197,173,291,191,166,139,173,175,154,177,165,185,163,154,164,142,167,172,174,166,190,176,191,166,171,199,203,185,176,193,202,190,176,200,174,172,181,163,240,196,175,178,176,190,142,171,203,285,227,180,143,186,170,159,137,171,158,165,166,158,173,157,164,188,173,193,190,164,191,199,199,231,204,184,196,204,203,204,182,155,185,181,189,212,186,168,157,169,168,176,156,181,274,210,161,167,192,141,171,173,185,165,158,170,167,144,172,185,184,198,169,179,177,165,186,199,160,182,186,181,187,176,179,184,194,184,189,168,200,187,178,147,183,178,165,171,159,308,196,176,157,177,190,177,164,189,158,162,176,177,194,141,179,193,173,184,169,185,180,172,182,174,173,183,165,185,180,158,194,149,181,165,165,206,173,204,178,175,158,171,191,165,260,174,176,181,190,166,161,141,175,160,181,171,189,155,170,144,166,165,170,191,173,163,188,177,179,183,191,191,171,179,185,208,192,176,174,176,241,191,196,183,190,165,172,170,159,259,197,143,162,140,143,150,173,172,182,169,187,173,154,173,168,172,178,154,156,170,186,168,158,184,178,180,163,192,169,170,187,190,177,169,173,172,173,162,158,178,187,165,186,176,268,189,155,148,156,146,162,167,175,180,167,171,152,172,157,144,170,159,179,148,176,196,168,190,141,178,187,193,161,172,171,177,177,169,187,180,191,174,179,155,170,170,174,171,160,256,169,155,154,144,185,161,150,165,169,159,151,153,145,159,147,154,150,152,178,172,177,171,171,169,158,173,169,171,173,154,168,166,154,146,157,192,170,169,172,173,172,135,192,171,246,167,137,156,159,141,139,138,168,166,149,155,137,130,171,160,159,146,160,163,166,178,155,173,182,167,158,154,179,157,158,176,141,176,156,170,187,167,168,155,155,161,154,188,167,239,155,142,139,149,148,147,149,155,143,136,146,165,155,156,153,152,147,180,163,135,151,155,175,152,136,138,166,168,155,154,165,147,162,159,159,189,159,157,159,155,154,162,153,123,232,133,148,119,133,152,160,157,145,164,147,139,176,152,150,170,146,148,159,154,154,153,159,148,155,163,166,167,162,163,158,150,159,170,166,164,174,158,177,117,164,154,145,149,148,200,155,131,168,165,140,133,160,147,141,136,152,133,159,152,139,139,132,133,151,123,161,146,159,143,136,143,153,163,141,148,141,167,152,139,128,159,151,150,136,137,141,137,144,135,215,127,111,144,119,124,135,150,130,142,119,157,142,132,146,132,146,151,118,128,131,143,143,138,136,158,143,126,142,129,131,127,122,130,124,147,171,150,145,157,132,141,167,140,156,214,149,111,126,138,128,132,121,140,149,115,131,128,141,120,143,142,135,149,140,150,115,133,144,138,150,127,136,122,149,120,136,128,136,127,109,178,143,150,116,112,154,117,128,128,219,141,126,123,119,116,142,147,142,119,146,110,109,115,127,103,131,131,131,124,139,119,125,127,123,123,147,115,140,123,129,128,116,126,128,134,139,146,115,124,134,139,104,118,111,197,108,106,121,129,134,125,113,114,118,129,123,128,99,120,109,122,124,147,128,124,120,128,132,141,120,152,128,138,144,137,132,114,156,138,105,151,109,112,122,107,122,120,126,137,177,118,104,99,98,117,107,121,122,111,130,103,112,112,102,105,127,101,119,124,116,108,132,114,113,126,123,83,102,140,101,129,118,132,99,111,136,128,105,118,122,110,121,106,130,170,111,109,96,98,84,118,97,127,94,109,109,97,91,103,135,105,114,94,100,117,116,108,89,118,109,115,111,129,106,129,98,98,117,116,93,126,113,107,126,105,129,121,118,108,176,131,103,105,115,128,114,103,109,104,107,110,116,120,101,102,100,112,108,111,104,108,114,105,105,118,112,125,119,97,121,108,109,110,89,93,114,136,115,111,121,112,112,100,113,129,91,94,94,100,70,78,91,90,97,101,92,103,106,100,104,89,102,90,95,80,104,111,98,87,108,112,91,88,100,87,108,83,112,85,106,104,103,103,97,95,82,102,101,90,140,113,81,79,93,89,86,92,106,105,85,101,83,74,86,75,105,84,96,106,72,89,93,67,110,102,94,99,88,102,95,87,83,90,82,98,102,110,79,82,110,88,84,116,87,127,94,84,78,75,69,82,88,93,89,95,86,72,86,80,84,91,97,79,80,83,70,98,87,97,76,84,87,94,92,80,76,88,90,99,84,123,84,94,83,93,76,74,101,96,130,84,74,74,78,89,56,71,67,88,88,92,91,72,82,82,79,103,83,80,79,80,76,79,99,84,118,94,86,81,91,92,85,73,88,81,87,90,74,71,73,70,84,78,65,104,75,74,63,65,79,71,75,85,70,90,73,69,83,71,93,86,70,72,75,85,67,57,90,73,71,74,72,75,82,81,92,80,93,79,67,83,72,68,72,77,75,76,66,67,113,70,71,60,75,67,67,60,70,79,79,77,72,68,62,70,61,77,73,83,91,59,70,78,72,72,83,68,80,70,78,65,75,60,67,71,70,69,83,74,59,68,71,66,68,104,63,66,60,70,66,74,73,51,67,74,69,74,68,56,66,71,81,69,63,72,58,87,60,67,73,86,76,63,59,70,77,62,58,66,61,66,65,56,68,54,73,62,73,56,90,72,57,62,53,64,66,51,67,66,52,62,48,51,80,69,62,66,61,71,63,65,58,63,51,65,54,62,52,58,65,57,68,57,77,72,89,47,74,58,58,61,56,60,59,106,72,55,53,53,44,44,55,54,46,52,54,62,54,54,64,48,55,60,68,58,57,55,52,59,45,61,62,72,63,65,60,53,60,74,72,76,48,63,54,56,41,49,33,52,72,41,46,43,38,54,60,47,54,56,57,59,51,57,61,52,52,36,37,71,62,55,62,62,57,50,56,72,52,56,69,63,55,47,52,26,65,42,63,47,55,59,58,57,44,84,41,53,47,45,58,42,46,52,50,53,54,57,39,44,43,44,44,59,43,53,49,62,44,44,44,53,52,45,38,45,55,51,46,49,49,48,37,47,37,55,46,39,49,47,73,35,45,43,39,40,36,41,38,47,32,50,56,41,39,36,38,49,41,37,36,49,33,52,41,43,40,47,35,43,44,43,39,44,56,33,58,46,35,41,37,43,46,32,38,58,37,40,40,51,35,40,37,32,38,45,44,46,41,45,36,33,34,30,38,51,45,37,34,43,43,29,51,37,51,34,41,40,27,40,47,48,50,45,47,41,45,48,36,37,87,40,37,43,41,41,32,29,36,40,42,36,46,34,37,30,40,47,37,26,28,35,37,42,41,27,33,34,38,37,46,37,39,35,48,39,31,27,39,34,42,28,31,37,36,50,35,29,30,35,26,24,36,38,32,34,31,42,30,45,34,42,35,36,30,34,31,31,30,31,36,29,27,25,34,37,33,32,34,22,47,38,47,30,26,31,34,35,32,27,61,29,27,22,27,27,26,29,38,28,32,37,29,32,25,36,34,35,31,29,22,27,29,30,26,34,34,22,29,44,33,22,38,36,31,24,31,36,29,31,29,34,30,31,30,51,22,28,30,32,25,25,35,30,27,23,28,13,19,31,21,32,30,34,31,27,33,23,29,33,34,24,26,26,38,25,39,32,28,40,20,27,38,26,32,25,20,29,28,24,42,27,20,30,28,29,15,25,21,28,21,28,29,27,34,21,22,29,25,23,31,22,30,25,21,26,25,25,20,24,28,22,28,20,23,20,26,21,21,17,23,21,21,20,27,30,26,25,25,17,16,28,18,21,16,22,18,33,25,20,16,29,25,20,19,17,21,19,27,24,23,21,21,23,33,21,26,19,26,16,24,18,21,26,18,18,19,27,24,16,24,25,27,23,14,18,20,17,11,23,19,21,25,20,19,12,23,24,21,18,21,19,25,20,26,17,16,11,15,16,25,21,16,23,28,31,21,21,16,25,18,19,18,17,15,24,13,18,18,20,11,17,26,16,15,17,27,9,13,14,17,17,20,33,19,14,21,15,26,14,21,16,20,14,19,20,19,20,21,12,10,22,25,15,25,9,21,16,17,18,22,13,13,12,9,23,13,16,17,11,18,8,19,19,11,13,8,12,19,11,11,18,22,24,19,17,17,22,14,15,14,15,20,17,27,13,17,18,19,11,22,9,8,15,15,27,13,12,14,15,14,13,17,13,13,12,12,6,21,18,21,19,16,13,14,21,15,15,13,17,14,22,13,15,14,21,16,11,19,18,14,22,19,18,16,16,14,16,14,17,21,15,10,14,12,4,14,16,9,9,10,21,16,14,12,20,15,16,15,19,16,11,8,16,15,13,11,13,9,20,14,11,15,10,12,15,11,16,12,13,11,17,9,16,10,17,10,14,17,13,6,9,13,14,13,8,6,13,14,16,6,13,15,16,14,10,13,15,8,14,10,18,8,6,12,19,8,15,10,15,11,13,7,12,21,12,16,10,15,12,19,11,6,12,6,13,13,9,12,12,9,6,6,11,7,14,7,12,16,12,4,8,14,7,7,13,11,11,9,17,12,10,11,12,16,12,13,11,7,8,10,12,12,12,15,18,11,6,8,6,7,8,8,10,8,9,11,10,8,11,11,7,17,4,10,7,12,8,5,10,13,15,11,7,14,10,7,16,11,9,11,12,8,4,8,15,5,8,4,5,18,8,12,11,3,7,5,11,7,6,5,9,12,10,10,7,15,10,6,16,8,7,8,6,10,11,12,7,15,6,9,6,9,9,9,8,12,13,14,9,3,6,5,7,2,11,10,6,8,7,9,9,13,4,6,8,14,8,12,8,5,5,8,5,8,2,15,10,7,10,5,10,11,7,13,15,12,8,10,5,7,9,2,7,8,10,7,5,6,10,3,10,5,7,7,8,9,5,8,11,7,8,6,7,13,8,6,4,8,5,9,5,9,6,7,2,5,5,5,3,6,10,6,1,7,4,8,6,8,3,5,4,10,8,11,5,3,3,5,4,5,7,7,4,6,7,2,5,2,9,6,3,9,5,3,1,9,5,8,4,2,10,4,4,5,5,5,7,6,9,11,6,8,2,5,9,5,4,4,11,5,12,4,7,4,9,3,10,6,7,8,2,9,5,7,6,4,4,9,4,6,6,1,11,6,2,4,7,10,5,5,8,3,7,4,8,2,5,5,7,6,5,8,7,9,1,3,5,6,6,3,2,5,6,1,7,5,7,2,7,15,4,6,1,5,3,9,5,9,7,4,10,2,8,7,6,4,8,4,5,5,3,7,8,6,4,4,6,7,4,3,6,7,2,8,1,4,5,4,5,1,5,7,8,3,3,2,6,3,3,4,5,5,5,2,9,3,1,5,9,5,5,5,3,5,9,2,4,7,3,7,4,7,1,6,3,5,4,2,3,3,4,1,2,2,5,3,1,1,4,2,2,3,11,3,6,5,4,3,7,7,3,4,4,1,5,2,8,8,4,3,2,3,5,7,6,4,2,5,2,3,4,4,5,4,1,3,3,3,5,5,3,2,4,4,3,3,2,1,4,3,5,9,5,7,4,3,3,3,10,5,1,2,5,2,9,4,7,5,2,2,5,4,4,3,2,6,4,4,4,5,3,4,3,2,4,2,3,4,2,5,6,2,3,6,2,6,1,7,2,4,2,1,3,2,4,4,8,4,5,2,5,2,4,2,5,3,4,3,1,6,3,2,3,6,2,3,1,1,3,3,1,4,5,4,4,5,3,3,3,5,3,3,4,4,3,1,7,1,2,4,3,2,6,2,6,3,4,2,2,1,6,2,5,1,3,3,1,2,4,1,3,4,2,1,1,1,3,9,5,6,3,1,2,3,5,2,5,5,3,3,3,2,3,3,1,5,2,5,6,3,4,3,4,1,1,3,4,2,5,1,2,2,3,3,3,4,5,4,4,5,1,1,1,2,4,5,4,4,2,1,1,3,3,4,8,3,4,3,1,4,3,2,2,1,4,3,1,4,1,1,2,1,7,1,6,3,1,3,2,2,2,1,2,1,1,1,4,6,4,4,3,4,3,1,3,3,6,2,2,3,3,3,1,1,2,4,4,2,4,1,2,2,1,3,3,2,2,4,1,3,2,3,9,3,2,2,2,3,5,2,2,4,3,1,3,1,1,4,1,2,2,2,3,2,1,4,1,1,3,2,2,2,7,1,1,2,1,3,2,2,1,1,1,2,1,2,1,1,4,2,2,1,2,3,1,3,6,1,3,4,2,5,2,3,2,1,1,1,5,1,4,1,2,2,1,1,1,2,1,4,3,2,2,1,4,1,1,1,1,3,2,2,1,1,1,2,1,2,1,2,2,1,2,4,5,1,1,1,3,2,1,1,4,3,1,1,2,1,3,3,5,3,1,1,2,3,3,1,1,2,2,1,1,2,1,1,4,3,2,1,1,4,3,1,3,1,3,2,3,1,2,2,2,5,2,2,1,2,1,1,1,2,4,2,2,1,1,1,1,2,2,1,1,2,2,1,1,3,2,2,1,2,1,1,1,1,3,1,2,2,2,2,1,2,2,1,1,3,2,2,3,2,2,3,1,1,1,1,3,2,1,1,2,1,2,1,3,2,1,4,2,1,2,1,3,2,2,1,1,2,1,2,3,2,1,1,2,2,1,5,1,1,2,3,1,3,1,3,1,1,1,1,1,2,2,1,1,2,1,1,2,1,1,1,1,1,2,1,2,1,1,1,2,1,1,1,2,1,3,1,1,1,1,1,1,1,2,2,1,2,1,1,2,2,1,3,4,2,1,2,1,2,1,1,1,2,3,2,3,3,1,2,3,1,2,1,1,2,1,1,1,3,1,1,1,1,2,1,1,1,2,2,1,1,1,1,1,2,3,1,1,1,2,2,2,1,3,2,1,2,2,5,2,2,1,2,1,1,1,1,2,1,1,2,1,1,1,2,2,1,1,1,1,1,1,2,3,1,1,1,1,2,1,1,3,2,1,1,2,1,2,1,1,3,2,1,1,1,1,1,1,2,1,1,1,2,2,1,2,2,1,3,1,1,1,2,1,2,1,1,1,1,1,2,2,2,1,2,2,1,2,1,2,2,3,3,1,1,1,1,1,1,1,2,1,2,1,2,1,1,2,2,1,1,1,2,1,1,1,1,1,1,1,1,1,2,2,1,2,2,1,1,1,2,2,2,1,1,2,1,2,2,2,1,2,1,1,1,1,1,1,1,1,1,2,1,1,1,3,2,1,1,1,1,3,1,2,1,2,2,1,1,1,1,1,1,1,2,1,2,1,1,1,1,2,3,2,2,1,2,2,1,1,2,1,1,3,2,1,1,1,1,1,1,2,1,1,1,2,3,1,1,1,2,1,1,2,1,1,1,1,3,1,1,1,1,1,1,2,1,1,3,2,2,1,1,2,1,1,1,1,1,1,1,1,1,1,2,1,1,1,1,1,2,1,1,1,1,2,1,1,1,1,1,2,1,2,1,2,1,1,1,1,1,1,1,2,2,1,1,1,1,1,1,1,1,1,1,1,1,2,1,1,2,5,1,1,1,1,1,1,2,1,1,4,1,2,2,1,1,3,1,1,1,3,1,1,1,1,1,1,1,2,1,1,1,2,1,2,1,1,1,1,1,1,1,3,1,4,2,1,1,1,1,1,1,1,1,2,1,1,1,1,1,1,1,2,1,1,1,1,1,2,1,1,1,1,1,1,1,2,1,1,1,1,1,2,1,2,3,1,1,1,1,1,1,1,2,1,1,1,1,1,2,2,1,1,2,1,2,1,1,2,1,1,1,1,3,1,1,1,1,2,1,1,1,3,1,2,1,2,1,1,2,1,1,1,1,1,1,1,1,1,1,1,1,1,1,1,1,1,1,1,1,1,1,1,2,1,3,1,2,1,1,2,1,1,1,2,2,1,2,1,1,1,1,2,1,1,1,1,2,1,1,1,1,1,1,1,4,2,2,1,1,2,2,1,1,1,1,1,1,1,1,1,1,1,2,1,1,1,1,1,2,2,1,1,2,3,1,2,1,1,2,1,1,1,2,1,3,1,2,2,1,1,1,2,1,2,1,1,1,1,2,1,1,2,1,1,2,2,1,1,1,1,1,1,1,1,1,1,2,1,1,2,1,1,1,1,1,2,1,1,2,1,1,1,1,2,1,2,1,1,1,1,1,2,1,2,1,1,1,1,1,1,2,1,1,1,3,1,1,1,1,2,1,1,1,2,1,1,1,1,1,1,1,1,1,2,1,1,1,2,1,1,1,1,1,1,1,1,3,1,1,1,1,1,1,1,1,2,1,1,3,1,1,2,1,2,1,1,1,1,1,1,1,4,1,1,1,2,1,1,1,1,1,1,1,1,1,1,1,1,1,2,1,1,2,1,1,1,1,1,1,1,1,1,1,1,1,1,1,1,1,1,2,1,1,1,1,1,1,1,1,2,1,1,1,1,2,1,1,1,1,1,1,1,1,1,1,1,1,1,1,1,1,1,1,1,1,1,1,1,1,1,1,3,1,1,1,1,1,1,1,1,1,1,1,1,1,1,1,1,1,2,1,1,1,1,1,2,1,1,1,1,1,1,1,1,1,1,1,1,1,1,1,1,1,1,1,2,1,1,1,1,1,2,1,1,1,2,1,1,2,1,2,2,1,1,1,1,1,1,1,1,1,1,1,1,1,1,1,1,1,1,1,1,1,1,1,1,1,1,1,1,1,1,1,1,1,1,1,1,1,1,1,1,1,1,1,1,1,1,1,1,1,2,1,1,1,1,1,1,2,1,1,1,1,2,1,2,1,1,1,1,1,1,1,1,1,1,1,2,1,1,1,1,1,1,1,1,1,1,1,1,1,1,1,1,1,1,1,1,1,1,1,1,1,1,1,1,2,1,2,1,1,1,2,1,1,1,1,1,1,1,1,1,1,1,1,1,1,1,1,1,1,1,1,1,1,1,1,1,1,1,1,1,1,1,1,1,1,1,1,1,1,1,1,1,1,1,1,1,1,1,1,2,1,1,1,1,1,1,1,1,1,1,1,1,1,1,1,1,1,1,1,1,1,1,1,1,1,1,1,1,1,1,1,1,1,1,1,1,1,1,1,1,1,1,1,1,1,1,1,1,1,1,1,1,1,1,1,1,1,1,1,1,1,1,1,1,1,1,2,1,1,1,1,1,1,1,1,1,1,1,2,1,1,1,1,1,1,1,1,1,1,1,2,1,2,1,1,1,1,1,1,1,1,1,1,1,1,1,1,1,1,1,1,1,1,1,1,1,1,1,1,1,1,1,1,1,1,1,1,2,1,1,1,1,1,2,1,1,1,1,1,1,1,2,1,1,1,1,1,1,1,1,1,1,1,1,1,1,1,1,1,1,1,1,1,1,1,1,1,1,1,1,1,1,1,1,1,1,1,1,2,1,1,1,1,1,1,1,1,1,1,1,1,1,1,1,1,1,1,1,1,2,1,1,1,1,1,1,1,1,1,1,1,1,1,1,1,1,1,1,1,1,1,1,1,1,1,1,1,1,1,1,1,1,1,1,1,1,1,1,1,1,1,1,1,1,1,1,1,1,1,1,1,1,1,1,1,1,1,1,1,2,1,1,1,1,1,1,1,1,1,1,1,1,1,1,1,1,1,1,1,1,1,1,1,1,1,1,1,1,1,1,1,1,1,1,1,1,1,1,1,1,1,1,1,1,1,1,1,1,1,1,1,1,1,1,1,1,1,1,1,1,1,1,1,1,1,1,1,1,1,1,1,1,1,1,1,1,1,1,1,1,1,1,1,1,1,1,1,1,1,1,1,1,1,1,1,1,1,1,1,1,1,1,1,1,1,1,1,1,1,1,1,1,1,1,1,1,1,1,1,1,1,1,1,1,1,1,1,1,1,1,1,1,1,1,1,1,1,1,1,1,1,1,1,1 |
```


---


 **Insertions and deletions length:**

```
|  |  |
| --- | --- |
| Min | 0 |
| Max | 83 |
| Mean | 0.945 |
| Median | 1 |
| Standard deviation | 1.926 |
| Values | 0,1,2,3,4,5,6,7,8,9,10,11,12,13,14,15,16,17,18,19,20,21,22,23,24,25,26,27,28,29,30,31,32,33,35,36,37,38,39,40,41,42,43,44,45,48,50,51,52,53,55,56,60,61,64,69,71,74,80,83 |
| Count | 24528,45801,2518,1546,517,488,227,186,164,96,40,58,15,28,24,16,8,13,11,7,11,9,5,11,4,4,7,6,3,9,2,3,5,4,2,1,4,2,1,1,3,2,1,4,1,3,1,1,3,4,1,1,2,1,1,2,1,2,2,1 |
```


---


 **Base changes (SNPs)** 

|  |  |  |  |  |
| --- | --- | --- | --- | --- |
|  | **A** | **C** | **G** | **T** |
| **A** | 0 | 21,993 | 57,833 | 31,019 |
| **C** | 21,458 | 0 | 15,748 | 56,960 |
| **G** | 57,362 | 15,670 | 0 | 21,674 |
| **T** | 31,094 | 57,538 | 21,744 | 0 |

---


  **Ts/Tv (transitions / transversions)** 

**Note:** Only SNPs are used for this statistic.  
**Note:** This Ts/Tv ratio is a 'raw' ratio (ratio of observed events).

|  |  |
| --- | --- |
| Transitions | 1,343,660 |
| Transversions | 1,058,969 |
| Ts/Tv ratio | 1.2688 |

**All variants:**

```
Sample ,Total
Transitions ,453559,445596,444505,1343660
Transversions ,357898,351017,350054,1058969
Ts/Tv ,1.267,1.269,1.270,1.269
```

**Only known variants** (i.e. the ones having a non-empty ID field):

```
No results available (empty input?)
```

---


  **Allele frequency** 
  

|  |  |
| --- | --- |
| Min | 16 |
| Max | 100 |
| Mean | 98.152 |
| Median | 100 |
| Standard deviation | 9.555 |
| Values | 16,25,33,50,66,75,83,100 |
| Count | 1613,169,2418,6260,4177,469,7131,463650 |

---


  **Allele Count** 
  

|  |  |
| --- | --- |
| Min | 1 |
| Max | 6 |
| Mean | 5.799 |
| Median | 6 |
| Standard deviation | 0.733 |
| Values | 1,2,3,4,5,6 |
| Count | 1901,5538,6349,20024,7131,444944 |

---


  **Hom/Het per sample** 
  
  
  

```
Sample_names , No_sample_name_found
Reference , 1266, 2582, 2436
Het , 16702, 12429, 10780
Hom , 467497, 460779, 460488
Missing , 422, 10097, 12183
```

---


 **Codon changes**

How to read this table:   
- Rows are reference codons and columns are changed codons. E.g. Row 'AAA' column 'TAA' indicates how many 'AAA' codons have been replaced by 'TAA' codons.  
- Red background colors indicate that more changes happened (heat-map).  
- Diagonals are indicated using grey background color   
- WARNING: This table may include different translation codon tables (e.g. mamalian DNA and mitochondrial DNA).

|  | - | AAA | AAC | AAG | AAT | ACA | ACC | ACG | ACN | ACT | AGA | AGC | AGG | AGT | ATA | ATC | ATG | ATT | CAA | CAC | CAG | CAT | CCA | CCC | CCG | CCT | CGA | CGC | CGG | CGT | CTA | CTC | CTG | CTT | GAA | GAC | GAG | GAT | GCA | GCC | GCG | GCN | GCT | GGA | GGC | GGG | GGT | GTA | GTC | GTG | GTT | TAA | TAC | TAG | TAT | TCA | TCC | TCG | TCT | TGA | TGC | TGG | TGT | TTA | TTC | TTG | TTT |
| --- | --- | --- | --- | --- | --- | --- | --- | --- | --- | --- | --- | --- | --- | --- | --- | --- | --- | --- | --- | --- | --- | --- | --- | --- | --- | --- | --- | --- | --- | --- | --- | --- | --- | --- | --- | --- | --- | --- | --- | --- | --- | --- | --- | --- | --- | --- | --- | --- | --- | --- | --- | --- | --- | --- | --- | --- | --- | --- | --- | --- | --- | --- | --- | --- | --- | --- | --- |
| - |  | 69 | 50 | 108 | 70 | 38 | 12 | 18 |  | 42 | 24 | 44 | 22 | 52 | 16 | 12 | 44 | 40 | 100 | 14 | 52 | 38 | 84 | 16 | 10 | 104 | 2 | 6 | 14 | 32 | 14 | 10 | 10 | 20 | 140 | 54 | 110 | 136 | 14 | 12 | 14 |  | 88 | 116 | 16 | 14 | 60 | 16 | 10 | 30 | 44 | 10 | 26 | 18 | 42 | 74 | 38 | 44 | 106 | 14 | 12 | 6 | 24 | 34 | 40 | 22 | 14 |
| AAA | 175 | 26 | 342 | 2,190 | 374 | 268 |  |  |  | 2 | 708 |  | 4 |  | 226 | 2 | 2 | 2 | 400 |  |  |  |  |  |  |  |  |  |  |  | 2 |  |  |  | 874 |  |  |  |  |  |  |  |  |  |  |  |  |  |  |  |  | 46 |  |  |  |  |  |  |  |  |  |  |  |  |  |  |  |
| AAC | 218 | 382 | 6 | 322 | 2,010 | 8 | 208 | 6 |  | 6 | 2 | 494 |  | 4 | 2 | 104 | 2 | 2 |  | 168 |  |  |  |  |  |  |  |  |  |  |  |  |  |  | 2 | 446 |  |  |  |  |  |  |  |  |  |  |  |  |  |  |  |  | 152 |  |  | 4 |  |  |  |  |  |  |  |  |  |  |  |
| AAG | 162 | 2,108 | 294 | 28 | 392 | 10 |  | 240 |  | 4 | 4 |  | 444 |  | 8 |  | 198 | 2 | 8 |  | 202 |  |  |  |  |  |  |  |  |  |  |  |  |  | 2 |  | 596 |  |  |  |  |  |  |  |  |  |  |  |  |  |  | 4 |  | 46 | 2 |  |  |  |  |  |  |  |  |  |  | 2 |  |
| AAT | 134 | 322 | 2,104 | 370 | 4 |  |  |  |  | 280 |  | 2 |  | 682 | 4 | 2 |  | 262 | 4 |  |  | 204 |  |  |  |  |  |  |  |  |  |  |  |  |  |  | 2 | 884 |  |  |  |  |  |  |  |  |  |  |  |  |  |  |  |  | 162 |  |  |  |  |  |  |  |  |  |  |  |  |
| ACA | 106 | 244 | 8 | 2 | 4 | 4 | 494 | 1,012 |  | 718 | 140 | 4 |  |  | 410 |  |  | 4 |  |  |  |  | 128 |  |  |  |  |  |  |  |  |  |  |  |  |  |  |  | 496 |  |  |  |  |  |  |  |  |  |  |  |  |  | 2 |  |  | 258 |  |  |  |  |  |  |  |  |  |  |  |
| ACC | 42 |  | 202 |  |  | 572 | 2 | 300 |  | 1,086 |  | 212 |  |  |  | 256 |  |  | 2 |  |  |  |  | 80 |  |  |  |  |  |  |  |  |  |  |  |  |  |  |  | 300 |  |  |  |  |  |  |  |  |  |  |  |  |  |  |  |  | 110 |  |  |  |  |  |  |  |  |  |  |
| ACG | 40 |  | 6 | 188 | 2 | 1,130 | 356 | 2 |  | 518 |  | 2 | 74 |  |  | 2 | 356 |  | 2 |  |  |  |  |  | 62 |  |  |  |  |  |  |  |  |  |  |  |  |  |  |  | 218 |  |  |  |  |  |  |  |  |  |  |  |  |  |  |  |  | 94 |  |  |  |  |  |  |  |  |  |
| ACN |  |  |  |  |  |  |  |  |  |  |  |  |  |  |  |  |  |  |  |  |  |  |  |  |  |  |  |  |  |  |  |  |  |  |  |  |  |  |  |  |  |  |  |  |  |  |  |  |  |  |  |  |  |  |  |  |  |  |  |  |  |  |  |  |  |  |  |
| ACT | 90 | 2 |  |  | 270 | 772 | 1,010 | 568 |  | 4 |  | 4 |  | 416 |  |  | 4 | 494 |  |  |  |  |  |  |  | 172 |  |  |  |  |  |  |  |  |  |  |  |  |  |  |  |  | 592 |  |  |  |  |  |  |  |  |  |  |  |  |  | 2 |  | 298 |  |  |  |  |  |  |  |  |
| AGA | 72 | 732 | 2 | 10 |  | 184 |  |  |  |  | 6 | 104 | 896 | 144 | 172 |  |  |  |  |  |  |  |  |  |  |  | 474 |  |  |  |  |  |  |  |  |  |  |  |  |  |  |  |  | 412 |  |  |  |  |  |  |  |  |  | 4 |  |  |  |  | 2 | 28 |  |  |  |  |  |  |  |
| AGC | 78 | 2 | 528 | 10 | 6 |  | 130 | 2 |  | 2 | 136 | 2 | 68 | 992 |  | 78 |  |  | 2 |  |  |  |  |  |  |  |  | 90 |  |  |  |  |  |  |  |  |  |  |  |  |  |  | 6 |  | 198 |  |  |  |  |  |  |  |  |  |  |  |  |  |  |  | 74 |  |  |  |  |  |  |
| AGG | 32 | 4 | 2 | 482 |  |  |  | 86 |  |  | 1,012 | 76 |  | 114 | 2 | 2 | 106 |  |  |  |  |  |  |  |  |  |  |  | 284 |  |  |  |  |  |  |  |  |  |  |  |  |  |  |  |  | 142 |  |  |  |  |  |  |  |  |  |  |  |  |  |  |  | 50 |  | 2 |  |  |  |
| AGT | 88 |  | 6 | 10 | 696 | 2 |  |  |  | 398 | 190 | 1,104 | 130 | 4 | 2 |  | 2 | 146 |  |  |  |  |  |  |  |  |  |  |  | 100 |  |  |  |  |  |  | 2 |  |  |  |  |  |  |  |  |  | 480 |  |  |  |  |  |  |  |  |  |  |  |  |  |  |  | 124 |  |  |  |  |
| ATA | 70 | 192 | 2 | 2 | 4 | 360 |  |  |  | 4 | 172 |  | 2 | 2 | 2 | 724 | 398 | 836 |  |  |  |  |  |  |  |  |  |  |  |  | 176 |  |  |  |  |  |  | 6 |  |  |  |  |  |  |  |  |  | 516 |  |  |  |  |  |  |  |  |  |  |  |  |  |  |  | 222 |  |  | 2 |
| ATC | 76 | 2 | 94 |  | 14 | 2 | 288 | 2 |  |  |  | 74 |  |  | 698 | 14 | 162 | 1,794 |  |  |  |  |  |  |  |  |  |  |  |  |  | 230 |  |  |  |  |  |  |  |  |  |  |  |  |  |  |  |  | 576 |  |  | 2 |  |  |  |  |  |  |  |  |  |  |  |  | 116 |  |  |
| ATG | 232 | 14 |  | 262 | 55 |  |  | 460 |  |  | 7 | 2 | 90 | 4 | 510 | 114 | 2 | 267 | 4 |  |  | 2 |  |  |  |  |  |  |  |  |  |  | 226 |  |  |  |  | 3 |  |  |  |  |  |  |  |  |  |  |  | 460 |  |  |  |  | 2 |  |  | 2 |  |  |  |  |  |  |  | 344 |  |
| ATT | 106 | 2 |  |  | 216 | 4 |  |  |  | 442 |  |  |  | 160 | 768 | 1,746 | 232 | 6 |  |  |  |  |  |  |  |  |  |  |  |  |  |  |  | 306 |  |  | 4 | 4 |  |  |  |  |  |  |  |  |  |  |  |  | 1,060 |  |  |  |  |  |  |  |  |  |  |  |  |  |  |  | 232 |
| CAA | 158 | 384 |  |  |  |  |  |  |  |  |  |  |  |  |  |  |  |  | 26 | 222 | 1,234 | 210 | 192 |  |  |  | 400 |  |  |  | 154 |  |  |  | 418 |  |  |  | 2 |  |  |  |  |  |  |  |  |  |  |  |  | 68 |  |  |  | 2 |  |  | 2 |  |  |  |  |  |  |  |  |
| CAC | 68 |  | 144 |  |  |  |  |  |  |  |  |  |  |  |  |  |  |  | 160 | 6 | 170 | 920 | 4 | 52 |  | 2 | 2 | 134 |  |  |  | 82 |  |  |  | 108 |  |  |  |  |  |  |  |  |  |  |  |  |  |  |  |  | 198 |  |  |  |  |  |  |  |  |  |  |  |  |  |  |
| CAG | 104 |  |  | 248 |  | 4 |  |  |  |  |  | 2 |  |  |  |  | 2 |  | 1,232 | 166 | 6 | 218 | 2 |  | 132 | 8 |  |  | 204 |  |  | 2 | 130 |  |  |  | 232 |  | 2 |  |  |  |  |  |  |  |  |  |  |  |  |  |  | 86 |  | 2 |  |  |  |  |  |  |  |  |  |  |  |
| CAT | 86 |  |  |  | 196 | 2 |  |  |  | 4 |  |  |  |  | 2 |  |  |  | 248 | 932 | 184 | 4 | 2 |  |  | 210 |  |  |  | 286 |  |  |  | 110 |  |  |  | 222 |  |  |  |  |  |  |  |  |  |  |  |  |  |  | 2 |  | 264 | 8 |  |  |  |  |  |  |  |  |  |  |  |
| CCA | 138 |  |  |  |  | 160 |  | 2 |  |  |  |  |  |  |  |  |  |  | 188 |  |  |  | 14 | 432 | 1,234 | 700 | 80 |  |  |  | 192 |  | 2 |  |  |  |  |  | 128 |  |  |  |  |  |  |  |  |  |  |  |  |  |  |  |  | 412 | 2 |  |  |  |  |  |  |  |  |  |  |
| CCC | 42 |  | 2 |  |  |  | 82 |  |  |  |  |  |  |  |  |  |  |  |  | 48 |  |  | 316 |  | 282 | 640 |  | 22 |  |  |  | 78 |  |  |  |  |  |  |  | 48 |  |  |  |  |  |  |  |  |  |  |  |  |  |  |  |  | 244 |  |  |  |  |  |  |  |  |  |  |
| CCG | 54 |  |  |  |  |  |  | 60 |  |  |  |  |  |  |  |  |  |  |  |  | 110 |  | 1,372 | 354 | 2 | 590 |  |  | 64 |  |  |  | 236 | 8 |  |  |  |  |  |  | 68 |  |  |  |  |  |  |  |  |  |  |  |  |  |  |  | 2 | 198 |  |  |  |  |  |  |  |  |  |
| CCT | 106 |  |  |  |  |  |  |  |  | 116 |  |  |  |  | 2 |  |  |  | 2 |  | 2 | 180 | 734 | 662 | 532 | 6 |  | 2 |  | 118 | 2 | 4 |  | 324 |  |  |  |  |  |  |  |  | 120 |  | 2 |  |  |  |  |  |  |  |  |  |  |  | 4 |  | 464 |  |  |  |  |  |  |  |  |
| CGA | 26 |  |  |  |  |  |  |  |  |  | 448 |  |  |  |  |  |  |  | 364 |  |  |  | 76 |  |  | 2 |  | 96 | 472 | 200 | 60 |  | 2 |  |  |  |  |  |  |  |  |  |  | 102 |  |  |  |  |  |  |  |  |  |  |  |  |  | 2 |  | 40 | 2 |  |  |  |  |  |  |
| CGC | 16 |  |  |  |  |  |  |  |  |  |  | 70 |  |  |  |  |  |  |  | 152 | 2 |  |  | 24 |  |  | 132 |  | 126 | 442 |  | 50 | 2 |  |  |  |  |  |  |  |  |  |  |  | 26 |  |  |  |  |  |  |  |  |  |  |  |  | 2 |  |  | 84 |  |  |  |  |  |  |
| CGG | 18 |  |  |  |  |  |  |  |  |  |  |  | 278 |  |  |  |  |  |  |  | 212 |  |  |  | 44 | 2 | 422 | 100 |  | 226 |  |  | 50 |  |  |  |  |  |  |  | 2 |  |  |  |  | 48 |  |  |  |  |  |  |  |  |  |  |  |  |  |  |  | 116 |  |  |  |  |  |
| CGT | 40 |  |  |  |  |  |  |  |  |  |  |  |  | 116 |  |  |  |  | 4 |  |  | 280 |  |  | 2 | 82 | 282 | 522 | 240 | 4 |  |  |  | 140 |  |  |  |  |  |  |  |  |  |  |  |  | 108 |  |  |  |  |  |  |  |  |  |  |  |  |  |  |  | 132 |  |  |  |  |
| CTA | 28 |  |  |  |  |  |  |  |  | 2 |  |  |  |  | 134 |  |  |  | 134 |  |  |  | 220 |  | 2 | 4 | 60 |  |  |  | 2 | 476 | 854 | 638 |  |  |  |  |  |  |  |  |  |  |  |  |  | 162 |  |  |  |  |  |  |  |  |  |  | 2 |  |  |  |  | 848 |  |  |  |
| CTC | 58 |  |  |  |  |  |  |  |  | 2 |  |  |  |  |  | 246 |  |  | 2 | 72 |  |  |  | 116 |  |  |  | 50 | 2 | 4 | 542 | 6 | 388 | 1,630 |  |  |  |  |  |  |  |  |  |  |  |  |  |  | 178 |  | 2 |  |  |  |  |  |  |  | 2 |  |  |  |  |  | 280 |  |  |
| CTG | 44 |  |  |  |  |  |  |  |  |  |  |  |  |  |  |  | 184 |  | 2 |  | 94 | 4 |  |  | 212 |  |  |  | 28 |  | 802 | 420 |  | 522 |  |  |  |  |  |  |  |  |  |  |  |  |  |  |  | 196 |  |  |  |  |  |  |  |  | 2 |  |  |  |  |  |  | 876 |  |
| CTT | 72 |  |  |  |  |  |  |  |  | 2 |  |  |  |  |  | 2 |  | 392 |  |  |  | 124 |  |  |  | 286 | 2 |  |  | 116 | 684 | 1,512 | 514 | 6 |  |  |  |  |  |  |  |  | 4 |  |  |  |  |  |  |  | 298 |  |  |  |  |  |  |  | 8 |  |  |  |  |  |  |  | 440 |
| GAA | 354 | 832 |  |  |  |  |  |  |  |  |  |  |  | 2 |  |  |  |  | 382 |  |  |  |  |  |  |  |  |  | 2 |  |  |  |  |  | 32 | 508 | 1,924 | 576 | 218 | 6 |  |  |  | 528 | 2 | 2 | 2 | 142 | 4 | 4 | 6 | 62 |  |  |  |  |  |  |  | 2 |  |  |  |  |  |  |  |
| GAC | 78 |  | 410 |  |  |  |  |  |  |  | 2 |  |  |  |  |  | 2 |  |  | 88 |  |  |  |  |  |  |  |  |  |  |  |  |  |  | 516 | 8 | 382 | 2,116 |  | 70 |  |  |  | 8 | 250 |  | 2 | 2 | 38 |  |  |  | 122 |  |  |  |  |  | 2 | 4 |  |  |  |  |  |  |  |
| GAG | 148 |  |  | 598 |  |  |  |  |  |  | 2 |  |  |  |  |  |  |  |  |  | 248 |  |  |  |  |  |  |  |  |  |  |  |  |  | 2,248 | 462 | 16 | 582 |  |  | 150 |  | 2 | 12 |  | 248 | 2 | 2 |  | 196 |  |  |  | 32 |  |  |  |  |  | 8 |  |  |  |  |  |  |  |
| GAT | 304 |  |  |  | 810 |  |  |  |  |  |  |  |  |  |  |  | 2 |  |  |  |  | 196 |  |  |  |  |  |  |  |  |  |  |  |  | 590 | 2,090 | 566 | 16 | 4 | 2 |  |  | 214 | 6 | 2 | 2 | 482 | 4 | 2 |  | 146 |  |  |  | 228 |  |  |  | 2 | 8 |  |  |  |  |  |  |  |
| GCA | 54 |  |  |  |  | 532 |  |  |  |  |  |  |  |  |  |  |  |  |  |  |  |  | 138 |  |  | 2 |  |  |  |  |  |  |  |  | 220 | 2 |  |  | 8 | 492 | 1,198 |  | 898 | 178 | 6 | 2 |  | 324 |  |  |  |  |  | 2 |  | 236 |  |  |  |  |  |  |  |  |  |  |  |
| GCC | 56 |  |  | 2 |  |  | 318 |  |  |  |  |  |  |  |  |  |  |  |  |  |  |  |  | 70 |  |  |  |  |  |  |  |  |  |  | 2 | 52 | 2 |  | 452 | 2 | 388 |  | 1,236 | 4 | 144 |  |  |  | 264 |  |  |  |  |  |  |  | 170 |  |  |  |  |  |  |  |  | 2 |  |
| GCG | 28 |  |  |  |  |  |  | 238 |  |  |  |  |  |  |  |  |  |  |  |  |  |  |  |  | 52 |  |  |  |  |  |  |  |  | 2 |  |  | 108 |  | 1,226 | 472 | 4 |  | 664 |  | 6 | 108 |  |  |  | 386 | 2 |  |  |  |  |  |  | 116 |  |  |  |  |  |  |  |  |  |
| GCN |  |  |  |  |  |  |  |  | 2 |  |  |  |  |  |  |  |  |  |  |  |  |  |  |  |  |  |  |  |  |  |  |  |  |  |  |  |  |  |  |  |  |  |  |  |  |  |  |  |  |  |  |  |  |  |  |  |  |  |  |  |  |  |  |  |  |  |  |
| GCT | 88 |  |  |  |  |  |  |  |  | 614 |  |  |  |  |  |  |  |  |  |  |  |  |  |  |  | 178 |  |  |  |  |  |  |  |  | 14 |  |  | 192 | 776 | 1,136 | 726 |  | 6 | 2 | 4 |  | 254 | 2 | 4 | 2 | 504 |  |  |  |  |  |  |  | 396 |  | 2 |  |  |  |  |  |  |
| GGA | 215 |  |  |  |  |  |  |  |  |  | 350 |  | 2 |  |  |  |  |  | 6 |  |  |  |  |  |  |  | 86 |  |  |  |  |  |  |  | 446 |  | 2 | 2 | 174 |  |  |  | 10 | 16 | 526 | 1,268 | 650 | 98 |  |  |  |  |  |  |  |  |  |  | 6 | 24 |  |  | 2 |  |  |  |  |
| GGC | 36 |  |  |  |  |  |  | 2 |  |  |  | 220 |  |  |  |  |  |  |  |  |  |  |  |  |  |  |  | 42 |  |  |  |  |  |  | 2 | 186 |  |  |  | 126 |  |  |  | 538 | 2 | 300 | 1,018 |  | 68 |  |  |  |  |  |  |  |  |  |  |  | 62 |  |  |  |  |  |  |
| GGG | 38 |  |  |  |  |  | 2 |  |  |  |  |  | 178 |  |  |  |  |  |  |  |  |  |  |  |  |  |  |  | 62 |  |  |  |  |  |  |  | 284 |  |  | 4 | 92 |  |  | 1,254 | 310 | 8 | 448 |  |  | 62 | 10 |  |  |  |  |  |  |  | 6 |  |  | 48 |  |  |  |  |  |
| GGT | 138 |  |  |  |  |  |  |  |  |  |  |  |  | 534 |  |  |  |  |  |  |  |  |  |  |  |  |  |  |  | 86 |  |  |  |  | 2 |  | 2 | 510 | 4 |  | 2 |  | 298 | 716 | 1,156 | 504 | 12 |  |  |  | 190 |  |  |  |  |  |  |  |  |  |  | 2 | 136 |  |  |  |  |
| GTA | 38 |  |  |  |  |  |  |  |  |  |  |  |  |  | 540 |  |  |  |  |  |  |  |  |  |  |  |  |  |  |  | 158 |  |  |  | 158 |  | 2 | 2 | 330 |  |  |  |  | 108 |  |  | 4 | 6 | 360 | 1,068 | 642 |  |  | 2 |  |  |  |  |  |  |  |  | 2 | 170 |  |  |  |
| GTC | 58 |  |  |  |  |  |  |  |  |  |  |  |  |  |  | 556 |  |  |  |  |  |  |  |  |  |  |  |  |  |  |  | 168 |  |  | 2 | 52 |  |  |  | 264 |  |  |  |  | 32 |  | 2 | 350 | 4 | 510 | 1,342 |  |  |  |  |  |  |  |  |  |  |  |  |  | 150 |  |  |
| GTG | 48 |  |  |  |  |  |  |  |  |  |  |  |  |  |  |  | 520 |  |  |  |  |  |  |  |  |  |  |  | 2 | 4 |  |  | 140 |  | 2 | 2 | 168 | 4 |  |  | 352 |  | 6 | 2 |  | 90 | 2 | 1,060 | 492 | 2 | 900 |  |  |  |  |  |  |  | 2 |  |  |  | 2 |  |  | 218 |  |
| GTT | 102 |  |  |  |  |  |  |  |  |  |  |  |  |  |  | 6 | 2 | 1,052 |  |  |  |  |  |  |  |  |  |  |  | 2 |  |  |  | 338 | 8 |  |  | 170 |  |  |  |  | 594 | 2 |  |  | 224 | 638 | 1,416 | 794 | 10 |  |  |  |  |  |  |  |  |  |  |  |  |  |  |  | 280 |
| TAA | 12 | 8 |  |  |  |  |  |  |  |  |  |  |  |  |  |  |  |  | 18 |  |  |  |  |  |  |  |  |  |  |  | 2 |  |  |  | 10 |  |  |  |  |  |  |  |  |  |  |  |  |  |  |  |  | 6 | 10 | 30 | 4 | 8 |  |  |  | 38 |  |  |  | 6 |  |  |  |
| TAC | 70 |  | 112 |  | 4 |  |  |  |  |  |  |  |  |  |  |  |  | 2 |  | 174 |  |  |  |  |  |  |  |  |  |  |  |  |  |  |  | 96 |  |  |  |  |  |  |  |  |  |  |  |  |  |  |  | 20 | 2 | 20 | 1,348 | 2 | 56 |  | 2 |  | 132 |  | 2 | 2 | 206 |  | 2 |
| TAG | 17 |  |  | 6 |  |  |  |  |  |  |  |  |  |  | 2 |  |  |  |  |  | 32 |  |  |  |  |  |  |  |  |  |  |  |  |  |  |  | 8 |  |  |  |  |  |  |  |  |  |  |  |  |  |  | 60 | 8 |  | 6 |  |  | 6 |  | 2 |  | 12 |  |  |  | 10 |  |
| TAT | 110 |  |  |  | 176 |  |  |  |  |  |  |  |  |  |  |  |  |  |  |  |  | 228 |  |  |  |  |  |  |  |  |  |  |  |  |  |  |  | 168 |  |  |  |  |  |  |  |  |  | 4 |  |  |  | 24 | 1,482 | 12 | 8 |  |  |  | 214 | 4 |  |  | 222 | 8 |  | 2 | 254 |
| TCA | 172 |  |  |  |  | 272 |  |  |  | 2 |  |  |  |  |  | 2 | 2 |  |  |  |  |  | 432 |  |  |  |  |  |  |  |  |  |  |  |  |  |  | 2 | 250 |  | 2 |  |  |  |  |  |  |  |  |  |  | 20 | 4 |  |  | 14 | 534 | 1,360 | 800 | 20 |  |  | 2 | 374 | 4 | 2 | 2 |
| TCC | 74 |  |  |  | 2 |  | 162 |  |  |  |  |  |  |  |  |  |  |  |  |  |  |  |  | 148 |  |  |  |  |  |  |  | 6 |  |  |  |  |  |  |  | 184 |  |  |  |  |  |  |  |  |  |  |  |  | 74 |  |  | 452 | 2 | 480 | 1,366 |  | 60 |  | 2 |  | 204 |  | 2 |
| TCG | 70 |  |  |  | 2 |  |  | 130 |  |  |  |  |  |  |  |  |  |  |  |  |  |  |  |  | 242 |  |  |  |  |  |  |  |  |  |  | 2 |  |  |  |  | 116 |  |  |  |  |  |  |  |  |  |  |  | 2 | 18 |  | 1,398 | 474 |  | 856 |  | 4 | 66 |  |  | 4 | 366 |  |
| TCT | 234 |  |  |  |  |  |  |  |  | 322 |  |  |  |  |  | 10 |  |  |  |  |  |  |  |  |  | 398 |  |  |  |  |  |  |  |  |  |  |  | 2 | 2 |  |  |  | 426 |  |  |  |  |  | 2 |  |  |  | 4 |  | 202 | 802 | 1,276 | 836 | 20 |  |  |  | 246 |  | 10 |  | 386 |
| TGA | 32 |  |  |  |  |  |  |  |  |  | 22 |  |  |  |  |  |  |  |  |  |  |  |  |  |  |  | 18 |  |  |  |  |  |  |  |  |  |  |  |  |  |  |  |  | 6 |  |  |  |  |  |  |  | 76 |  | 2 |  | 10 |  |  | 2 |  | 10 | 14 | 2 |  |  | 4 |  |
| TGC | 22 |  | 2 |  |  |  |  |  |  |  |  | 76 |  |  |  |  |  |  |  |  |  |  |  |  |  |  |  | 136 |  |  |  |  |  |  |  |  |  |  |  |  |  |  |  |  | 74 |  |  | 2 |  |  |  |  | 98 |  |  |  | 62 |  |  | 14 |  | 46 | 804 |  | 42 | 2 |  |
| TGG | 38 |  |  |  |  |  |  |  |  |  |  |  | 50 |  |  |  |  |  |  |  |  |  |  |  |  | 6 |  |  | 104 |  |  |  |  |  | 2 |  |  |  |  |  |  |  |  |  |  | 48 |  |  |  |  |  |  |  | 64 |  |  |  | 62 |  | 56 | 34 | 2 | 60 |  |  | 100 |  |
| TGT | 58 |  |  |  |  |  |  |  |  |  |  |  | 2 | 152 |  |  | 2 |  |  |  |  |  |  |  |  |  |  |  |  | 190 |  |  |  |  |  |  |  |  |  |  |  |  |  |  |  |  | 132 |  |  |  |  |  | 2 | 4 | 260 | 2 |  |  | 160 | 10 | 846 | 58 | 2 |  |  | 10 | 126 |
| TTA | 60 |  |  |  |  |  |  |  |  |  |  |  |  |  | 218 |  |  | 2 |  |  |  |  |  |  |  |  |  |  |  |  | 812 |  |  |  | 2 |  |  |  |  |  |  |  |  |  |  |  |  | 106 |  |  | 2 | 20 |  |  |  | 324 | 2 |  | 4 | 24 |  |  |  | 4 | 190 | 1,386 | 234 |
| TTC | 102 |  | 2 | 2 |  |  |  |  |  |  |  |  |  |  |  | 114 |  |  |  |  |  |  |  |  |  |  |  |  |  |  |  | 292 |  |  |  |  |  |  |  |  |  |  | 2 |  |  |  |  |  | 154 |  | 4 |  | 200 |  |  | 2 | 210 |  | 10 |  | 50 | 2 |  | 200 | 16 | 200 | 2,090 |
| TTG | 72 |  |  |  |  |  |  |  |  |  |  |  |  |  |  |  | 290 |  |  |  |  |  |  |  |  |  |  |  |  |  |  |  | 1,162 |  |  |  |  |  |  |  |  |  |  |  |  |  |  |  |  | 210 |  |  |  | 18 | 2 | 4 | 4 | 348 | 4 | 2 |  | 102 |  | 1,398 | 220 | 4 | 296 |
| TTT | 120 |  |  |  |  |  |  |  |  |  |  |  |  |  |  |  |  | 208 |  |  |  |  |  |  |  |  |  |  |  |  |  |  |  | 398 |  |  |  |  |  |  |  |  |  |  |  |  | 2 |  |  |  | 228 | 2 |  |  | 258 |  |  |  | 426 | 4 |  |  | 124 | 184 | 2,012 | 244 | 14 |


---


 **Amino acid changes**

How to read this table:   
- Rows are reference amino acids and columns are changed amino acids. E.g. Row 'A' column 'E' indicates how many 'A' amino acids have been replaced by 'E' amino acids.  
- Red background colors indicate that more changes happened (heat-map).  
- Diagonals are indicated using grey background color   
- WARNING: This table may include different translation codon tables (e.g. mamalian DNA and mitochondrial DNA).

|  | \* | - | ? | A | C | D | E | F | G | H | I | K | L | M | N | P | Q | R | S | T | V | W | Y |
| --- | --- | --- | --- | --- | --- | --- | --- | --- | --- | --- | --- | --- | --- | --- | --- | --- | --- | --- | --- | --- | --- | --- | --- |
| \* | 214 | 60 | 1 |  | 12 |  | 18 |  | 6 |  | 2 | 14 | 22 |  |  |  | 50 | 40 | 26 |  |  | 26 | 28 |
| - | 42 |  | 1,512 | 128 | 36 | 190 | 250 | 54 | 206 | 52 | 68 | 177 | 110 | 44 | 120 | 214 | 152 | 100 | 358 | 110 | 100 | 6 | 68 |
| ? |  |  | 2 |  |  |  |  |  |  |  |  |  |  |  |  |  |  |  |  |  |  |  |  |
| A | 2 | 226 |  | 9,684 | 2 | 246 | 346 |  | 708 |  |  | 2 | 4 |  |  | 440 |  |  | 918 | 1,702 | 1,488 |  |  |
| C | 28 | 80 |  |  | 1,652 |  |  | 168 | 206 |  |  |  | 12 | 2 | 2 |  |  | 328 | 452 |  | 2 | 104 | 360 |
| D | 12 | 382 |  | 290 |  | 4,230 | 2,054 |  | 752 | 284 |  |  |  | 4 | 1,220 |  |  | 2 | 4 |  | 192 |  | 350 |
| E | 104 | 502 |  | 376 |  | 2,128 | 4,220 |  | 796 |  |  | 1,430 |  |  |  |  | 630 | 4 | 2 |  | 354 |  |  |
| F | 6 | 222 |  | 2 | 174 |  |  | 4,132 | 2 |  | 322 | 2 | 1,518 |  | 2 |  |  |  | 648 |  | 386 | 2 | 458 |
| G | 24 | 427 |  | 710 | 200 | 698 | 738 |  | 8,726 |  |  |  |  |  |  |  | 6 | 806 | 766 | 4 | 428 | 50 |  |
| H |  | 154 |  |  |  | 330 |  |  |  | 1,862 | 2 |  | 192 |  | 340 | 270 | 762 | 422 | 8 | 6 |  |  | 464 |
| I | 2 | 252 |  |  |  | 10 | 4 | 350 |  |  | 6,588 | 198 | 934 | 792 | 330 |  |  | 174 | 236 | 1,102 | 2,152 |  |  |
| K | 96 | 336 | 1 |  |  |  | 1,472 |  |  |  | 240 | 4,352 | 4 | 200 | 1,402 |  | 610 | 1,160 |  | 524 |  |  | 2 |
| L | 64 | 334 |  | 4 |  |  | 2 | 1,660 |  | 200 | 994 |  | 15,486 | 474 |  | 840 | 232 | 262 | 704 | 6 | 1,154 | 102 | 2 |
| M |  | 232 |  |  |  | 3 |  |  |  | 2 | 891 | 276 | 570 | 2 | 55 |  | 4 | 97 | 8 | 460 | 460 |  | 2 |
| N |  | 352 |  |  |  | 1,330 | 4 |  |  | 372 | 376 | 1,396 |  | 2 | 4,124 |  | 4 | 2 | 1,186 | 508 |  |  | 314 |
| P |  | 340 |  | 364 |  |  |  |  | 2 | 228 | 2 |  | 846 |  | 2 | 7,870 | 302 | 286 | 1,326 | 420 |  |  |  |
| Q | 154 | 262 |  | 4 |  |  | 650 |  |  | 816 |  | 632 | 286 | 2 |  | 334 | 2,498 | 604 | 8 | 4 |  |  |  |
| R | 72 | 204 |  | 2 | 218 |  |  |  | 838 | 432 | 176 | 1,228 | 306 | 106 | 4 | 232 | 582 | 6,662 | 630 | 270 |  | 166 |  |
| S | 58 | 716 |  | 986 | 512 | 6 | 2 | 612 | 678 |  | 238 | 22 | 748 | 4 | 1,240 | 1,220 | 2 | 714 | 12,772 | 1,422 | 2 | 66 | 286 |
| T |  | 278 |  | 1,606 |  |  |  |  |  |  | 1,166 | 436 |  | 360 | 492 | 442 | 4 | 214 | 1,400 | 8,548 |  |  | 2 |
| V | 2 | 246 |  | 1,546 | 4 | 230 | 340 | 430 | 466 |  | 2,154 |  | 1,192 | 522 |  |  |  | 8 | 2 |  | 9,594 |  |  |
| W | 120 | 38 |  |  | 94 |  | 2 |  | 48 |  |  |  | 100 |  |  | 6 |  | 154 | 62 |  |  | 2 |  |
| Y | 80 | 180 |  |  | 356 | 264 |  | 462 |  | 402 | 2 |  | 12 |  | 292 |  |  |  | 274 |  | 4 |  | 2,840 |


---


 **Variants by chromosome**

```
		  

		1, Position,0,1000000,2000000,3000000,4000000,5000000,6000000,7000000,8000000,9000000,10000000,11000000,12000000,13000000,14000000,15000000,16000000,17000000,18000000,19000000,20000000,21000000,22000000,23000000,24000000,25000000,26000000,27000000,28000000,29000000,30000000
1,Count,3003,2898,3098,2872,4304,3315,2344,4068,4621,3846,5061,6660,5439,7865,3756,4435,8022,6138,4397,4878,3147,6107,3960,2993,3308,2710,4438,3299,2737,3174,1543

	
```

```
		  

		2, Position,0,100000,200000,300000,400000,500000,600000,700000,800000,900000,1000000,1100000,1200000,1300000,1400000,1500000,1600000,1700000,1800000,1900000,2000000,2100000,2200000,2300000,2400000,2500000,2600000,2700000,2800000,2900000,3000000,3100000,3200000,3300000,3400000,3500000,3600000,3700000,3800000,3900000,4000000,4100000,4200000,4300000,4400000,4500000,4600000,4700000,4800000,4900000,5000000,5100000,5200000,5300000,5400000,5500000,5600000,5700000,5800000,5900000,6000000,6100000,6200000,6300000,6400000,6500000,6600000,6700000,6800000,6900000,7000000,7100000,7200000,7300000,7400000,7500000,7600000,7700000,7800000,7900000,8000000,8100000,8200000,8300000,8400000,8500000,8600000,8700000,8800000,8900000,9000000,9100000,9200000,9300000,9400000,9500000,9600000,9700000,9800000,9900000,10000000,10100000,10200000,10300000,10400000,10500000,10600000,10700000,10800000,10900000,11000000,11100000,11200000,11300000,11400000,11500000,11600000,11700000,11800000,11900000,12000000,12100000,12200000,12300000,12400000,12500000,12600000,12700000,12800000,12900000,13000000,13100000,13200000,13300000,13400000,13500000,13600000,13700000,13800000,13900000,14000000,14100000,14200000,14300000,14400000,14500000,14600000,14700000,14800000,14900000,15000000,15100000,15200000,15300000,15400000,15500000,15600000,15700000,15800000,15900000,16000000,16100000,16200000,16300000,16400000,16500000,16600000,16700000,16800000,16900000,17000000,17100000,17200000,17300000,17400000,17500000,17600000,17700000,17800000,17900000,18000000,18100000,18200000,18300000,18400000,18500000,18600000,18700000,18800000,18900000,19000000,19100000,19200000,19300000,19400000,19500000,19600000
2,Count,113,555,322,514,37,15,361,18,3,8,524,527,597,686,665,813,689,190,456,821,664,992,764,660,367,387,221,541,892,796,749,356,30,27,23,681,407,825,1116,368,746,1071,895,885,825,949,1039,1224,926,755,832,553,953,1187,716,591,925,151,625,579,700,271,500,878,791,738,624,734,635,62,511,493,692,485,692,792,711,522,406,312,421,514,574,623,606,607,515,19,460,364,659,470,479,453,260,428,401,587,453,592,523,557,643,479,503,669,640,562,634,180,385,257,439,191,335,177,45,4,324,5,47,435,328,636,763,575,485,161,515,223,498,256,226,319,306,574,345,320,354,418,332,240,22,8,11,125,309,435,399,303,455,223,279,560,267,169,184,125,106,444,364,309,412,110,162,189,189,313,467,287,177,145,444,174,222,462,575,166,316,449,318,338,441,407,311,244,257,266,177,6,66,164,118,199,238,18,236

	
```

```
		  

		3, Position,0,100000,200000,300000,400000,500000,600000,700000,800000,900000,1000000,1100000,1200000,1300000,1400000,1500000,1600000,1700000,1800000,1900000,2000000,2100000,2200000,2300000,2400000,2500000,2600000,2700000,2800000,2900000,3000000,3100000,3200000,3300000,3400000,3500000,3600000,3700000,3800000,3900000,4000000,4100000,4200000,4300000,4400000,4500000,4600000,4700000,4800000,4900000,5000000,5100000,5200000,5300000,5400000,5500000,5600000,5700000,5800000,5900000,6000000,6100000,6200000,6300000,6400000,6500000,6600000,6700000,6800000,6900000,7000000,7100000,7200000,7300000,7400000,7500000,7600000,7700000,7800000,7900000,8000000,8100000,8200000,8300000,8400000,8500000,8600000,8700000,8800000,8900000,9000000,9100000,9200000,9300000,9400000,9500000,9600000,9700000,9800000,9900000,10000000,10100000,10200000,10300000,10400000,10500000,10600000,10700000,10800000,10900000,11000000,11100000,11200000,11300000,11400000,11500000,11600000,11700000,11800000,11900000,12000000,12100000,12200000,12300000,12400000,12500000,12600000,12700000,12800000,12900000,13000000,13100000,13200000,13300000,13400000,13500000,13600000,13700000,13800000,13900000,14000000,14100000,14200000,14300000,14400000,14500000,14600000,14700000,14800000,14900000,15000000,15100000,15200000,15300000,15400000,15500000,15600000,15700000,15800000,15900000,16000000,16100000,16200000,16300000,16400000,16500000,16600000,16700000,16800000,16900000,17000000,17100000,17200000,17300000,17400000,17500000,17600000,17700000,17800000,17900000,18000000,18100000,18200000,18300000,18400000,18500000,18600000,18700000,18800000,18900000,19000000,19100000,19200000,19300000,19400000,19500000,19600000,19700000,19800000,19900000,20000000,20100000,20200000,20300000,20400000,20500000,20600000,20700000,20800000,20900000,21000000,21100000,21200000,21300000,21400000,21500000,21600000,21700000,21800000,21900000,22000000,22100000,22200000,22300000,22400000,22500000,22600000,22700000,22800000,22900000,23000000,23100000,23200000,23300000,23400000
3,Count,327,187,119,321,167,263,9,10,8,215,451,422,296,214,267,291,112,175,389,202,252,290,371,137,282,159,204,456,222,227,137,559,304,322,258,592,328,330,234,157,220,275,319,335,225,447,213,307,346,257,233,221,325,511,582,310,411,240,299,352,286,252,436,258,266,395,614,381,366,87,40,135,173,414,339,296,333,331,352,368,438,619,678,378,508,156,9,3,8,4,4,3,4,357,478,332,465,405,447,369,356,576,581,588,617,593,710,575,671,592,502,825,806,898,970,676,510,753,866,634,973,1171,889,1030,811,1100,833,33,48,290,84,65,143,614,1245,848,98,0,101,260,557,646,820,241,47,132,229,975,783,1064,997,916,852,1035,277,395,927,350,15,13,75,687,641,784,851,755,670,805,704,584,481,788,480,530,543,537,562,273,491,614,573,382,579,482,380,440,148,701,468,468,196,425,434,239,534,451,671,358,619,199,10,7,292,356,376,387,472,526,380,379,464,105,87,278,411,446,279,169,299,147,404,408,23,15,209,255,333,435,232,305,398,328,359,374,139

	
```

```
		  

		4, Position,0,100000,200000,300000,400000,500000,600000,700000,800000,900000,1000000,1100000,1200000,1300000,1400000,1500000,1600000,1700000,1800000,1900000,2000000,2100000,2200000,2300000,2400000,2500000,2600000,2700000,2800000,2900000,3000000,3100000,3200000,3300000,3400000,3500000,3600000,3700000,3800000,3900000,4000000,4100000,4200000,4300000,4400000,4500000,4600000,4700000,4800000,4900000,5000000,5100000,5200000,5300000,5400000,5500000,5600000,5700000,5800000,5900000,6000000,6100000,6200000,6300000,6400000,6500000,6600000,6700000,6800000,6900000,7000000,7100000,7200000,7300000,7400000,7500000,7600000,7700000,7800000,7900000,8000000,8100000,8200000,8300000,8400000,8500000,8600000,8700000,8800000,8900000,9000000,9100000,9200000,9300000,9400000,9500000,9600000,9700000,9800000,9900000,10000000,10100000,10200000,10300000,10400000,10500000,10600000,10700000,10800000,10900000,11000000,11100000,11200000,11300000,11400000,11500000,11600000,11700000,11800000,11900000,12000000,12100000,12200000,12300000,12400000,12500000,12600000,12700000,12800000,12900000,13000000,13100000,13200000,13300000,13400000,13500000,13600000,13700000,13800000,13900000,14000000,14100000,14200000,14300000,14400000,14500000,14600000,14700000,14800000,14900000,15000000,15100000,15200000,15300000,15400000,15500000,15600000,15700000,15800000,15900000,16000000,16100000,16200000,16300000,16400000,16500000,16600000,16700000,16800000,16900000,17000000,17100000,17200000,17300000,17400000,17500000,17600000,17700000,17800000,17900000,18000000,18100000,18200000,18300000,18400000,18500000
4,Count,10,5,2,7,8,21,191,674,498,560,258,6,13,490,491,621,120,383,982,941,891,600,649,979,672,776,806,430,1044,454,570,351,218,451,398,432,371,357,311,250,701,837,360,209,54,241,436,346,823,978,554,644,870,607,656,504,774,715,330,446,762,888,656,745,752,834,591,639,654,392,758,762,881,880,617,585,876,582,612,778,919,782,439,558,450,656,453,142,403,667,805,830,407,601,1166,556,415,526,482,479,550,673,630,579,1105,425,583,723,549,759,190,380,338,316,520,284,551,446,505,341,419,374,394,443,308,205,254,162,258,576,281,377,385,259,213,606,217,402,522,426,18,24,13,10,6,7,4,13,230,262,171,132,392,233,79,8,10,11,14,121,199,89,505,381,145,422,176,290,227,367,590,389,457,478,526,859,753,372,264,6,350,382,19,208,558,377

	
```

```
		  

		5, Position,0,100000,200000,300000,400000,500000,600000,700000,800000,900000,1000000,1100000,1200000,1300000,1400000,1500000,1600000,1700000,1800000,1900000,2000000,2100000,2200000,2300000,2400000,2500000,2600000,2700000,2800000,2900000,3000000,3100000,3200000,3300000,3400000,3500000,3600000,3700000,3800000,3900000,4000000,4100000,4200000,4300000,4400000,4500000,4600000,4700000,4800000,4900000,5000000,5100000,5200000,5300000,5400000,5500000,5600000,5700000,5800000,5900000,6000000,6100000,6200000,6300000,6400000,6500000,6600000,6700000,6800000,6900000,7000000,7100000,7200000,7300000,7400000,7500000,7600000,7700000,7800000,7900000,8000000,8100000,8200000,8300000,8400000,8500000,8600000,8700000,8800000,8900000,9000000,9100000,9200000,9300000,9400000,9500000,9600000,9700000,9800000,9900000,10000000,10100000,10200000,10300000,10400000,10500000,10600000,10700000,10800000,10900000,11000000,11100000,11200000,11300000,11400000,11500000,11600000,11700000,11800000,11900000,12000000,12100000,12200000,12300000,12400000,12500000,12600000,12700000,12800000,12900000,13000000,13100000,13200000,13300000,13400000,13500000,13600000,13700000,13800000,13900000,14000000,14100000,14200000,14300000,14400000,14500000,14600000,14700000,14800000,14900000,15000000,15100000,15200000,15300000,15400000,15500000,15600000,15700000,15800000,15900000,16000000,16100000,16200000,16300000,16400000,16500000,16600000,16700000,16800000,16900000,17000000,17100000,17200000,17300000,17400000,17500000,17600000,17700000,17800000,17900000,18000000,18100000,18200000,18300000,18400000,18500000,18600000,18700000,18800000,18900000,19000000,19100000,19200000,19300000,19400000,19500000,19600000,19700000,19800000,19900000,20000000,20100000,20200000,20300000,20400000,20500000,20600000,20700000,20800000,20900000,21000000,21100000,21200000,21300000,21400000,21500000,21600000,21700000,21800000,21900000,22000000,22100000,22200000,22300000,22400000,22500000,22600000,22700000,22800000,22900000,23000000,23100000,23200000,23300000,23400000,23500000,23600000,23700000,23800000,23900000,24000000,24100000,24200000,24300000,24400000,24500000,24600000,24700000,24800000,24900000,25000000,25100000,25200000,25300000,25400000,25500000,25600000,25700000,25800000,25900000,26000000,26100000,26200000,26300000,26400000,26500000,26600000,26700000,26800000,26900000
5,Count,348,382,344,68,258,646,612,584,719,385,541,322,640,38,348,379,230,405,46,219,378,319,496,406,438,298,78,232,59,160,390,293,267,439,324,451,289,249,401,314,386,216,320,232,216,88,63,232,306,231,306,315,550,398,247,344,655,277,550,653,323,274,430,314,445,226,314,262,350,540,962,374,428,401,587,472,601,596,504,596,577,218,674,642,253,600,622,275,678,470,467,958,696,657,944,651,645,213,566,848,761,684,626,306,800,1091,636,291,646,126,178,155,116,160,179,177,166,142,65,142,129,231,187,187,174,111,131,141,37,61,185,132,146,563,497,447,674,493,632,16,34,22,10,22,0,357,629,717,792,721,784,658,644,672,729,651,479,839,849,609,653,730,306,253,483,534,810,419,3,4,12,7,10,8,23,558,819,563,735,819,542,582,633,296,19,83,666,740,354,15,10,4,4,5,8,6,608,824,419,499,424,422,170,151,406,235,9,6,202,243,195,75,96,11,3,5,257,122,283,727,74,322,8,6,4,30,11,10,211,308,58,6,91,334,269,221,306,248,199,259,428,368,362,370,345,174,389,392,252,322,272,235,529,429,320,483,283,429,293,15,287,160,154,183,407,502,540,213,70,174

	
```


---

 **Details by gene** 

**Here** you can find a tab-separated table.
